# High‐dimensional principal component analysis with heterogeneous missingness

**DOI:** 10.1111/rssb.12550

**Published:** 2022-11-20

**Authors:** Ziwei Zhu, Tengyao Wang, Richard J. Samworth

**Affiliations:** ^1^ Statistical Laboratory University of Cambridge Cambridge UK; ^2^ Department of Statistics University of Michigan Ann Arbor Michigan USA; ^3^ Department of Statistics London School of Economics London UK

**Keywords:** heterogeneous missingness, high‐dimensional statistics, iterative projections, missing data, principal component analysis

## Abstract

We study the problem of high‐dimensional Principal Component Analysis (PCA) with missing observations. In a simple, homogeneous observation model, we show that an existing observed‐proportion weighted (OPW) estimator of the leading principal components can (nearly) attain the minimax optimal rate of convergence, which exhibits an interesting phase transition. However, deeper investigation reveals that, particularly in more realistic settings where the observation probabilities are heterogeneous, the empirical performance of the OPW estimator can be unsatisfactory; moreover, in the noiseless case, it fails to provide exact recovery of the principal components. Our main contribution, then, is to introduce a new method, which we call primePCA, that is designed to cope with situations where observations may be missing in a heterogeneous manner. Starting from the OPW estimator, primePCA iteratively projects the observed entries of the data matrix onto the column space of our current estimate to impute the missing entries, and then updates our estimate by computing the leading right singular space of the imputed data matrix. We prove that the error of primePCA converges to zero at a geometric rate in the noiseless case, and when the signal strength is not too small. An important feature of our theoretical guarantees is that they depend on average, as opposed to worst‐case, properties of the missingness mechanism. Our numerical studies on both simulated and real data reveal that primePCA exhibits very encouraging performance across a wide range of scenarios, including settings where the data are not Missing Completely At Random.

## 1 INTRODUCTION

One of the ironies of working with Big Data is that missing data play an ever more significant role, and often present serious difficulties for analysis. For instance, a common approach to handling missing data is to perform a so‐called *complete‐case analysis* (Little & Rubin, 2019), where we restrict attention to individuals in our study with no missing attributes. When relatively few features are recorded for each individual, one can frequently expect a sufficiently large proportion of complete cases that, under an appropriate missing at random (MAR) hypothesis, a complete‐case analysis may result in only a relatively small loss of efficiency. On the other hand, in high‐dimensional regimes where there are many features of interest, there is often such a small proportion of complete cases that this approach becomes infeasible. As a very simple illustration of this phenomenon, imagine an n×d data matrix in which each entry is missing independently with probability 0.01. When d=5, a complete‐case analysis would result in around 95% of the individuals (rows) being retained, but even when we reach d=300, only around 5% of rows will have no missing entries.

The inadequacy of the complete‐case approach in many applications has motivated numerous methodological developments in the field of missing data over the past 60 years or so, including imputation (Ford, 1983; Rubin, 2004), factored likelihood (Anderson, 1957) and maximum likelihood approaches (Dempster et al., 1977); see, for example, Little & Rubin (2019) for an introduction to the area. Recent years have also witnessed increasing emphasis on understanding the performance of methods for dealing with missing data in a variety of high‐dimensional problems, including sparse regression (Belloni et al., 2017; Loh & Wainwright, 2012), classification (Cai & Zhang, 2018), sparse principal component analysis (Elsener & van de Geer, 2018) and covariance and precision matrix estimation (Loh & Tan, 2018; Lounici, 2014).

In this paper, we study the effects of missing data in one of the canonical problems of high‐dimensional data analysis, namely dimension reduction via Principal Component Analysis (PCA). This is closely related to the topic of *matrix completion*, which has received a great deal of attention in the literature over the last decade or so (Candès et al., 2011; Candès & Plan, 2010; Candès & Recht, 2009; Keshavan et al., 2010; Koltchinskii et al., 2011; Mazumder et al., 2010; Negahban & Wainwright, 2012) for example. There, the focus is typically on accurate recovery of the missing entries, subject to a low‐rank assumption on the signal matrix; by contrast, our focus is on estimation of the principal eigenspaces. Previously proposed methods for low‐dimensional PCA with missing data include non‐linear iterative partial least squares (Wold & Lyttkens, 1969), iterative PCA (Josse & Husson, 2012; Kiers, 1997) and its regularised variant (Josse et al., 2009); see Dray & Josse (2015) for a nice survey and comparative study. More broadly, the R‐miss‐tastic website https://rmisstastic.netlify.com/ provides a valuable resource on methods for handling missing data.

The importance of the problem of high‐dimensional PCA with missing data derives from its wide variety of applications. For instance, in many commercial settings, one may have a matrix of customers and products, with entries recording the number of purchases. Naturally, there will typically be a high proportion of missing entries. Nevertheless, PCA can be used to identify items that distinguish the preferences of customers particularly effectively, to make recommendations to users of products they might like and to summarise efficiently customers' preferences. Later, we will illustrate such an application, on the Million Song Dataset, where we are able to identify particular songs that have substantial discriminatory power for users' preferences as well as other interesting characteristics of the user database. Other potential application areas include health data, where one may seek features that best capture the variation in a population, and where the corresponding principal component scores may be used to cluster individuals into subgroups (that may, for instance, receive different treatment regimens).

To formalise the problem we consider, suppose that the (partially observed) matrix n×d matrix Y is of the form

(1) Y=X+Z,

for independent random matrices X and Z, where X is a low‐rank matrix and Z is a noise matrix with independent and identically distributed entries having zero mean. The low‐rank property of X is encoded through the assumption that it is generated via

(2) $$X=UV_{K}^{⊤},$$

where $V_{K}\in {\mathbb{R}}^{d\times K}$ has orthonormal columns and U is a random n×K matrix (with n>K) having independent and identically distributed rows with mean zero and covariance matrix $\sum_{u}$. Note that when X and Z are independent, the covariance matrix of Y has a K‐spiked structure; such covariance models have been studied extensively in both theory and applications (Cai et al., 2013; Fan et al., 2013; Johnstone & Lu, 2009; Paul, 2007).

We are interested in estimating the column space of $V_{K}$, denoted by $\text{Col}(V_{K})$, which is also the K‐dimensional leading eigenspace of $\sum_{y}:=n^{-1}𝔼(Y^{⊤}Y)$. Cho et al. (2017) considered a different but related model where U in (2) is deterministic, and is not necessarily centred, so that $V_{K}$ is the top K right singular space of 𝔼(Y). (By contrast, in our setting, 𝔼(Y)=0, so the mean structure is uninformative for recovering $V_{K}$.) Their model can be viewed as being obtained from the model (1) and (2) by conditioning on U. In the context of a p‐*homogeneous Missing Completely At Random (MCAR) observation model*, where each entry of Y is observed independently with probability p∈(0,1) (independently of Y), Cho et al. (2017) studied the estimation of $\text{Col}(V_{K})$ by $\text{Col}({\hat{V}}_{K})$, where ${\hat{V}}_{K}$ is a simple estimator formed as the top K eigenvectors of an observed‐proportion weighted (OPW) version of the sample covariance matrix (here, the weighting is designed to achieve approximate unbiasedness). Our first contribution, in Section 2, is to provide a detailed, finite‐sample analysis of this estimator in the model given by (1) and (2) together with a p‐homogeneous MCAR missingness structure, with a noise level of constant order. The differences between the settings necessitate completely different arguments, and reveal in particular a new phenomenon in the form of a phase transition in the attainable risk bound for the sinΘ loss function, that is, the Frobenius norm of the diagonal matrix of the sines of the principal angles between ${\hat{V}}_{K}$ and $V_{K}$. Moreover, we also provide a minimax lower bound in the case of estimating a single principal component, which reveals that this estimator achieves the minimax optimal rate up to a poly‐logarithmic factor.

While this appears to be a very encouraging story for the OPW estimator, it turns out that it is really only the starting point for a more complete understanding of high‐dimensional PCA with missing data. For instance, in the noiseless case, the OPW estimator fails to provide exact recovery of the principal components. Moreover, it is the norm rather than the exception in applications that missingness is *heterogeneous*, in the sense that the probability of observing entries of Y varies (often significantly) across columns. For instance, in recommendation systems, some products will typically be more popular than others, and hence we observe more ratings in those columns. As another example, in meta‐analyses of data from several studies, it is frequently the case that some covariates are common across all studies, while others appear only in a reduced proportion of them. In Section 2.2, we present an example to show that, even with an MCAR structure, PCA algorithms can break down entirely for such heterogeneous observation mechanisms when individual rows of $V_{K}$ can have large Euclidean norm. Intuitively, if we do not observe the interaction between the jth and kth columns of Y, then we cannot hope to estimate the jth or kth rows of $V_{K}$, and this will cause substantial error if these rows of $V_{K}$ contain significant signal. This example illustrates that it is only possible to handle heterogeneous missingness in high‐dimensional PCA with additional structure, and indicates that it is natural to measure the difficulty of the problem in terms of the *incoherence* among the entries of $V_{K}$—that is, the maximum Euclidean norm of the rows of $V_{K}$.

**FIGURE 1:**
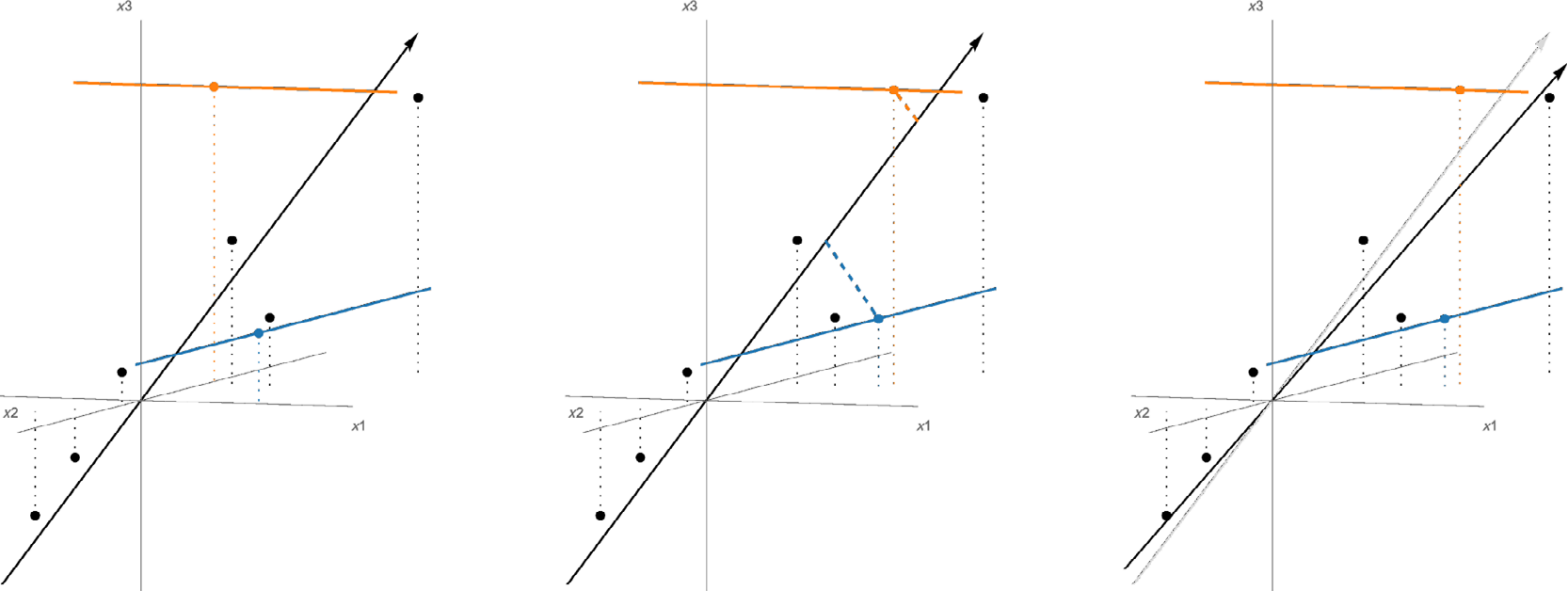
An illustration of the two steps of a single iteration of the primePCA algorithm with d=3 and K=1. Black dots represent fully observed data points, while vertical dotted lines that emanate from them give an indication of their $x_{3}$ coordinate values, as well as their projections onto the $x_{1}$‐$x_{2}$ plane. The $x_{1}$ coordinate of the orange data point and the $x_{2}$ coordinate of the blue data point are unobserved, so the true observations lie on the respective solid lines through those points (which are parallel to the relevant axes). Starting from an input estimate of $V_{K}$ (left), given by the black arrow, we impute the missing coordinates as the closest points on the coloured lines to $V_{K}$ (middle), and then obtain an updated estimate of $V_{K}$ as the leading right singular vector of the imputed data matrix (right, with the old estimate in grey). [Colour figure can be viewed at wileyonlinelibrary.com]

Our main contribution, then, is to propose a new, iterative algorithm, called primePCA (short for projected refinement for imputation of missing entries in Principal Component Analysis), in Section 3, to estimate $V_{K}$, even with heterogeneous missingness. The main initialiser that we study for this algorithm is a modified version of the simple estimator discussed above, where the modification accounts for potential heterogeneity. Each iteration of primePCA projects the observed entries of Y onto the column space of the current estimate of $V_{K}$ to impute missing entries, and then updates our estimate of $V_{K}$ by computing the leading right singular space of the imputed data matrix. An illustration of the two steps of a single iteration of the primePCA algorithm in the case d=3, K=1 is given in Figure 1.

Our theoretical results reveal that in the noiseless setting, that is, Z=0, primePCA achieves exact recovery of the principal eigenspaces (with a geometric convergence rate) when the initial estimator is close to the truth and a sufficiently large proportion of the data are observed. Moreover, we also provide a performance guarantee for the initial estimator, showing that under appropriate conditions it satisfies the desired requirement with high probability, conditional on the observed missingness pattern. Code for our algorithm is available in the R package primePCA (Zhu et al., 2019).

To the best of our knowledge, primePCA is the first method for high‐dimensional PCA that is designed to cope with settings where missingness is heterogeneous. Indeed, the previously mentioned works on high‐dimensional PCA and other high‐dimensional statistical problems with missing data have either focused on a uniform missingness setting or have imposed a lower bound on entrywise observation probabilities, which reduces to this uniform case. In particular, such results fail to distinguish in terms of the performance of their algorithms between a setting where one variable is observed with a very low probability p and all other variables are fully observed, and a setting where all variables are observed with probability p. A key contribution of our work is to account explicitly for the effect of a heterogeneous missingness mechanism, where the estimation error depends on average entrywise missingness rather than worst‐case missingness; see the discussions after Theorem 4 and Proposition 2 below. In Section 4, the empirical performance of primePCA is compared with both that of the initialiser, and a popular method for matrix completion called softImpute (Hastie et al., 2015; Mazumder et al., 2010); we also discuss maximum likelihood approaches implemented via the Expectation–Maximisation (EM) algorithm, which can be used when the dimension is not too high. Our settings include a wide range of signal‐to‐noise ratios (SNRs), as well as Missing Completely At Random, MAR and Missing Not At Random (MNAR) examples (Little & Rubin, 2019; Seaman et al., 2013). These comparisons reveal that primePCA provides highly accurate and robust estimation of principal components, for instance outperforming the softImpute algorithm, even when the latter is allowed access to the oracle choice of regularisation parameter for each dataset. Our analysis of the Million Song Dataset is given in Section 5. In Section 6, we illustrate how some of the ideas in this work may be applied to other high‐dimensional statistical problems involving missing data. Proofs of our main results are deferred to Section A in Appendix S1 (Zhu et al., 2019); auxiliary results and their proofs are given in Section B of Appendix S1.

### 1.1 Notation

For a positive integer T, we write [T]:={1,…,T}. For $v={\left(v_{1},\ldots ,v_{d}\right)}^{⊤}\in {\mathbb{R}}^{d}$ and p∈[1,∞), we define $\|v{\|}_{p}:=(\sum_{j=1}^{d}|v_{j}{|}^{p}){\;}^{1/p}$ and $\left\|v{\|}_{\infty }:=\max_{j\in [d]}|v_{j}\right|$. We let ${𝒮}^{d-1}:=\{u\in {\mathbb{R}}^{d}:\|u{\|}_{2}=1\}$ denote the unit Euclidean sphere in ${\mathbb{R}}^{d}$.

Given $u={\left(u_{1},\ldots ,u_{d}\right)}^{⊤}\in {\mathbb{R}}^{d}$, we write $\text{diag}(u)\in {\mathbb{R}}^{d\times d}$ for the diagonal matrix whose jth diagonal entry is $u_{j}$. We let ${𝕆}^{d_{1}\times d_{2}}$ denote the set of matrices in ${\mathbb{R}}^{d_{1}\times d_{2}}$ with orthonormal columns. For a matrix $A=(A_{ij})\in {\mathbb{R}}^{d_{1}\times d_{2}}$, and p,q∈[1,∞], we write $\|A{\|}_{p}:=(\sum_{i,j}|A_{ij}{|}^{p}){\;}^{1/p}$ if 1≤p<∞ and $\left\|A{\|}_{\infty }:=\max_{i,j}|A_{ij}\right|$ for its entrywise $\ell_{p}$ norm, as well as $\|A{\|}_{p\to q}:=\sup_{\|v{\|}_{p}=1}\|Av{\|}_{q}$ for its p‐to‐q operator norm. We provide special notation for the (Euclidean) operator norm and the Frobenius norm by writing $\|A{\|}_{\text{op}}:=\|A{\|}_{2\to 2}$ and $\|A{\|}_{F}:=\|A{\|}_{2}$ respectively. We also write $\sigma_{j}(A)$ for the jth largest singular value of A, and define its nuclear norm by $\left\|A{\|}_{*}:=\sum_{j=1}^{\min(d_{1},d_{2})}\sigma_{j}(A\right)$. If S⊆[n], we write $A_{S}\in {\mathbb{R}}^{|S|\times d}$ for the matrix obtained by extracting the rows of A that are in S. For $A,B\in {\mathbb{R}}^{d_{1}\times d_{2}}$, the Hadamard product of A and B, denoted A∘B, is defined such that ${\left(A\circ B\right)}_{ij}=A_{ij}B_{ij}$ for any $i\in [d_{1}]$ and $j\in [d_{2}]$.

## 2 THE OPW ESTIMATOR

In this section, we study a simple OPW estimator of the matrix of principal components. To define the estimator, let ${𝒜}_{ij}$ denote the event that the (i,j)th entry $y_{ij}$ of Y is observed. We define the revelation matrix $\Omega =(\omega_{ij})\in {\mathbb{R}}^{n\times d}$ by $\omega_{ij}:=1_{{𝒜}_{ij}}$, and the partially observed data matrix

(3) $$Y_{\Omega }:=Y\circ \Omega .$$

Our observed data are the pair ($Y_{\Omega },\Omega$). Importantly, the fact that we observe Ω allows us to distinguish between observed zeros and missing entries (even though these also appear as zeros in $Y_{\Omega }$). We first consider the simplest possible case, which we refer to as the p‐homogeneous observation model, where entries of the data matrix Y are observed independently and completely at random (i.e., independent of (U,Z)), each with probability p. Thus, $\mathbb{P}({𝒜}_{ij})=p\in (0,1)$ for all i∈[n],j∈[d], and ${𝒜}_{ij}$ and ${𝒜}_{i^{\prime }j^{\prime }}$ are independent for $\left(i,j)\neq (i^{\prime },j^{\prime }\right)$.

For i∈[n], let $y_{i}^{⊤}$ and $\omega_{i}^{⊤}$ denote the ith rows of Y and Ω, respectively, and define ${\tilde{y}}_{i}:=y_{i}\circ \omega_{i}$. Writing $P:=𝔼\omega_{1}\omega_{1}^{⊤}$ and W for its entrywise inverse, we have that under the p‐homogeneous observation model, $P=p^{2}\{1_{d}1_{d}^{⊤}-(1-p^{-1})I_{d}\}$ and $W=p^{-2}\{1_{d}1_{d}^{⊤}-(1-p)I_{d}\}$. Following Lounici (2013, 2014) and Cho et al. (2017), we consider the following weighted sample covariance matrix:

$$G:=(\frac{1}{n}Y_{\Omega }^{⊤}Y_{\Omega })\circ W=(\frac{1}{n}\overset{n}{\underset{i=1}{\sum }}{\tilde{y}}_{i}{\tilde{y}}_{i}^{⊤})\circ W.$$

The reason for including the weight W is to ensure that $𝔼(G|Y)=n^{-1}Y^{⊤}Y$, so that G is an unbiased estimator of $\sum_{y}$. Related ideas appear in the work of Cai & Zhang (2016) on high‐dimensional covariance matrix estimation with missing data; see also Little & Rubin (2019, section 3.4). In practice, p is typically unknown and needs to be estimated. It is therefore natural to consider the following plug‐in estimator $\hat{G}$:

(4) $$\hat{G}=(\frac{1}{n}Y_{\Omega }^{⊤}Y_{\Omega })\circ \hat{W},$$

where $\hat{W}={\hat{p}}^{-2}\{1_{d}1_{d}^{⊤}-(1-\hat{p})I_{d}\}$ and $\hat{p}:={\left(nd\right)}^{-1}\|\Omega {\|}_{1}$ denotes the proportion of observed entries in Y. The OPW estimator of $V_{K}$, denoted ${\hat{V}}_{K}^{\text{OPW}}$, is the d×K matrix formed from the top K eigenvectors of $\hat{G}$.

### 2.1 Theory for homogeneous missingness

We begin by studying the theoretical performance of ${\hat{V}}_{K}^{\text{OPW}}$ in a simple model that will allow us to reveal an interesting phase transition for the problem. For a random vector x taking values in ${\mathbb{R}}^{d}$ and for r≥1, we define its (Orlicz) $\psi_{r}$‐norm and a version that is invariant to invertible affine transformations by

$$\|x{\|}_{\psi_{r}}:=\underset{u\in {𝒮}^{d-1}}{\sup}\underset{q\in \mathbb{N}}{\sup}\frac{(𝔼|u^{⊤}x{|}^{q}){\;}^{1/q}}{q^{1/r}}\;\text{and}\;\|x{\|}_{\psi_{r}^{*}}:=\underset{u\in {𝒮}^{d-1}}{\sup}\frac{\|u^{⊤}(x-𝔼x){\|}_{\psi_{r}}}{{\mathrm{var}}^{1/2}(u^{⊤}x)},$$

respectively. Recall that we say x is *sub‐Gaussian* if $\|x{\|}_{\psi_{2}^{*}}<\infty$.

In this preliminary section, we assume that $\left(Y_{\Omega },\Omega \right)$ is generated according to (1), (2) and (3), where:

(A1) U, Z and Ω are independent;
(A2) U has independent and identically distributed rows $\left(u_{i}:i\in [n]\right)$ with $𝔼u_{1}=0$ and $\|u_{1}{\|}_{\psi_{2}^{*}}\leq \tau$;
(A3) $Z={\left(z_{ij}\right)}_{i\in [n],j\in [d]}$ has independent and identically distributed entries with $𝔼z_{11}=0$, $\mathrm{var}z_{11}=1$ and $\|z_{11}{\|}_{\psi_{2}^{*}}\leq \tau$;
(A4) $\|y_{1j}^{2}{\|}_{\psi_{1}}\leq M$ for all j∈[d];
(A5) Ω has independent Bern(p) entries.

Thus, (A1) ensures that the complete data matrix Y and the revelation matrix Ω are independent; in other words, for now we work in a Missing Completely At Random (MCAR) setting. In a homoscedastic noise model, there is no loss of generality (by a scaling argument) in assuming that each entry of Z has unit variance, as in (A3). In many places in this work, it will be convenient to think intuitively of τ and M in (A2)–(A4) as constants. In particular, if U has multivariate normal rows and Z has normal entries, then we can simply take τ=1. For M, under the same normality assumptions, we have $\left\|y_{1j}^{2}{\|}_{\psi_{1}}=\mathrm{var}(y_{1j}\right)$, so this intuition amounts to thinking of the variance of each component of our data as being of constant order.

A natural measure of the performance of an estimator ${\hat{V}}_{K}$ of $V_{K}$ is given by the Davis–Kahan sinΘ loss

$$L({\hat{V}}_{K},V_{K}):=\frac{1}{\sqrt{2}}\|{\hat{V}}_{K}{\hat{V}}_{K}^{⊤}-V_{K}V_{K}^{⊤}{\|}_{F},$$

(Davis & Kahan, 1970)^1^. Our first theorem controls the risk of the OPW estimator; here and below, we write $\lambda_{k}$ for the kth largest eigenvalue of $\sum_{u}$.

Theorem 1 *Assume* (*A1*)–(*A5*) *and that* n,d≥2, dp≥1. *Write* $R:=\lambda_{1}+1$. *Then there exists a universal constant* C>0 *such that*

(5) $$𝔼L({\hat{V}}_{K}^{\text{OPW}},V_{K})\leq \frac{CK^{1/2}}{\lambda_{K}p}\left\{{\left(\frac{Md(R\tau^{2}p+M\log d)\log^{2}d}{n}\right)}^{1/2}+\frac{Md(\log^{2}d)(\log n)}{n}\right\}.$$

*In particular, if* $n\geq d(\log^{2}d)(\log^{2}n)/(\lambda_{1}p+\log d)$, *then there exists* $C_{M,\tau }>0$, *depending only on* M *and* τ, *such that*

(6) $$𝔼L({\hat{V}}_{K}^{\text{OPW}},V_{K})\leq \frac{C_{M,\tau }}{\lambda_{K}p}{\left(\frac{Kd(\lambda_{1}p+\log d)\log^{2}d}{n}\right)}^{1/2}.$$

Theorem 1 reveals an interesting phase transition phenomenon. Specifically, if the signal strength is large enough that $\lambda_{1}\geq p^{-1}\log d$, then we should regard np as the effective sample size, as might intuitively be expected. On the other hand, if $\lambda_{1}<p^{-1}\log d$, then the estimation problem is considerably more difficult and the effective sample size is of order $np^{2}$. In fact, by inspecting the proof of Theorem 1, we see that in the high signal case, it is the difficulty of estimating the diagonal entries of $\sum_{y}$ that drives the rate, while when the signal strength is low, the bottleneck is the challenge of estimating the off‐diagonal entries. By comparing (6) with the minimax lower bound result in Theorem 2 below, we see that this phase transition phenomenon is an inherent feature of this estimation problem, rather than an artefact of the proof techniques we used to derived the upper bound.

The condition $n\geq d(\log^{2}d)(\log^{2}n)/(\lambda_{1}p+\log d)$ in Theorem 1 is reasonable given the scaling requirement for consistency of the empirical eigenvectors (Johnstone & Lu, 2009; Shen et al., 2016; Wang & Fan, 2017). Indeed, Shen et al. (2016, Theorem 5.1) show that when $\lambda_{1}\gg 1$, the top eigenvector of the sample covariance matrix estimator is consistent if and only if $d/(n\lambda_{1})\to 0$. If we regard np as the effective sample size in our missing data PCA problem, then it is a sensible analogy to assume that $d/(np\lambda_{1})\to 0$ here, which implies that the condition $n\geq d(\log^{2}d)(\log^{2}n)/(\lambda_{1}p+\log d)$ holds for large n, up to poly‐logarithmic factors.

As mentioned in the introduction, Cho et al. (2017) considered the different but related problem of singular space estimation in a model in which Y=Θ+Z, where Θ is a matrix of the form $UV_{K}^{⊤}$ for a *deterministic* matrix U, whose rows are not necessarily centred. In this setting, $V_{K}$ is the matrix of top K right singular vectors of Θ, and the same estimator ${\hat{V}}_{K}$ can be applied. An important distinction is that, when the rows of U are not centred and the entries of Θ are of comparable magnitude, $\|\Theta {\|}_{F}$ is of order $\sqrt{nd}$, so when K is regarded as a constant, it is natural to think of the singular values of Θ as also being of order $\sqrt{nd}$. Indeed, this is assumed in Cho et al. (2017). On the other hand, in our model, where the rows of U have mean zero, assuming that the eigenvalues are of order $\sqrt{nd}$ would amount to an extremely strong requirement, essentially restricting attention to very highly spiked covariance matrices. Removing this condition in Theorem 1 requires completely different arguments.

In order to state our minimax lower bound, we let ${𝒫}_{n,d}(\lambda_{1},p)$ denote the class of distributions of pairs $\left(Y_{\Omega },\Omega \right)$ satisfying (A1), (A2), (A3) and (A5) with K=1. Since we are now working with vectors instead of matrices, we write v in place of $V_{1}$.

Theorem 2 *There exists a universal constant* c>0 *such that*

$$\underset{\hat{v}}{\inf}\underset{P\in {𝒫}_{n,d}(\lambda_{1},p)}{\sup}{𝔼}_{P}L(\hat{v},v)\geq c\min\left\{\frac{1}{\lambda_{1}p}{\left(\frac{d(\lambda_{1}p+1)}{n}\right)}^{1/2},1\right\},$$

*where the infimum is taken over all estimators* $\hat{v}=\hat{v}(Y_{\Omega },\Omega )$ *of* v.

Theorem 2 reveals that ${\hat{V}}_{1}^{\text{OPW}}$ in Theorem 1 achieves the minimax optimal rate of estimation up to a poly‐logarithmic factor when M and τ are regarded as constants.

### 2.2 Heterogeneous observation mechanism

A key assumption of the theory in Section 2.1, which allowed even a very simple estimator to perform well, was that the missingness probability was homogeneous across the different entries of the matrix. On the other hand, the aim of this sub‐section is to show that the situation changes dramatically once the data can be missing heterogeneously.

To this end, consider the following example. Suppose that ω is equal to ${\left(1,0,1,\ldots ,1\right)}^{⊤}$ or ${\left(0,1,1,\ldots ,1\right)}^{⊤}$ with equal probability, so that

$$P=𝔼\omega \omega^{⊤}=\left(\begin{array}{ccccc} 1/2 & 0 & 1/2 & \ldots & 1/2\\ 0 & 1/2 & 1/2 & \ldots & 1/2\\ 1/2 & 1/2 & 1 & \ldots & 1\\ \vdots & \vdots & \vdots & \ddots & \vdots \\ 1/2 & 1/2 & 1 & \ldots & 1 \end{array}\right)\in {\mathbb{R}}^{d\times d}.$$

In other words, for each i∈[n], we observe precisely one of the first two entries of $y_{i}$, together with all of the remaining (d−2) entries. Let $\sum =I_{d}+\alpha \alpha^{⊤}$, where $\alpha ={\left(2^{-1/2},2^{-1/2},0,\ldots ,0\right)}^{⊤}\in {\mathbb{R}}^{d}$, and $\sum^{\prime }=I_{d}+\alpha^{\prime }{\left(\alpha^{\prime }\right)}^{⊤}$, where $\alpha^{\prime }={\left(2^{-1/2},-2^{-1/2},0,\ldots ,0\right)}^{⊤}\in {\mathbb{R}}^{d}$. Suppose that $y∼N_{d}(0,\sum )$ and let $\tilde{y}:=y\circ \omega$, and similarly assume that $y^{\prime }∼N_{d}(0,\sum^{\prime })$ and set ${\tilde{y}}^{\prime }:=y^{\prime }\circ \omega$. Then $\left(\tilde{y},\omega \right)$ and $\left({\tilde{y}}^{\prime },\omega \right)$ are identically distributed. However, the leading eigenvectors of ∑ and $\sum^{\prime }$ are respectively α and $\alpha^{\prime }$, which are orthogonal!

Thus, it is impossible to simultaneously estimate consistently the leading eigenvector of both ∑ and $\sum^{\prime }$ from our observations. We note that it is the disproportionate weight of the first two coordinates in the leading eigenvector, combined with the failure to observe simultaneously the first two entries in the data, that makes the estimation problem intractable in this example. The understanding derived from this example motivates us to seek bounds on the error in high‐dimensional PCA that depend on an incoherence parameter $\mu :={\left(d/K\right)}^{1/2}\|V_{K}{\|}_{2\to \infty }\in [1,{\left(d/K\right)}^{1/2}]$. The intuition here is that the maximally incoherent case is where each column of $V_{K}$ is a unit vector proportional to a vector whose entries are either 1 or −1, in which case $\|V_{K}{\|}_{2\to \infty }={\left(K/d\right)}^{1/2}$ and μ=1. On the other hand, in the worst case, when the columns of $V_{K}$ are the first K standard basis vectors in ${\mathbb{R}}^{d}$, we have $\mu ={\left(d/K\right)}^{1/2}$. Bounds involving incoherence have appeared previously in the literature on matrix completion (e.g., Candès & Plan, 2010; Keshavan et al., 2010), but for a different reason. There, the purpose is to control the principal angles between the true right singular space and the standard basis, which yields bounds on the number of observations required to infer the missing entries of the matrix. In our case, the incoherence condition controls the extent to which the loadings of the principal components of interest are concentrated in any single coordinate, and therefore the extent to which significant estimation error in a few components of the leading eigenvectors can affect the overall statistical performance. In the intractable example above, $\mu ={\left(d/2\right)}^{1/2}$, and with such a large value of μ, heavy corruption from missingness in only a few entries spoils any chance of consistent estimation.

## 3 OUR NEW ALGORITHM FOR PCA WITH MISSING ENTRIES

We are now in a position to introduce and analyse our iterative algorithm primePCA to estimate $\text{Col}(V_{K})$, the principal eigenspace of the covariance matrix $\sum_{y}$. The basic idea is to iterate between imputing the missing entries of the data matrix $Y_{\Omega }$ using a current (input) iterate ${\hat{V}}_{K}^{\left(\text{in}\right)}$, and then applying a singular value decomposition (SVD) to the completed data matrix. More precisely, for i∈[n], we let ${𝒥}_{i}$ denote the indices for which the corresponding entry of $y_{i}$ is observed, and regress the observed data ${\tilde{y}}_{i,{𝒥}_{i}}=y_{i,{𝒥}_{i}}$ on ${\left({\hat{V}}_{K}^{\left(\text{in}\right)}\right)}_{{𝒥}_{i}}$ to obtain an estimate ${\hat{u}}_{i}$ of the ith row of U. This is natural in view of the data generating mechanism $y_{i}=V_{K}u_{i}+z_{i}$. We then use ${\hat{y}}_{i,{𝒥}_{i}^{c}}:={\left({\hat{V}}_{K}^{\left(\text{in}\right)}\right)}_{{𝒥}_{i}^{c}}{\hat{u}}_{i}$ to impute the missing values $y_{i,{𝒥}_{i}^{c}}$, retain the original observed entries as ${\hat{y}}_{i,{𝒥}_{i}}:={\tilde{y}}_{i,{𝒥}_{i}}$, and set our next (output) iterate ${\hat{V}}_{K}^{\left(\text{out}\right)}$ to be the top K right singular vectors of the imputed matrix $\hat{Y}:={\left({\hat{y}}_{1},\ldots ,{\hat{y}}_{n}\right)}^{⊤}$. To motivate this final choice, observe that when Z=0, we have rank(Y)=K; we therefore have the SVD $Y=L\Gamma R^{⊤}$, where $L\in {𝕆}^{n\times K},R\in {𝕆}^{d\times K}$ and $\Gamma \in {\mathbb{R}}^{K\times K}$ is diagonal with positive diagonal entries. This means that $R=V_{K}U^{⊤}L\Gamma^{-1}$, so the column spaces of R and $V_{K}$ coincide. For convenience, pseudocode of a single iteration of refinement in this algorithm is given in Algorithm 1.

Algorithm 1 **refine $\left(K,{\hat{V}}_{K}^{\left(\text{in}\right)},\Omega ,Y_{\Omega }\right)$, a single step of refinement of current iterate ${\hat{V}}_{K}^{\left(\text{in}\right)}$
**

1

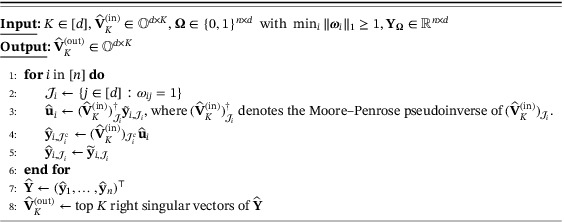

We now seek to provide formal justification for Algorithm 1. The recursive nature of the primePCA algorithm induces complex relationships between successive iterates, so to facilitate theoretical analysis, we will impose some conditions on the underlying data generating mechanism that may not hold in situations where we would like to apply to algorithm. Nevertheless, we believe that the analysis provides considerable insight into the performance of the primePCA algorithm, and these are discussed extensively below; moreover, our simulations in Section 4 consider settings both within and outside the scope of our theory, and confirm its attractive and robust numerical performance.

In addition to the loss function L, it will be convenient to define a slightly different notion of distance between subspaces. For any $V,\tilde{V}\in {𝕆}^{d\times K}$, we let $W_{1}DW_{2}^{⊤}$ be an SVD of ${\tilde{V}}^{⊤}V$. The *two‐to‐infinity distance* between $\tilde{V}$ and V is then defined to be

$$𝒯(\tilde{V},V):=\|\tilde{V}-VW_{2}W_{1}^{⊤}{\|}_{2\to \infty }.$$

We remark that the definition of $𝒯(\tilde{V},V)$ does not depend on our choice of SVD and that $𝒯(\tilde{V},V)=𝒯(\tilde{V}O_{1},VO_{2})$ for any $O_{1},O_{2}\in {𝕆}^{K\times K}$, so that 𝒯 really represents a distance between the subspaces spanned by $\tilde{V}$ and V. In fact, there is a sense in which the change‐of‐basis matrix $W_{2}W_{1}^{⊤}$ tries to align the columns of V as closely as possible with those of $\tilde{V}$; more precisely, if we change the norm from the two‐to‐infinity operator norm to the Frobenius norm, then $W_{2}W_{1}^{⊤}$ uniquely solves the so‐called *Procrustes problem* (Schönemann, 1966):

(7) $$W_{2}W_{1}^{⊤}=\underset{W\in {𝕆}^{K\times K}}{\text{argmin}}{\left\|\tilde{V}-VW\right\|}_{F}.$$

The following proposition considers the noiseless setting Z=0, and shows that, for any estimator ${\hat{V}}_{K}^{\left(\text{in}\right)}$ that is close to $V_{K}$, a single iteration of refinement in Algorithm 1 contracts the two‐to‐infinity distance between their column spaces, under appropriate conditions. We define $\Omega^{c}:=1_{d}1_{d}^{⊤}-\Omega$.

Proposition 1 *Let* ${\hat{V}}_{K}^{\left(\text{out}\right)}:=\text{refine}(K,{\hat{V}}_{K}^{\left(\text{in}\right)},\Omega ,Y_{\Omega })$ *as in Algorithm* 1 *and further let* $\Delta :=𝒯({\hat{V}}_{K}^{\left(\text{in}\right)},V_{K})$. *We assume that* $\min_{i\in [n]}\|\omega_{i}{\|}_{1}>K$ *and that* $\min_{i\in [n]}\frac{d^{1/2}\sigma_{K}({\left({\hat{V}}_{K}^{\left(\text{in}\right)}\right)}_{{𝒥}_{i}})}{|{𝒥}_{i}{|}^{1/2}}\geq 1/\sigma_{*}>0$. *Suppose that* Z=0 *and that the SVD of* Y *is of the form* $L\Gamma R^{⊤}$, *where* $\|L{\|}_{2\to \infty }\leq \mu {\left(K/n\right)}^{1/2}$ *and* $\|R{\|}_{2\to \infty }\leq \mu {\left(K/d\right)}^{1/2}$ *for some* μ≥1. *Then there exist* $c_{1},C>0$, *depending only on* $\sigma_{*}$, *such that whenever*

(*i*) $\Delta \leq \frac{c_{1}\sigma_{K}(\Gamma )}{K^{2}\mu^{4}\sigma_{1}(\Gamma )\sqrt{d}}$,
(*ii*) $\rho :=\frac{CK^{2}\mu^{4}\sigma_{1}(\Gamma )\|\Omega^{c}{\|}_{1\to 1}}{\sigma_{K}(\Gamma )n}<1$,

*we have that*

$$𝒯({\hat{V}}_{K}^{\left(\text{out}\right)},V_{K})\leq \rho \Delta .$$

In order to understand the main conditions of Proposition 1, it is instructive to consider the case K=1, as was illustrated in Figure 1, and initially to think of μ as a constant. In that case, condition (i) asks that the absolute value of every component of the difference between the vectors ${\hat{V}}_{1}^{\left(\text{in}\right)}$ and $V_{1}$ is $O(d^{-1/2})$; for intuition, if two vectors are uniformly distributed on ${𝒮}^{d-1}$, then each of their $\ell_{\infty }$ norms is $O_{p}(d^{-1/2}\log^{1/2}d)$; in other words, we only ask that the initialiser is very slightly better than a random guess. In Condition (ii), ρ being less than 1 is equivalent to the proportion of missing data in each column being less than $1/(C^{\prime }\mu^{4})$ (where $C^{\prime }$ again depends only on $\sigma_{*})$, and the conclusion is that the refine step contracts the initial two‐to‐infinity distance from $V_{K}$ by at least a factor of ρ. In the noiseless setting of Proposition 1, the matrix R of right singular vectors of Y has the same column span (and hence the same two‐to‐infinity norm) as $V_{K}$. We can therefore gain some intuition about the scale of μ by considering the situation where $V_{K}$ is uniformly distributed on ${𝕆}^{d\times K}$, so in particular, the columns of $V_{K}$ are uniformly distributed on ${𝒮}^{d-1}$. By Vershynin (2018, Theorem 5.1.4), we deduce that $\left\|V_{K}{\|}_{2\to \infty }=O_{p}(\sqrt{\frac{K\log d}{d}}\right)$. On the other hand, when the distribution of U is invariant under left multiplication by an orthogonal matrix (e.g. if U has independent and identically distributed Gaussian rows), then L is distributed uniformly on ${𝕆}^{n\times K}$. Arguing as above, we see that, with high probability, we may take $\mu ≲\max(\sqrt{\log n},\sqrt{\log d})$. This calculation suggests that we do not lose too much by thinking of μ as a constant (or at most, growing very slowly with n and d).

To apply Proposition 1, we also require conditions on $\min_{i\in [n]}\|\omega_{i}{\|}_{1}$ and $\sigma_{*}$. In practice, if either of these conditions is not satisfied, we first perform a screening step that restricts attention to a set of row indices for which the data contain sufficient information to estimate the K principal components. This screening step is explicitly accounted for in Algorithm 2, as well as in the theory that justifies it. An alternative would be to seek to weight rows according to their utility for principal component estimation, but it seems difficult to implement this in a principled way that facilitates formal justification.

Algorithm 2 **primePCA, an iterative algorithm for estimating $V_{K}$ given initialiser ${\hat{V}}_{K}^{\left(0\right)}$
**

1

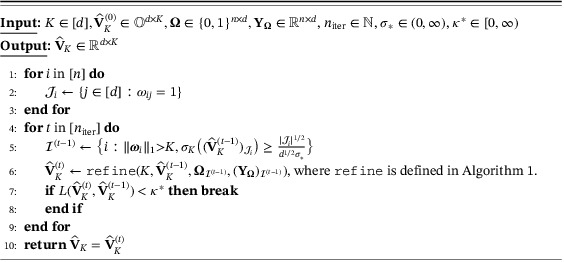

Algorithm 2 provides pseudocode for the iterative primePCA algorithm, given an initial estimator ${\hat{V}}_{K}^{\left(0\right)}$. The iterations continue until either we hit the convergence threshold $\kappa^{*}$ or the maximum iteration number $n_{\text{iter}}$. Theorem 3 below guarantees that, in the noiseless setting of Proposition 1, the primePCA estimator converges to $V_{K}$ at a geometric rate.

Theorem 3 *For* $t\in [n_{\text{iter}}]$, *let* ${\hat{V}}_{K}^{\left(t\right)}$ *be the* tth *iterate of Algorithm* 2 *with input* K, ${\hat{V}}_{K}^{\left(0\right)}$, $\Omega \in {\left\{0,1\right\}}^{n\times d}$, $Y_{\Omega }\in {\mathbb{R}}^{n\times d}$, $n_{\text{iter}}\in \mathbb{N}$, $\sigma_{*}\in (0,\infty )$ *and* $\kappa^{*}=0$. *Write* $\Delta :=𝒯({\hat{V}}_{K}^{\left(0\right)},V_{K})$, *and let*

$$ℐ:=\left\{i:\|\omega_{i}{\|}_{1}>K,\sigma_{K}({\left(V_{K}\right)}_{{𝒥}_{i}})\geq \frac{|{𝒥}_{i}{|}^{1/2}}{d^{1/2}\sigma_{*}}\right\},$$

*where* ${𝒥}_{i}:=\{j:\omega_{ij}=1\}$. *Suppose that* Z=0 *and that the SVD of* $Y_{ℐ}$ *is of the form* $L\Gamma R^{⊤}$, *where* $\|L{\|}_{2\to \infty }\leq \mu {\left(K/n\right)}^{1/2}$ *and* $\|R{\|}_{2\to \infty }\leq \mu {\left(K/d\right)}^{1/2}$ *for some* μ≥1. *Assume that*

$$\epsilon :=\min\left\{\left|\frac{\sigma_{K}({\left(V_{K}\right)}_{{𝒥}_{i}})d^{1/2}}{|{𝒥}_{i}{|}^{1/2}}-\frac{1}{\sigma_{*}}\right|:i\in [n],\|\omega_{i}{\|}_{1}>K\right\}>0.$$

*Then there exist* $c_{1},C>0$, *depending only* $\sigma_{*}$ and ϵ, *such that whenever*

(*i*) $\Delta \leq \frac{c_{1}\sigma_{K}(\Gamma )}{K^{2}\mu^{4}\sigma_{1}(\Gamma )\sqrt{d}}$,
(*ii*) $\rho :=\frac{CK^{2}\mu^{4}\sigma_{1}(\Gamma )\|\Omega_{ℐ}^{c}{\|}_{1\to 1}}{\sigma_{K}(\Gamma )|ℐ|}<1$,

*we have that for every* $t\in [n_{\text{iter}}]$,

$$𝒯({\hat{V}}_{K}^{\left(t\right)},V_{K})\leq \rho^{t}\Delta .$$

The condition that ϵ>0 amounts to the very mild assumption that the algorithmic input $\sigma_{*}$ is not exactly equal to any element of the set $\left\{\frac{|{𝒥}_{i}{|}^{1/2}}{\sigma_{K}({\left(V_{K}\right)}_{{𝒥}_{i}})d^{1/2}}:i\in [n],\|\omega_{i}{\|}_{1}>K\right\}$, though the conditions on $c_{1}$ and C become milder as ϵ increases.

### 3.1 Initialisation

Theorem 3 provides a general guarantee on the performance of primePCA, but relies on finding an initial estimator ${\hat{V}}_{K}^{\left(0\right)}$ that is sufficiently close to the truth $V_{K}$. The aim of this sub‐section, then, is to propose a simple initialiser and show that it satisfies the requirement of Theorem 3 with high probability, conditional on the missingness pattern.

Consider the following modified weighted sample covariance matrix

(8) $$\tilde{G}:=\frac{1}{n}\overset{n}{\underset{i=1}{\sum }}{\tilde{y}}_{i}{\tilde{y}}_{i}^{⊤}\circ \tilde{W},$$

where for any j,k∈[d],

(9) $${\tilde{W}}_{jk}:=\left\{\begin{array}{cc} \frac{n}{\sum_{i=1}^{n}\omega_{ij}\omega_{ik}}\; & \text{if}\;\overset{n}{\underset{i=1}{\sum }}\omega_{ij}\omega_{ik}>0,\\ 0,\; & \text{otherwise}. \end{array}\right.$$

Here, the matrix $\tilde{W}$ replaces $\hat{W}$ in (4) because, unlike in Section 2.1, we no longer wish to assume homogeneous missingness. We take as our initial estimator of $V_{K}$ the matrix of top K eigenvectors of $\tilde{G}$, denoted ${\tilde{V}}_{K}$. Theorem 4 below studies the performance of this initialiser, in terms of its two‐to‐infinity norm error, and provides sufficient conditions for us to be able to apply Theorem 3. In particular, it ensures that the initialiser is reasonably well‐aligned with the target $V_{K}$. We write ${\mathbb{P}}^{\Omega }$ and ${𝔼}^{\Omega }$ for probabilities and expectations conditional on Ω.

Theorem 4 *Assume (A1)*–*(A4) and that* n,d≥2. *Suppose further that* $\|V_{K}{\|}_{2\to \infty }\leq \mu {\left(K/d\right)}^{1/2}$, that $\sum_{i=1}^{n}\omega_{ij}\omega_{ik}>0$ *for all* j,k *and let* $R:=\lambda_{1}+1$. *Then there exist* $c_{M,\tau },C_{M,\tau }>0$, *depending only on* M *and* τ, *such that for every* ξ>2, if

(10) $$\lambda_{K}>c_{M,\tau }\left\{{\left(\frac{\max(\|\tilde{W}{\|}_{1},R\|\tilde{W}{\|}_{1\to 1})\xi \log d}{n}\right)}^{1/2}+\frac{\xi \|\tilde{W}{\|}_{F}\log^{2}d}{n}\right\},$$

*then*

$$\begin{array}{lll} {\mathbb{P}}^{\Omega }\left\{𝒯({\tilde{V}}_{K},V_{K})\geq \frac{C_{M,\tau }K\mu^{2}R^{1/2}}{\lambda_{K}}\left(\frac{K}{d^{1/2}}+\frac{1}{\lambda_{K}}\right)\left(\frac{\xi^{1/2}\|\tilde{W}{\|}_{\infty \to \infty }^{1/2}\log^{1/2}d}{n^{1/2}}+\frac{\xi \|\tilde{W}{\|}_{2\to \infty }\log d}{n}\right)\right\}\; & & \\ \;\;\;\;\;\;\;\leq 2(e^{K\log5}+K+4)d^{-(\xi -1)}+2d^{-(\xi -2)}.\; & & \end{array}$$

*As a consequence, writing*

$$𝒜:=\left\{\frac{\sigma_{K}(Y_{ℐ})}{\sigma_{1}(Y_{ℐ})}>\frac{C_{M,\tau }K^{3}\mu^{6}R^{1/2}}{c_{1}\lambda_{K}}\left(1+\frac{d^{1/2}}{K\lambda_{K}}\right)\left(\frac{\xi^{1/2}\|\tilde{W}{\|}_{\infty \to \infty }^{1/2}\log^{1/2}d}{n^{1/2}}+\frac{\xi \|\tilde{W}{\|}_{2\to \infty }\log d}{n}\right)\right\},$$

*where* ℐ and $c_{1}$ *are as in Theorem* 3, *we have that*

$${\mathbb{P}}^{\Omega }\left(𝒯({\tilde{V}}_{K},V_{K})>\frac{c_{1}\sigma_{K}(Y_{ℐ})}{K^{2}\sigma_{1}(Y_{ℐ})d^{1/2}}\right)\leq 2(e^{K\log5}+K+4)d^{-(\xi -1)}+2d^{-(\xi -2)}+{\mathbb{P}}^{\Omega }({𝒜}^{c}).$$

The first part of Theorem 4 provides a general probabilistic upper bound for $𝒯({\tilde{V}}_{K},V_{K})$, after conditioning on the missingness pattern. This allows us, in the second part, to provide a guarantee on the probability with which ${\tilde{V}}_{K}$ is a good enough initialiser for Theorem 3 to apply. For intuition regarding ${\mathbb{P}}^{\Omega }({𝒜}^{c})$, consider the MCAR setting with $p_{jk}:=𝔼(\omega_{1j}\omega_{1k})$ for j,k∈[d]. In that case, by Lemma 6, typical realisations of $\tilde{W}$ have $\|\tilde{W}{\|}_{\infty \to \infty }\leq 2\max_{j\in [d]}\sum_{k\in [d]}p_{jk}^{-1}$ and $\|\tilde{W}{\|}_{2\to \infty }\leq 2\max_{j\in [d]}(\sum_{k\in [d]}p_{jk}^{-2}){\;}^{1/2}$ when $\sum_{j,k\in [d]}e^{-np_{jk}/8}$ is small. In particular, when $n\min_{j,k\in [d]}p_{jk}\geq \log d$, we expect ${\mathbb{P}}^{\Omega }({𝒜}^{c})$ to be small when $\lambda_{1}$ and $\lambda_{K}$ are both of the same order, and grow faster than

$$\max\left\{{\left(\frac{d\log d}{n}\underset{j\in [d]}{\max}\overset{d}{\underset{k=1}{\sum }}\frac{1}{p_{jk}}\right)}^{1/3},\frac{\log d}{n}\underset{j\in [d]}{\max}\overset{d}{\underset{k=1}{\sum }}\frac{1}{p_{jk}}\right\}.$$

As a special case, in the p‐homogeneous model where $p_{jk}=p^{2}1_{\left\{j\neq k\right\}}+p1_{\left\{j=k\right\}}$ for j,k∈[d], the requirement on $\lambda_{K}$ above is that it should grow faster than $\max\{{\left(\frac{d^{2}\log d}{np^{2}}\right)}^{1/3},\frac{d\log d}{np^{2}}\}$.

One of the attractions of our analysis is the fact that we are able to provide bounds that only depend on entrywise missingness probabilities in an average sense, as opposed to worst‐case missingness probabilities. The refinements conferred by such bounds are particularly important when the missingness mechanism is heterogeneous, as typically encountered in practice. The averaging of missingness probabilities can be partially seen in Theorem 4, since $\|\tilde{W}{\|}_{\infty \to \infty }$ and $\|\tilde{W}{\|}_{2\to \infty }$ depend only on the $\ell_{1}$ and $\ell_{2}$ norms of each row of $\tilde{W}$, but is even more evident in the proposition below, which gives a probabilistic bound on the original sinΘ distance between ${\tilde{V}}_{K}$ and $V_{K}$.

Proposition 2 *Assume the same conditions as in Theorem* 4. *Then there exists a universal constant* C>0 *such that for any* ξ>1, *if*

(11) $$\lambda_{K}>C\left\{{\left(\frac{M\tau^{2}R\|\tilde{W}{\|}_{1\to 1}\xi \log d}{n}\right)}^{1/2}+\frac{M\|\tilde{W}{\|}_{\text{op}}\xi \log^{2}d}{n}\right\},$$

*then*

$$\begin{array}{ll} & {\mathbb{P}}^{\Omega }\left\{L({\tilde{V}}_{K},V_{K})\geq \frac{2^{9/2}eK\tau \mu }{\lambda_{K}}{\left(\frac{MR}{d}\right)}^{1/2}\left(\frac{\xi^{1/2}\|\tilde{W}{\|}_{1}^{1/2}\log^{1/2}d}{n^{1/2}}+\frac{\xi \|\tilde{W}{\|}_{F}\log d}{n}\right)\right\}\\ & \;\leq (2K+4)d^{-(\xi -1)}. \end{array}$$

In this bound, we see that $L({\tilde{V}}_{K},V_{K})$ only depends on $\tilde{W}$ through the entrywise $\ell_{1}$ and $\ell_{2}$ norms of the whole matrix. Lemma 6 again provides probabilistic control of these norms under the p‐homogeneous missingness mechanism. In general, if the rows of Ω are independent and identically distributed, but different covariates are missing with different probabilities, then off‐diagonal entries of $\tilde{W}$ will concentrate around the reciprocals of the simultaneous observation probabilities of pairs of covariates. As such, for a typical realisation of Ω, our bound in Proposition 2 depends only on the harmonic averages of these simultaneous observation probabilities and their squares. Such an averaging effect ensures that our method is effective in a much wider range of heterogeneous settings than previously allowed in the literature.

### 3.2 Weakening the missingness proposition condition for contraction

Theorem 3 provides a geometric contraction guarantee for the primePCA algorithm in the noiseless case. The price we pay for this strong conclusion, however, is a strong condition on the proportion of missingness that enters the contraction rate parameter ρ through $\|\Omega_{ℐ}^{c}{\|}_{1\to 1}$; indeed in an asymptotic framework where the incoherence parameter μ grows with the sample size and/or dimension, the proportion of missingness would need to vanish asymptotically. Therefore, to complement our earlier theory, we present below Proposition 3 and Corollary 1. Proposition 3 is an analogue of the deterministic Proposition 1 in that it demonstrates that a single iteration of the primePCA algorithm yields a contraction provided that the input ${\hat{V}}_{K}^{\left(\text{in}\right)}$ is sufficiently close to $V_{K}$. The two main differences are first that the contraction is in terms of a Procrustes‐type loss (see the discussion around (7)), which turns out to be convenient for Corollary 1; and second, the bound depends only on the incoherence of the matrix $V_{K}$, and not on the corresponding quantity for U.

Proposition 3 *Let* ${\hat{V}}_{K}^{\left(\text{in}\right)}\in {𝕆}^{d\times K}$, *let* $O:={\text{argmin}}_{\tilde{O}\in {𝕆}^{K\times K}}\|{\hat{V}}_{K}^{\left(\text{in}\right)}-V_{K}\tilde{O}{\|}_{F}$ *and let* $\Xi :={\hat{V}}_{K}^{\left(\text{in}\right)}-V_{K}O$. *Fix* $U\in {\mathbb{R}}^{n\times K}$ and $V_{K}\in {𝕆}^{d\times K}$ *with* $\|V_{K}{\|}_{2\to \infty }\leq \mu {\left(K/d\right)}^{1/2}$, *and let* $Y:=UV_{K}^{⊤}$, *with* $c:=\sigma_{K}(Y)/\|Y{\|}_{F}$. *Suppose that* $\kappa_{1},\kappa_{2},\kappa_{3}>0$ *are such that for every* i∈[n],

(12) $$\frac{\|V_{{𝒥}_{i},K}^{⊤}\Xi_{{𝒥}_{i}}{\|}_{\text{op}}}{\left|{𝒥}_{i}\right|}\leq \frac{\|\Xi {\|}_{\text{op}}^{2}}{d}+\kappa_{1}\frac{\mu \|\Xi {\|}_{\text{op}}}{d^{3/2}},\;\;\frac{\|\Xi_{{𝒥}_{i}}{\|}_{\text{op}}^{2}}{\left|{𝒥}_{i}\right|}\leq \kappa_{2}\frac{\|\Xi {\|}_{\text{op}}^{2}}{d},\;\;\|\Xi_{{𝒥}_{i}^{c}}{\|}_{\text{op}}^{2}\leq \kappa_{3}\|\Xi {\|}_{\text{op}}^{2}.$$

*Assume further that*

(13) $$\|\Xi {\|}_{\text{op}}\leq \min\{{\left(\frac{c}{4\overset{2}{\underset{*}{\sigma }}(\kappa_{1}+\kappa_{2})}\right)}^{1/2},\frac{c}{4\mu \kappa_{1}\sigma_{*}^{2}K}{\left(\frac{d}{\log K}\right)}^{1/2}\}.$$

*Then the output* ${\hat{V}}_{K}^{\left(\text{out}\right)}:=\text{refine}(K,{\hat{V}}_{K}^{\left(\text{in}\right)},\Omega ,Y_{\Omega })$ *of Algorithm* 1 *satisfies*

$$\|{\hat{V}}_{K}^{\left(\text{out}\right)}-V_{K}\hat{O}{\|}_{\text{op}}\leq \frac{16\|\Xi {\|}_{\text{op}}}{c}\{\sigma_{*}^{2}(\kappa_{1}+\kappa_{2})\|\Xi {\|}_{\text{op}}+\sigma_{*}^{2}\kappa_{1}\mu K{\left(\frac{\log K}{d}\right)}^{1/2}+\kappa_{3}^{1/2}(1+\frac{c}{2})\},$$

*where* $\hat{O}:={\text{argmin}}_{\tilde{O}\in {𝕆}^{K\times K}}\|{\hat{V}}_{K}^{\left(\text{out}\right)}-V_{K}\tilde{O}{\|}_{F}\in {𝕆}^{K\times K}$.

Interestingly, the proof of Proposition 3 proceeds in a very different fashion from that of Proposition 1. The key step is to bound the discrepancy between the principal components of the imputed data matrix $\hat{Y}$ in Algorithm 1 and $V_{K}$ using a modified version of Wedin's theorem (Wang, 2016). To achieve the desired contraction rate, instead of viewing the true data matrix Y as the reference matrix when calculating the perturbation, we choose a different reference matrix $\tilde{Y}$ with the same top K right singular space as Y but which is closer to $\hat{Y}$ in terms of the Frobenius norm. Such a reference shift sharpens the eigenspace perturbation bound.

The contraction rate in Proposition 3 is a sum of three terms, the first two of which are small provided that $\|\Xi {\|}_{\text{op}}$ is small and d is large respectively. On the other hand, the final term is small provided that no small subset of the rows of Ξ contributes excessively to its operator norm. For different missingness mechanisms, such a guarantee would need to be established probabilistically on a case‐by‐case basis; in Corollary 1 we illustrate how this can be done to achieve a high probability contraction in the simplest missingness model. Importantly, the proportion of missingness allowed, and hence the contraction rate parameter, no longer depend on the incoherence of $V_{K}$, and can be of constant order.

Corollary 1 *Consider the* p‐*homogeneous MCAR setting. Fix* $U\in {\mathbb{R}}^{n\times K}$ *and* $V_{K}\in {𝕆}^{d\times K}$ *with* $\|V_{K}{\|}_{2\to \infty }\leq \mu {\left(K/d\right)}^{1/2}$, *and let* $Y:=UV_{K}^{⊤}$, *with* $c:=\sigma_{K}(Y)/\|Y{\|}_{F}$. *Suppose that* ${\hat{V}}_{K}^{\left(\text{in}\right)},{\hat{V}}_{K}^{\left(\text{out}\right)}\in {𝕆}^{d\times K}$, $O,\hat{O}\in {𝕆}^{K\times K}$ and $\Xi \in {\mathbb{R}}^{d\times K}$ are as in Proposition 3, let $C_{*}:=\|\Xi {\|}_{\text{op}}/\|\Xi {\|}_{2\to \infty }$, and, fixing δ ∈ (0,1), suppose that

$$\|\Xi {\|}_{\text{op}}\leq \min\{\frac{p{\left(1-p\right)}^{1/2}}{44\mu K^{3/2}(\sigma_{*}\vee 1)\log(24nK/\delta )},{\left(\frac{c}{8\overset{2}{\underset{*}{\sigma }}}\right)}^{1/2},\frac{c}{4\mu \sigma_{*}^{2}K}{\left(\frac{d}{\log K}\right)}^{1/2}\}.$$

*Suppose that* dp≥8log(3/δ). *Then with probability at least* 1−δ, *the output* ${\hat{V}}_{K}^{\left(\text{out}\right)}:=\text{refine}(K,{\hat{V}}_{K}^{\left(\text{in}\right)},\Omega ,Y_{\Omega })$ *of Algorithm* 1 *satisfies*

$$\|{\hat{V}}_{K}^{\left(\text{out}\right)}-V_{K}\hat{O}{\|}_{\text{op}}\leq \frac{125}{c}\{K^{1/2}{\left(1-p\right)}^{1/2}+\frac{\log^{1/2}(3/\delta )}{C_{*}}\}\|{\hat{V}}_{K}^{\left(\text{in}\right)}-V_{K}O{\|}_{\text{op}}.$$

To understand the conclusion of Corollary 1, it is instructive to consider the special case K=1. Here, c=1 and $C_{*}$ is the ratio of the $\ell_{2}$ and $\ell_{\infty }$ norms of the vector ${\hat{V}}_{1}^{\left(\text{in}\right)}-\text{sgn}(V_{1}^{⊤}{\hat{V}}_{1}^{\left(\mathrm{in}\right)})V_{1}$. When the entries of this vector are of comparable magnitude, $C_{*}$ is therefore of order $d^{1/2}$, so the contraction rate is of order ${\left(1-p\right)}^{1/2}+d^{-1/2}$.

### 3.3 Other missingness mechanisms

Another interesting aspect of our theory is that the guarantees provided in Theorem 3 are deterministic. Provided we start with a sufficiently good initialiser, Theorem 3 describes the way in which the performance of primePCA improves over iterations. An attraction of this approach is that it offers the potential to study the performance of primePCA under more general missingness mechanisms. For instance, one setting of considerable practical interest is the MAR model, which postulates that our data vector $y=(y_{1},\ldots ,y_{d})$ and observation indicator vector ω satisfy

(14) $$\mathbb{P}(\omega =\epsilon |y=a)=\mathbb{P}\left(\omega =\epsilon {|⋂}_{j:\epsilon_{j}=1}\{y_{j}=a_{j}\}\right),$$

for all $\epsilon ={\left(\epsilon_{1},\ldots ,\epsilon_{d}\right)}^{⊤}\in {\left\{0,1\right\}}^{d}$ and $a={\left(a_{1},\ldots ,a_{d}\right)}^{⊤}\in {\mathbb{R}}^{d}$. In other words, the probability of seeing a particular missingness pattern only depends on the data vector through components of this vector that are observed. Thus, if we want to understand the performance of primePCA under different missingness mechanisms, such as specific MAR (or even MNAR) models, all we require is an analogue of Theorem 4 on the performance of the initialiser in these new missingness settings. Such results, however, are likely to be rather problem‐specific in nature, and it can be that choosing an initialiser based on available information on the dependence between the observations and the missingness mechanism makes it easier to prove the desired performance guarantees.

We now provide an example to illustrate how such initialisers can be constructed and analysed. Consider an MAR setting where the missingness pattern depends on the data matrix only through a fully observed categorical variable. In this case, we can construct a variant of the OPW estimator, denoted ${\hat{V}}_{K}^{\text{OPWv}}$, by modifying the weighted sample covariance matrix in (8) to condition on the fully observed covariate, and then take the leading eigenvectors of an appropriate average of these conditional weighted sample covariance matrices. Specifically, suppose that our data consist of independent and identically distributed copies $\left(y_{1},\omega_{1}),\ldots ,(y_{n},\omega_{n}\right)$ of $\left(y,\omega )=(y_{0},y_{1},\ldots ,y_{d},\omega_{0},\omega_{1},\ldots ,\omega_{d}\right)$, where $\omega_{0}=1$, where $y_{0}$ is a categorical random variable taking values in {1,…,L} and where $\left(y_{1},\ldots ,y_{d})|y_{0}∼N_{d}(0,\sum_{y_{0}}\right)$ is independent of $\omega_{j}|y_{0}\;\overset{\text{iid}}{˜}\;\text{Bern}(p_{y_{0}})$ for all j∈[d]. Writing $y_{-0}:={\left(y_{1},\ldots ,y_{d}\right)}^{⊤}$, $\omega_{-0}:={\left(\omega_{1},\ldots ,\omega_{d}\right)}^{⊤}$ and ${\tilde{y}}_{-0}:=y_{-0}\circ \omega_{-0}$, we have that $\text{Cov}(y_{0},y_{-0})=0$, that is, Cov(y) is block diagonal. Thus, introducing the subscript i for our ith observation, as a starting point to construct ${\hat{V}}_{K}^{\text{OPWv}}$, it is natural to consider an oracle estimator of $\text{Cov}(y_{-0})$, given by

$$G:=\frac{1}{n}\overset{L}{\underset{\ell =1}{\sum }}\underset{i:y_{i0}=\ell }{\sum }{\tilde{y}}_{i,-0}{\tilde{y}}_{i,-0}^{⊤}\circ W_{\ell },$$

where $W_{\ell }:=p_{\ell }^{-2}\{1_{d}1_{d}^{⊤}-(1-p_{\ell })I_{d}\}$. Observe that we can write

$$G=\overset{L}{\underset{\ell =1}{\sum }}\frac{n_{\ell }}{n}G^{\left(\ell \right)},$$

where $n_{\ell }:=|\{i:y_{i0}=\ell \}|$ and where $G^{\left(\ell \right)}:=n_{\ell }^{-1}\sum_{i:y_{i0}=\ell }{\tilde{y}}_{i,-0}{\tilde{y}}_{i,-0}^{⊤}\circ W_{\ell }$ is the OPW estimator of $\sum_{\ell }$ based on the observations with $y_{i0}=\ell$. Hence, G is unbiased for $\text{Cov}(y_{-0})$, because

$$𝔼(G)=\overset{L}{\underset{\ell =1}{\sum }}𝔼\left(\frac{n_{\ell }}{n}𝔼(G^{\left(\ell \right)}|y_{10},\ldots ,y_{n0})\right)=\overset{L}{\underset{\ell =1}{\sum }}\frac{𝔼(n_{\ell })}{n}\sum_{\ell }=\text{Cov}(y_{-0}).$$

In practice, when $p_{\ell }$ is unknown, we can estimate it by

$${\hat{p}}_{\ell }:=\frac{1}{dn_{\ell }}\underset{i:y_{i0}=\ell }{\sum }\overset{d}{\underset{j=1}{\sum }}\omega_{ij},$$

and substitute this estimate into $W_{\ell }$ to obtain empirical estimators ${\tilde{G}}^{\left(\ell \right)}$ and $\tilde{G}$ of $G^{\left(\ell \right)}$ and G, respectively. Finally, ${\hat{V}}_{K}^{\text{OPWv}}$ can be obtained as the matrix of top K eigenvectors of the (d+1)×(d+1) matrix

$$\left(\begin{array}{cc} \frac{1}{n}\overset{n}{\underset{i=1}{\sum }}y_{i0}^{2}-{\left(\frac{1}{n}\overset{n}{\underset{i=1}{\sum }}y_{i0}\right)}^{2} & 0^{⊤}\\ 0 & \tilde{G} \end{array}\right).$$

To sketch the way to bound the sinΘ loss of such an initialiser, we can condition on $y_{10},\ldots ,y_{n0}$ and apply matrix Bernstein concentration inequalities similarly to those in the proof of Theorem 1 to show that ${\tilde{G}}^{\left(\ell \right)}$ is close to $\sum_{\ell }$ for each ℓ. Simple binomial concentration bounds then allow us to combine these to control $\|\tilde{G}-\text{Cov}(y_{-0}){\|}_{\text{op}}$, and then apply a variant the Davis–Kahan theorem to obtain a final result.

While different initialisers can be designed and analysed theoretically in specific missingness settings, as shown in the example above, our empirical experience, nevertheless, is that regardless of the missingness mechanism, primePCA is extremely robust to the choice of initialiser. This is evident from the discussion of the performance of primePCA in MAR and MNAR settings given in Section 4.4.

## 4 SIMULATION STUDIES

In this section, we assess the empirical performance of primePCA, as proposed in Algorithm 2, with initialiser ${\tilde{V}}_{K}$ from Section 3.1, and denote the output of this algorithm by ${\hat{V}}_{K}^{\text{prime}}$. In Sections 4.1, 4.2 and 4.3, we generate observations according to the model described in (1), (2) and (3) where the rows of the matrix U are independent $N_{d}(0,\sum_{u})$ random vectors, for some positive semi‐definite $\sum_{u}\in {\mathbb{R}}^{d\times d}$. We further generate the observation indicator matrix Ω, independently of U and Z, and investigate the following four missingness mechanisms that represent different levels of heterogeneity:

(H1) Homogeneous: $\mathbb{P}(\omega_{ij}=1)=0.05$ for all i∈[n],j∈[d];
(H2) Mildly heterogeneous: $\mathbb{P}(\omega_{ij}=1)=P_{i}Q_{j}$ for i∈[n],j∈[d], where $P_{1},\ldots ,P_{n}\overset{\text{iid}}{˜}U[0,0.2]$ and $Q_{1},\ldots ,Q_{d}\overset{\text{iid}}{˜}U[0.05,0.95]$ independently;
(H3) Highly heterogeneous columns: $\mathbb{P}(\omega_{ij}=1)=0.19$ for i∈[n] and all odd j∈[d] and $\mathbb{P}(\omega_{ij}=1)=0.01$ for i∈[n] and all even j∈[d].
(H4) Highly heterogeneous rows: $\mathbb{P}(\omega_{ij}=1)=0.18$ for j∈[d] and all odd i∈[n] and $\mathbb{P}(\omega_{ij}=1)=0.02$ for j∈[d] and all even i∈[n].

In Sections 4.1, 4.2 and 4.3 below, we investigate primePCA in noiseless, noisy and misspecified settings, respectively. Section 4.4 is devoted to MAR and MNAR settings. In all cases, the average statistical error was estimated from 100 Monte Carlo repetitions of the experiment. For comparison, we also studied the softImpute algorithm (Hastie et al., 2015; Mazumder et al., 2010), which is considered to be state‐of‐the‐art for matrix completion (Chi et al., 2018). This algorithm imputes the missing entries of Y by solving the following nuclear‐norm‐regularised optimisation problem:

$${\hat{y}}^{\text{soft}}:=\underset{X\in {\mathbb{R}}^{n\times d}}{\text{argmin}}\left\{\frac{1}{2}\|Y_{\Omega }-X_{\Omega }{\|}_{F}^{2}+\lambda \|X{\|}_{*}\right\},$$

where λ>0 is to be chosen by the practitioner. The softImpute estimator of $V_{K}$ is then given by the matrix of top K right singular vectors ${\hat{V}}_{K}^{\text{soft}}$ of ${\hat{Y}}^{\text{soft}}$. In practice, the optimisation is carried out by representing X as $AB^{⊤}$, and performing alternating projections to update $A\in {\mathbb{R}}^{n\times K}$ and $B\in {\mathbb{R}}^{d\times K}$ iteratively. The fact that the softImpute algorithm was originally intended for matrix completion means that it treats the left and right singular vectors symmetrically, whereas the primePCA algorithm, which has the advantage of a clear geometric interpretation as exemplified in Figure 1, focuses on the target of inference in PCA, namely the leading right singular vectors.

**FIGURE 2:**
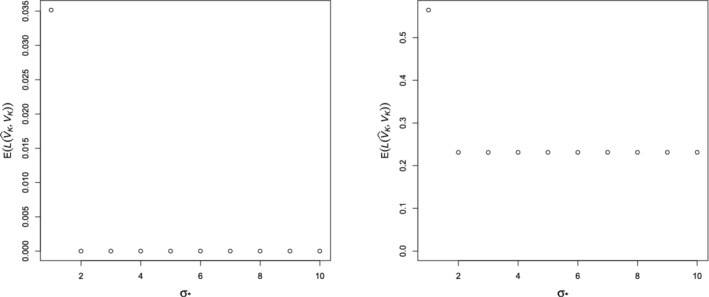
Estimates of $𝔼L({\hat{V}}_{K}^{\text{prime}},V_{K})$ for various choices of $\sigma_{*}$ under (H1) in the noiseless setting of Section 4.1 (left) and (H2) in the noisy setting of Section 4.2 with ν=20 (right)

Figure 2 presents Monte Carlo estimates of $𝔼L({\hat{V}}_{K}^{\text{prime}},V_{K})$ for different choices of $\sigma_{*}$ in two different settings. The first uses the noiseless set‐up of Section 4.1, together with missingness mechanism (H1); the second uses the noisy setting of Section 4.2 with parameter ν=20 and missingness mechanism (H2). We see that the error barely changes when $\sigma_{*}$ varies within [2,10]; very similar plots were obtained for different data generation and missingness mechanisms, though we omit these for brevity. For definiteness, we therefore fixed $\sigma_{*}=3$ throughout our simulation study.

### 4.1 Noiseless case

In the noiseless setting, we let Z=0, and also fix n=2000, d=500, K=2 and $\sum_{u}=100I_{2}$. We set

$$V_{K}=\sqrt{\frac{1}{500}}\left(\begin{array}{cc} 1_{250} & 1_{250}\\ 1_{250} & -1_{250} \end{array}\right)\in {\mathbb{R}}^{500\times 2}.$$

**FIGURE 3:**
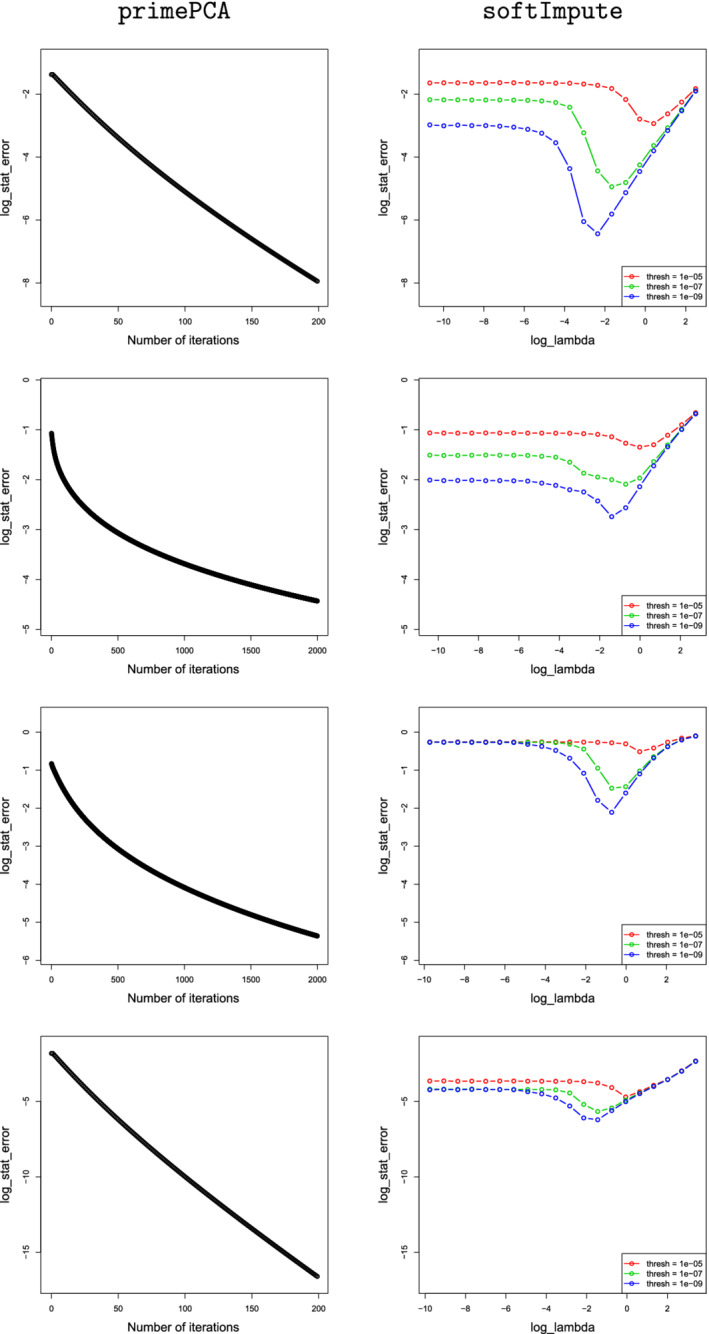
Logarithms of the average Frobenius norm sinΘ error of primePCA and softImpute under various heterogeneity levels of missingness in absence of noise. The four rows of plots above, from the top to bottom, correspond to (H1), (H2), (H3) and (H4). [Colour figure can be viewed at wileyonlinelibrary.com]

In Figure 3, we present the (natural) logarithm of the estimated average loss of primePCA and softImpute under (H1), (H2), (H3) and (H4). We set the range of y‐axis to be the same for each method to facilitate straightforward comparison. We see that the statistical error of primePCA decreases geometrically as the number of iterations increases, which confirms the conclusion of Theorem 3 in this noiseless setting. Moreover, after a moderate number of iterations, its performance is a substantial improvement on that of the softImpute algorithm, even if this latter algorithm is given access to an oracle choice of the regularisation parameter λ. The high statistical error of softImpute in these settings can be partly explained by the default value of the tuning parameter thresh in the softImpute package in R, namely $10^{-5}$, which corresponds to the red curve in the right‐hand panels of Figure 3. By reducing the values of thresh to $10^{-7}$ and $10^{-9}$, corresponding to the green and blue curves in Figure 3, respectively, we were able to improve the performance of softImpute to some extent, though the statistical error is sensitive to the choice of the regularisation parameter λ. Moreover, even with the optimal choice of λ, it is not competitive with primePCA. Finally, we mention that for the 2000 iterations of setting (H2), primePCA took on average just under 10 min per repetition to compute, whereas the solution path of softImpute with $\text{thresh}=10^{-9}$ took around 36 min per repetition.

### 4.2 Noisy case

Here, we generate the rows of Z as independent $N_{d}(0,I_{d})$ random vectors, independent of all other data. We maintain the same choices of n, d, K and $V_{K}$ as in Section 4.1, set $\sum_{u}=\nu^{2}I_{2}$ and vary ν>0 to achieve different SNRs. In particular, defining $\text{SNR}:=\text{Tr}\text{Cov}(x_{1})/\text{Tr}\text{Cov}(z_{1})$, the choices ν=10,20,40,60 correspond to the very low, low, medium and high SNR=0.4,1.6,6.4,14.4, respectively. For an additional comparison, we consider a variant of the softImpute algorithm called hardImpute (Mazumder et al., 2010), which retains only a fixed number of top singular values in each iteration of matrix imputation; this can be achieved by setting the argument λ in the softImpute function to be 0.

To avoid confounding our study of the statistical performance of the softImpute algorithm with the choice of regularisation parameter λ, we gave the softImpute algorithm a particularly strong form of oracle choice of λ, namely where λ was chosen for each individual repetition of the experiment, so as to minimise the loss function. Naturally, such a choice is not available to the practitioner. Moreover, in order to ensure the range of λ was wide enough to include the best softImpute solution, we set the argument rank.max in that algorithm to be 20.

In Table 1, we report the statistical error of primePCA after 2000 iterations of refinement, together with the corresponding statistical errors of our initial estimator primePCA_init and those of softImpute(oracle) and hardImpute. Remarkably, primePCA exhibits stronger performance than these other methods across each of the SNR regimes and different missingness mechanisms. We also remark that hardImpute is inaccurate and unstable, because it might converge to a local optimum that is far from the truth.

**TABLE 1:** Average losses (with SEs in brackets) under (H1), (H2), (H3) and (H4)

		ν=10	ν=20	ν=40	ν=60
(H1)	hardImpute	$0.891_{\left(0.005\right)}$	$0.444_{\left(0.001\right)}$	$0.251_{\left(0.001\right)}$	$0.186_{\left(0.0005\right)}$
	softImpute(oracle)	$0.377_{\left(0.0009\right)}$	$0.186_{\left(0.0004\right)}$	$0.095_{\left(0.0002\right)}$	$0.064_{\left(0.0002\right)}$
	primePCA_init	$0.449_{\left(0.001\right)}$	$0.306_{\left(0.001\right)}$	$0.266_{\left(0.001\right)}$	$0.259_{\left(0.001\right)}$
	primePCA	$0.368_{\left(0.001\right)}$	$0.171_{\left(0.0004\right)}$	$0.084_{\left(0.0002\right)}$	$0.056_{\left(0.0001\right)}$
(H2)	hardImpute	$0.920_{\left(0.006\right)}$	$0.473_{\left(0.001\right)}$	$0.291_{\left(0.001\right)}$	$0.236_{\left(0.001\right)}$
	softImpute(oracle)	$0.519_{\left(0.001\right)}$	$0.308_{\left(0.001\right)}$	$0.185_{\left(0.001\right)}$	$0.141_{\left(0.001\right)}$
	primePCA_init	$0.549_{\left(0.002\right)}$	$0.399_{\left(0.002\right)}$	$0.357_{\left(0.001\right)}$	$0.349_{\left(0.001\right)}$
	primePCA	$0.475_{\left(0.002\right)}$	$0.232_{\left(0.001\right)}$	$0.115_{\left(0.001\right)}$	$0.077_{\left(0.0005\right)}$
(H3)	hardImpute	$0.792_{\left(0.003\right)}$	$0.479_{\left(0.001\right)}$	$0.385_{\left(0.001\right)}$	$0.427_{\left(0.001\right)}$
	softImpute(oracle)	$0.622_{\left(0.002\right)}$	$0.374_{\left(0.001\right)}$	$0.222_{\left(0.001\right)}$	$0.170_{\left(0.001\right)}$
	primePCA_init	$0.624_{\left(0.002\right)}$	$0.486_{\left(0.001\right)}$	$0.449_{\left(0.001\right)}$	$0.442_{\left(0.001\right)}$
	primePCA	$0.581_{\left(0.002\right)}$	$0.290_{\left(0.001\right)}$	$0.145_{\left(0.001\right)}$	$0.097_{\left(0.0004\right)}$
(H4)	hardImpute	$0.368_{\left(0.001\right)}$	$0.174_{\left(0.0005\right)}$	$0.089_{\left(0.0003\right)}$	$0.062_{\left(0.0003\right)}$
	softImpute(oracle)	$0.243_{\left(0.0006\right)}$	$0.121_{\left(0.0002\right)}$	$0.062_{\left(0.0001\right)}$	$0.042_{\left(0.0001\right)}$
	primePCA_init	$0.290_{\left(0.0007\right)}$	$0.203_{\left(0.001\right)}$	$0.175_{\left(0.0005\right)}$	$0.169_{\left(0.0004\right)}$
	primePCA	$0.238_{\left(0.0006\right)}$	$0.116_{\left(0.0003\right)}$	$0.058_{\left(0.0002\right)}$	$0.038_{\left(0.0001\right)}$

### 4.3 Near low‐rank case

In this sub‐section, we set n=2000, d=500, K=10, $\sum_{u}=\text{diag}(2^{10},2^{9},\ldots ,2)$, and fixed $V_{K}$ once for all experiments to be the top K eigenvectors of one realisation^2^ of the sample covariance matrix of n independent $N_{d}(0,I_{d})$ random vectors. Here $d^{1/2}\|V_{K}{\|}_{2\to \infty }<1.72$, and we again generated the rows of Z as independent $N_{d}(0,I_{d})$ random vectors. Table 2 reports the average loss of estimating the top $\hat{K}$ eigenvectors of $\sum_{y}$, where $\hat{K}$ varies from 1 to 5. Interestingly, even in this misspecified setting, primePCA is competitive with the oracle version of softImpute.

**TABLE 2:** Average losses (with SEs in brackets) in the setting of Section 4.3 under (H1), (H2), (H3) and (H4)

		$\hat{K}=1$	$\hat{K}=2$	$\hat{K}=3$	$\hat{K}=4$	$\hat{K}=5$
(H1)	hardImpute	$0.308_{\left(0.002\right)}$	$0.507_{\left(0.002\right)}$	$0.764_{\left(0.004\right)}$	$1.199_{\left(0.006\right)}$	$1.524_{\left(0.004\right)}$
	softImpute(oracle)	$0.107_{\left(0.001\right)}$	$0.182_{\left(0.001\right)}$	$0.275_{\left(0.001\right)}$	$0.401_{\left(0.001\right)}$	$0.596_{\left(0.001\right)}$
	primePCA_init	$0.203_{\left(0.001\right)}$	$0.345_{\left(0.001\right)}$	$0.554_{\left(0.003\right)}$	$1.074_{\left(0.007\right)}$	$1.427_{\left(0.006\right)}$
	primePCA	$0.141_{\left(0.001\right)}$	$0.200_{\left(0.001\right)}$	$0.269_{\left(0.001\right)}$	$0.374_{\left(0.001\right)}$	$0.580_{\left(0.001\right)}$
(H2)	hardImpute	$0.298_{\left(0.002\right)}$	$0.466_{\left(0.002\right)}$	$0.696_{\left(0.003\right)}$	$1.124_{\left(0.006\right)}$	$1.452_{\left(0.004\right)}$
	softImpute(oracle)	$0.188_{\left(0.001\right)}$	$0.283_{\left(0.001\right)}$	$0.410_{\left(0.001\right)}$	$0.562_{\left(0.001\right)}$	$0.751_{\left(0.001\right)}$
	primePCA_init	$0.285_{\left(0.001\right)}$	$0.443_{\left(0.004\right)}$	$0.757_{\left(0.013\right)}$	$1.201_{\left(0.004\right)}$	$1.533_{\left(0.003\right)}$
	primePCA	$0.190_{\left(0.002\right)}$	$0.267_{\left(0.002\right)}$	$0.368_{\left(0.003\right)}$	$0.543_{\left(0.008\right)}$	$0.797_{\left(0.009\right)}$
(H3)	hardImpute	$0.302_{\left(0.001\right)}$	$0.482_{\left(0.002\right)}$	$0.695_{\left(0.002\right)}$	$1.004_{\left(0.006\right)}$	$1.373_{\left(0.004\right)}$
	softImpute(oracle)	$0.206_{\left(0.001\right)}$	$0.338_{\left(0.001\right)}$	$0.492_{\left(0.001\right)}$	$0.664_{\left(0.002\right)}$	$0.878_{\left(0.002\right)}$
	primePCA_init	$0.341_{\left(0.001\right)}$	$0.528_{\left(0.019\right)}$	$1.097_{\left(0.008\right)}$	$1.306_{\left(0.008\right)}$	$1.597_{\left(0.004\right)}$
	primePCA	$0.222_{\left(0.001\right)}$	$0.330_{\left(0.002\right)}$	$0.452_{\left(0.003\right)}$	$0.641_{\left(0.008\right)}$	$0.919_{\left(0.007\right)}$
(H4)	hardImpute	$0.090_{\left(0.001\right)}$	$0.148_{\left(0.001\right)}$	$0.226_{\left(0.001\right)}$	$0.346_{0.002}$	$0.589_{\left(0.007\right)}$
	softImpute(oracle)	$0.071_{\left(0.001\right)}$	$0.112_{\left(0.001\right)}$	$0.164_{\left(0.001\right)}$	$0.233_{\left(0.001\right)}$	$0.332_{\left(0.001\right)}$
	primePCA_init	$0.139_{\left(0.001\right)}$	$0.220_{\left(0.001\right)}$	$0.325_{\left(0.001\right)}$	$0.475_{\left(0.002\right)}$	$0.805_{\left(0.012\right)}$
	primePCA	$0.098_{\left(0.001\right)}$	$0.135_{\left(0.001\right)}$	$0.176_{\left(0.001\right)}$	$0.236_{\left(0.001\right)}$	$0.328_{\left(0.001\right)}$

### 4.4 Other missingness mechanisms

Finally in this section, we investigate the performance of primePCA, as well as other alternative algorithms, in settings where the MCAR hypothesis is not satisfied. We consider two simulation frameworks to explore both MAR (see (14)) and MNAR mechanisms. In the first, we assume that missingness depends on the data matrix Y only through a fully observed covariate, as in the example in Section 3.3. Specifically, for some α≥0, for K=2, and for two matrices^3^ $V_{+},V_{-}\in {𝕆}^{d\times 2}$, the pair $\left(y_{1},\omega_{1})=(y_{10},y_{11}\ldots ,y_{1d},\omega_{10},\omega_{11},\ldots ,\omega_{1d}\right)$ is generated as follows:

(15) $$\begin{array}{rl} \omega_{10} & =1,\;y_{10}∼\text{Unif}\{-1,1\},\\ (y_{11},\ldots ,y_{1d}),\omega_{11},\ldots ,\omega_{1d} & \;\text{are conditionally independent given}\;y_{10},\\ {\left(y_{11},\ldots ,y_{1d}\right)}^{⊤}|y_{10} & ∼\left\{\begin{array}{cc} N_{d}(0,V_{+}\text{diag}(40,10)V_{+}^{⊤}+I_{d})\; & \text{if}\;y_{10}=1\\ N_{d}(0,V_{-}\text{diag}(40,10)V_{-}^{⊤}+I_{d})\; & \text{if}\;y_{10}=-1, \end{array}\right.\\ \mathbb{P}(\omega_{1j}=1|y_{10}) & ={\left\{1+\exp(\frac{j}{d}+y_{10}\alpha )\right\}}^{-1},\;\text{for}\;j\in [d]. \end{array}$$

The other rows of (Y,Ω) are taken to be as independent copies of $\left(y_{1},\omega_{1}\right)$. Thus, when α=0, the matrices Y and Ω are independent, and we are in an MCAR setting; when α≠0, the data are MAR but not MCAR, and α measures the extent of departure from the MCAR setting. The covariance matrix of $y_{1}$ is

$$\sum_{y}=\left(\begin{array}{cc} 1 & 0^{⊤}\\ 0 & \frac{1}{2}V_{+}\text{diag}(40,10)V_{+}^{⊤}+\frac{1}{2}V_{-}\text{diag}(40,10)V_{-}^{⊤}+I_{d} \end{array}\right)\in {\mathbb{R}}^{\left(d+1)\times (d+1\right)}.$$

In this example, we can construct a variant of the OPW estimator, which we call the OPWv estimator, by exploiting the fact that, conditional on the fully observed first column of Y, the data are MCAR. To do this, let

$${\hat{\sum }}^{\text{OPWv}}:=\left(\begin{array}{cc} 1 & 0^{⊤}\\ 0 & \frac{1}{2}{\tilde{G}}_{+}+\frac{1}{2}{\tilde{G}}_{-} \end{array}\right),$$

where ${\tilde{G}}_{+}$ and ${\tilde{G}}_{-}$ are the weighted sample covariance matrices computed as in (8), based on data ${\left(y_{ij},\omega_{ij}\right)}_{i:y_{i0}=1,j\in [d]}$ and ${\left(y_{ij},\omega_{ij}\right)}_{i:y_{i0}=-1,j\in [d]}$, respectively. The OPWv estimator is the matrix of the first two eigenvectors of ${\hat{\sum }}^{\text{OPWv}}$. Both the OPW and OPWv estimators are plausible initialisers for the primePCA algorithm.

In low‐dimensional settings, likelihood‐based approaches, often implemented via an EM algorithm, are popular for handling MAR data (14) (Rubin, 1976). In Table 3, we compare the performance of primePCA in this setting with that of an EM algorithm derived from the suggestion in Little and Rubin (2019), section 11.3, and considered both the OPW and OPWv estimators as initialisers. We set n=500, d∈{25,50}, α∈{0.1,0.5} and took $\hat{K}=2$ for both the primePCA and the EM algorithms. From the table, we see that the OPWv estimator is able to exploit the group structure of the data to improve upon the OPW estimator, especially for the larger value of α. It is reassuring to find that the performance of primePCA is completely unaffected by the choice of initialiser, and, remarkably, it outperforms the OPWv estimator, even though the latter has access to additional model structure information. The worse root mean squared error of the EM algorithm is mainly due its numerical instability when performing Schur complement computations^4^.

**TABLE 3:** Root mean squared errors of the sinΘ loss function (with SEs in brackets) over 100 repetitions from the data‐generating mechanism in (15) for observed‐proportion weighted (OPW) estimator and its class‐weighted variant (OPWv), expectation‐mMaximisation (EM) and primePCA with both the OPW or OPWv initialisers

d	α	OPW	OPWv	EM	EMv	primePCA	primePCAv
25	0.1	$0.266_{\left(0.005\right)}$	$0.247_{\left(0.004\right)}$	$0.414_{\left(0.045\right)}$	$0.464_{\left(0.053\right)}$	$0.206_{\left(0.004\right)}$	$0.206_{\left(0.004\right)}$
25	0.5	$0.346_{\left(0.009\right)}$	$0.248_{\left(0.005\right)}$	$0.445_{\left(0.047\right)}$	$0.378_{\left(0.056\right)}$	$0.248_{\left(0.014\right)}$	$0.248_{\left(0.008\right)}$
50	0.1	$0.287_{\left(0.003\right)}$	$0.265_{\left(0.003\right)}$	$0.350_{\left(0.032\right)}$	$0.346_{\left(0.032\right)}$	$0.220_{\left(0.002\right)}$	$0.220_{\left(0.002\right)}$
50	0.5	$0.591_{\left(0.025\right)}$	$0.290_{\left(0.003\right)}$	$0.588_{\left(0.033\right)}$	$0.369_{\left(0.03\right)}$	$0.255_{\left(0.005\right)}$	$0.255_{\left(0.005\right)}$

The second simulation framework is as follows. Let $\sum :={\left(\min\{j,k\}\right)}_{j,k\in [d]}\in {\mathbb{R}}^{d\times d}$ and let $\xi ={\left(\xi_{ij}\right)}_{i\in [n],j\in [d]}$ be a latent Bernoulli thinning matrix. The data matrix $Y={\left(y_{ij}\right)}_{i\in [n],j\in [d]}$ and revelation matrix $\Omega ={\left(\omega_{ij}\right)}_{i\in [n],j\in [d]}$ are generated in such a way that Y and ξ are independent,

(16) $$\begin{array}{rl} {\left(y_{i1},\ldots ,y_{id}\right)}^{⊤}\; & \overset{\text{iid}}{˜}\;N_{d}(0,\sum ),\;\text{for}\;i\in [n],\\ \xi_{ij}\; & \overset{\text{iid}}{˜}\;\text{Bern}(p),\\ \omega_{ij} & =\xi_{ij}1_{\left\{\underset{1\leq t<j}{\max}|y_{it}|<\tau \right\}},\;\text{for some}\;\tau >0, \end{array}$$

(where the maximum of the empty set is −∞ by convention). As usual, we observe (Y∘Ω,Ω). In other words, viewing each $\left(y_{i1},\ldots ,y_{id}\right)$ as a d‐step standard Gaussian random walk, we observe Bernoulli‐thinned paths of the process up to (and including) the hitting time of the threshold ±τ. We note that the observations satisfy the MAR hypothesis if and only if p=1, and as p decreases from 1, the mechanism becomes increasingly distant from MAR, as we become increasingly likely to fail to observe the threshold hitting time. We take K=1.

In Table 4, we compare the performance of primePCA with that of the EM algorithm, and in both cases, we can initialise with either the OPW estimator or a mean‐imputation estimator, obtained by imputing all missing entries by their respective population column means. We set n=500, d=100, $\tau =d^{1/2}$, took $\hat{K}=1$ for both primePCA and the EM algorithm, and took p∈{0.25,0.5,0.75,1}. From the table, we see that primePCA outperforms the EM algorithm except in the MAR case where p=1, which is tailor‐made for the likelihood‐based EM approach. In fact, primePCA is highly robust statistically and stable computationally, performing well consistently across different missingness settings and initialisers. On the other hand, the EM algorithm exhibits a much heavier dependence on the initialiser: its statistical performance suffers when initialised with the poorer mean‐imputation estimator and runs into numerical instability issues when initialised with the OPW estimator in the MNAR settings. We found that these instability issues are exacerbated in higher dimensions, and moreover, that the EM algorithm quickly becomes computationally infeasible^5^. This explains why we did not run the EM algorithm on the larger‐scale problems in Sections 4.1, 4.2 and 4.3, as well as the real data example in Section 5 below.

**TABLE 4:** Root mean squared errors of the sinΘ loss function (with SEs in brackets) over 100 repetitions from the data‐generating mechanism in (16) for mean‐imputation estimator (MI), observed‐proportion weighted (OPW) estimator, Expectation–Maximisation (EM) and primePCA with both MI and OPW initialisers (distinguished by subscripts in table header)

p	MI	OPW	EM${\;}_{\text{MI}}$	EM${\;}_{\text{OPW}}$	primePCA${\;}_{\text{MI}}$	primePCA${\;}_{\text{OPW}}$
1	$0.548_{\left(0.004\right)}$	$0.282_{\left(0.004\right)}$	$0.086_{\left(0.003\right)}$	$0.056_{\left(0.002\right)}$	$0.096_{\left(0.002\right)}$	$0.096_{\left(0.002\right)}$
0.75	$0.551_{\left(0.004\right)}$	$0.285_{\left(0.004\right)}$	$0.117_{\left(0.004\right)}$	$0.353_{\left(0.041\right)}$	$0.097_{\left(0.002\right)}$	$0.097_{\left(0.002\right)}$
0.5	$0.557_{\left(0.005\right)}$	$0.29_{\left(0.004\right)}$	$0.186_{\left(0.025\right)}$	$0.944_{\left(0.013\right)}$	$0.1_{\left(0.002\right)}$	$0.1_{\left(0.002\right)}$
0.25	$0.575_{\left(0.005\right)}$	$0.309_{\left(0.005\right)}$	$0.228_{\left(0.005\right)}$	$0.989_{\left(0.001\right)}$	$0.112_{\left(0.002\right)}$	$0.123_{\left(0.006\right)}$

## 5 REAL DATA ANALYSIS: MILLION SONG DATASET

We apply primePCA to a subset of the Million Song Dataset^6^ to analyse music preferences. The original data can be expressed as a matrix with 110,000 users (rows) and 163,206 songs (columns), with entries representing the number of times a song was played by a particular user. The proportion of non‐missing entries in the matrix is 0.008%. Since the matrix is very sparse, and since most songs have very few listeners, we enhance the SNR by restricting our attention to songs that have at least 100 listeners (1777 songs in total). This improves the proportion of non‐missing entries to 0.23%. Further summary information about the filtered data is provided below:

**Table:** 

(a) Quantiles of non‐missing matrix entry values:										
0%	10%	20%	30%	40%	50%	60%	70%	80%	90%	100%
1	1	1	1	1	1	2	3	5	8	500
90%	91%	92%	93%	94%	95%	96%	97%	98%	99%	100%
8	9	9	10	11	13	15	18	23	33	500
**(b) Quantiles of the number of listeners for each song:**										
0%	10%	20%	30%	40%	50%	60%	70%	80%	90%	100%
100	108	117	126	139	154	178	214	272.8	455.6	5043
**(c) Quantiles of the total play counts of each user:**										
0%	10%	20%	30%	40%	50%	60%	70%	80%	90%	100%
0	0	1	3	4	6	9	14	21	38	1114
90%	91%	92%	93%	94%	95%	96%	97%	98%	99%	100%
38	41	44	48	54	60	68	79	97	132	1114

We mention here a respect in which the data set does not conform exactly to the framework studied in the paper, namely that we treat zero entries as missing data (this is very common for analyses of user‐preference data sets). In practice, while it may indeed be the case that a zero play count for song j by user i provides no indication of their level of preference for that song, it may also be the case that it reflects a dislike of that song. To address this issue, following our main analysis we will present a study of the robustness of our conclusions to different levels of true zeros in the data.

From point (a) above, we see that the distribution of play counts has an extremely heavy tail, and in particular the sample variances of the counts will be highly heterogeneous across songs. To guard against excessive influence from the outliers, we discretise the play counts into five interest levels as follows:

**Table:** 

Play count	1	2–3	4–6	7–10	≥11
Level of interest	1	2	3	4	5

**FIGURE 4:**
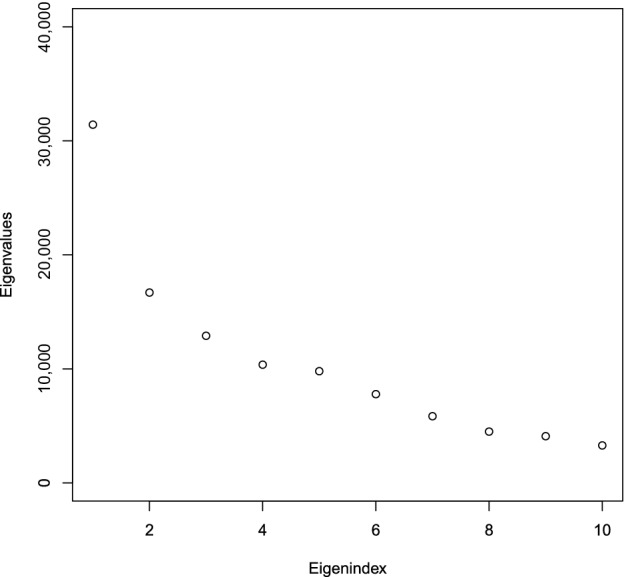
Leading eigenvalues of ${\hat{\sum }}_{y}$

We are now in a position to analyse the data using primePCA, noting that one of the attractions of estimating the principal eigenspaces in this setting (as opposed to matrix completion, for instance), is that it becomes straightforward to make recommendations to new users, instead of having to run the algorithm again from scratch. For i=1,…,n=110,000 and j=1,…,d=1,777, let $y_{ij}\in \{1,\ldots ,5\}$ denote the level of interest of user i in song j, let $\hat{K}=10$ and let $ℐ=\{i:\|\omega_{i}{\|}_{1}>\hat{K}\}$. Our initial goal is to assess the top $\hat{K}$ eigenvalues of $\sum_{y}$ to see if there is low‐rank signal in $Y=(y_{ij})$. To this end, we first apply Algorithm 2 to obtain ${\hat{V}}_{\hat{K}}^{\text{prime}}$; next, for each i∈ℐ, we run Steps 2–5 of Algorithm 1 to obtain the estimated principal score ${\hat{u}}_{i}$, so that we can approximate $y_{i}$ by ${\hat{y}}_{i}={\hat{V}}_{\hat{K}}^{\text{prime}}{\hat{u}}_{i}$. This allows us to estimate $\sum_{y}$ by ${\hat{\sum }}_{y}=n^{-1}\sum_{i\in ℐ}{\hat{y}}_{i}{\hat{y}}_{i}^{⊤}$. Figure 4 displays the top $\hat{K}$ eigenvalues of ${\hat{\sum }}_{y}$, which exhibit a fairly rapid decay, thereby providing evidence for the existence of low‐rank signal in Y.

**FIGURE 5:**
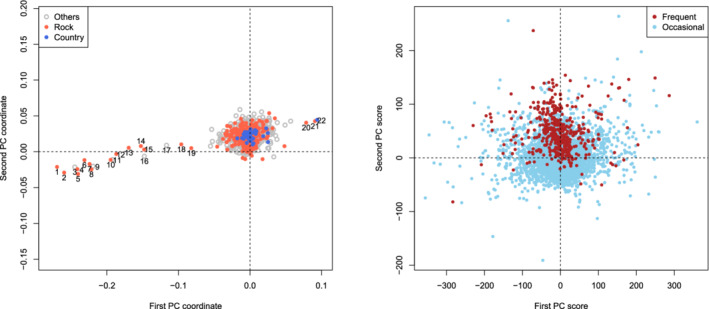
Plots of the first two principal components ${\hat{V}}_{2}^{\text{prime}}$ (left) and the associated scores ${\left\{{\hat{u}}_{i}\right\}}_{i=1}^{n}$ (right) [Colour figure can be viewed at wileyonlinelibrary.com]

In the left panel of Figure 5, we present the estimate ${\hat{V}}_{2}^{\text{prime}}$ of the top two eigenvectors of the covariance matrix $\sum_{y}$, with colours indicating the genre of the song. The outliers in the x‐axis of this plot are particularly interesting: they reveal songs that polarise opinion among users (see Table 5) and that best capture variation in individuals' preferences for types of music measured by the first principal component. It is notable that Rock songs are overrepresented among the outliers (see Table 6), relative to, say, Country songs. Users who express a preference for particular songs are also more likely to enjoy songs that are nearby in the plot. Such information is therefore potentially commercially valuable, both as an efficient means of gauging users' preferences, and for providing recommendations.

**TABLE 5:** Titles, artists and genres of the 22 outlier songs in Figure 5

ID	Title	Artist	Genre
1	Your Hand In Mine	Explosions In The Sky	Rock
2	All These Things That I've Done	The Killers	Rock
3	Lady Marmalade	Christina Aguilera / Lil' Kim/	Pop
		Mya / Pink	
4	Here It Goes Again	Ok Go	Rock
5	I Hate Pretending (Album Version)	Secret Machines	Rock
6	No Rain	Blind Melon	Rock
7	Comatose (Comes Alive Version)	Skillet	Rock
8	Life In Technicolor	Coldplay	Rock
9	New Soul	Yael Naïm	Pop
10	Blurry	Puddle Of Mudd	Rock
11	Give It Back	Polly Paulusma	Pop
12	Walking On The Moon	The Police	Rock
13	Face Down (Album Version)	The Red Jumpsuit Apparatus	Rock
14	Savior	Rise Against	Rock
15	Swing Swing	The All‐American Rejects	Rock
16	Without Me	Eminem	Rap
17	Almaz	Randy Crawford	Pop
18	Hotel California	Eagles	Rock
19	Hey There Delilah	Plain White T's	Rock
20	Revelry	Kings Of Leon	Rock
21	Undo	Björk	Rock
22	You're The One	Dwight Yoakam	Country

**TABLE 6:** Genre distribution of the outliers (songs whose corresponding coordinate in the estimated leading principal component is of magnitude larger than 0.07)

	Rock	Pop	Electronic	Rap	Country	RnB	Latin	Others
Population (Total =1,777)	48.92%	18.53%	9.12%	7.15%	4.33%	2.35%	2.26%	7.34%
Outliers (Total =22)	72.73%	18.18%	0%	4.54%	4.54%	0%	0%	0%

The right panel of Figure 5 presents the principal scores ${\left\{{\hat{u}}_{i}\right\}}_{i=1}^{n}$ of the users, with frequent users (whose total song plays are in the top 10% of all users) in red and occasional users in blue. This plot reveals, for instance, that the second principal component is well aligned with general interest in the website. Returning to the left plot, we can now interpret a positive y‐coordinate for a particular song (which is the case for the large majority of songs) as being associated with an overall interest in the music provided by the site.

As discussed above, it may be the case that some of the entries that we have treated as missing in fact represent a user's aversion to a particular song. We therefore studied the robustness of our conclusions by replacing some of the missing entries with an interest level of 1 (i.e. the lowest level available). More precisely, for some α∈{0.05,0.1,0.2}, and independently for each user i∈[n], we generated $R_{i}∼\text{Poisson}(\alpha \|\omega_{i}{\|}_{1})$, and assigned an interest level of 1 to $R_{i}$ uniformly random chosen songs that this user had not previously heard through the site. We then ran primePCA on this imputed dataset, obtaining estimators ${\hat{v}}_{1}^{\prime }$ and ${\hat{v}}_{2}^{\prime }$ of the two leading principal components. Denoting the original primePCA estimators for the two columns of $V_{2}$ by ${\hat{v}}_{1}$ and ${\hat{v}}_{2}$, respectively, Table 7 reports the average of the inner product $\left\langle {\hat{v}}_{j},{\hat{v}}_{k}^{\prime }\right\rangle$, where j,k∈{1,2}, based on 100 independent Monte Carlo experiments. Bearing in mind that the average absolute inner product between two independent random vectors chosen uniformly on ${𝒮}^{1776}$ is around 0.020, this table is reassuring that the conclusions are robust to the treatment of missing entries.

**TABLE 7:** Robustness assessment: average inner products (over 100 repetitions) between top two eigenvectors obtained by running primePCA on the original data and with some of the missing entries imputed with an interest level of 1

α	$\left\langle {\hat{v}}_{1},{\hat{v}}_{1}^{\prime }\right\rangle$	$\left\langle {\hat{v}}_{1},{\hat{v}}_{2}^{\prime }\right\rangle$	$\left\langle {\hat{v}}_{2},{\hat{v}}_{1}^{\prime }\right\rangle$	$\left\langle {\hat{v}}_{2},{\hat{v}}_{2}^{\prime }\right\rangle$
0.05	$0.816_{\left(0.018\right)}$	$-0.042_{\left(0.007\right)}$	$-0.012_{\left(0.007\right)}$	$0.910_{\left(0.002\right)}$
0.1	$0.756_{\left(0.018\right)}$	$-0.027_{\left(0.007\right)}$	$-0.070_{\left(0.008\right)}$	$0.893_{\left(0.002\right)}$
0.2	$0.546_{\left(0.025\right)}$	$-0.067_{\left(0.010\right)}$	$-0.085_{\left(0.010\right)}$	$0.859_{\left(0.002\right)}$

*Note*: SEs are given in brackets.

## 6 DISCUSSION

Heterogeneous missingness is ubiquitous in contemporary, large‐scale data sets, yet we currently understand very little about how existing procedures perform or should be adapted to cope with the challenges this presents. Here we attempt to extract the lessons learned from this study of high‐dimensional PCA, in order to see how related ideas may be relevant in other statistical problems where one wishes to recover low‐dimensional structure with data corrupted in a heterogeneous manner.

A key insight, as gleaned from Section 2.2, is that the way in which the heterogeneity interacts with the underlying structure of interest is crucial. In the worst case, the missingness may be constructed to conceal precisely the structure one seeks to uncover, thereby rendering the problem infeasible by any method. The only hope, then, in terms of providing theoretical guarantees, is to rule out such an adversarial interaction. This was achieved via our incoherence condition in Section 3, and we look forward to seeing how the relevant interactions between structure and heterogeneity can be controlled in other statistical problems such as those mentioned in the introduction. For instance, in sparse linear regression, one would anticipate that missingness of covariates with strong signal would be much more harmful than corresponding missingness for noise variables.

Our study also contributes to the broader understanding of the uses and limitations of spectral methods for estimating hidden low‐dimensional structures in high‐dimensional problems. We have seen that the OPW estimator is both methodologically simple and, in the homogeneous missingness setting, achieves near‐minimax optimality when the noise level is of constant order. Similar results have been obtained for spectral clustering for network community detection in stochastic block models (Rohe et al., 2011) and in low‐rank‐plus‐sparse matrix estimation problems (Fan et al., 2013). On the other hand, the OPW estimator fails to provide exact recovery of the principal components in the noiseless setting. In these other aforementioned problems, it has also been observed that refinement of an initial spectral estimator can enhance performance, particularly in high SNR regimes (Gao et al., 2016; Zhang et al., 2018), as we were able to show for our primePCA algorithm. This suggests that such a refinement has the potential to confer a sharper dependence of the statistical error rate on the SNR compared with a vanilla spectral algorithm, and understanding this phenomenon in greater detail provides another interesting avenue for future research.

## Supporting information




**Data S1**: Supporting information

## Footnotes

1 When K=1, we have that $L({\hat{V}}_{1},V_{1})$ is the sine of the acute angle between ${\hat{V}}_{1}$ and $V_{1}$. More generally, $L^{2}({\hat{V}}_{K},V_{K})$ is the sum of the squares of the sines of the principal angles between the subspaces spanned by ${\hat{V}}_{K}$ and $V_{K}$.

2 In R, we set the random seed to be 2019 before generating $V_{K}$.

3 To be completely precise, in our simulations, $V_{+}$ and $V_{-}$ were generated independently (and independently of all other randomness) and were drawn from Haar measure on ${𝕆}^{d\times 2}$; however, these matrices were then fixed for every replication, so it is convenient to regard them as deterministic for the purposes of this description.

4 In fact, to try to improve the numerical stability of the EM procedure, we prevented the sample covariance estimators from exiting the cone of positive semi‐definite matrices during iterations and took Moore–Penrose pseudoinverses with eigenvalues below $10^{-10}$ regarded as 0. Both of these modifications did indeed improve the algorithm, but some instability persists. Moreover, use of the SWEEP operator (Beaton, 1964), which is designed to compute the Schur complement in a numerically stable way, failed to remedy the situation, yielding identical (increasing) log‐likelihood trajectories as the vanilla algorithm.

5 Each iteration of the EM algorithm involves the inversion of n matrices, where the dimension of the ith such matrix is $\sum_{j=1}^{d}\omega_{ij}\times \sum_{j=1}^{d}\omega_{ij}$ (i.e. O(d)×O(d)). Using standard matrix inversion algorithms, then, each iteration has computational complexity of order $nd^{3}$, and moreover the number of iterations required for numerical convergence can be very large in higher dimensions. This meant that even when d=100, primePCA was nearly 50 times faster than the EM algorithm.

6
https://www.kaggle.com/c/msdchallenge/data
