# Supplementary material for: High‐dimensional principal component analysis with heterogeneous missingness
Source: J R Stat Soc Series B Stat Methodol. 2022 Nov 20;84(5):2000–31. doi: 10.1111/rssb.12550 (PMC10098677; doi:10.1111/rssb.12550)
Supplement: Supplementary file 1 — Data S1: Supporting information [file RSSB-84-2000-s001.pdf]

Supplementary material for "High-dimensional principal component analysis with heterogeneous missingness"

Ziwei Zhu[*,‡], Tengyao Wang[*,†] and Richard J. Samworth[*]
[*] *Statistical Laboratory, University of Cambridge*
[†] *Department of Statistics, London School of Economics*
[‡] *Department of Statistics, University of Michigan, Ann Arbor*
E-mail: ziweiz@umich.edu, t.wang59@lse.ac.uk, r.samworth@statslab.cam.ac.uk

A. Proof of main results

In this section, we give the proofs of our main results stated in Zhu, Wang and Samworth (2021), hereafter referred to as the main text. Auxiliary lemmas, together with their proofs, are deferred to Section B.

We define two linear maps $\mathcal{D}, \mathcal{F} : \mathbb{R}^{d\times d} \to \mathbb{R}^{d\times d}$, such that for any $\mathbf{A} = (A_{ij}) \in \mathbb{R}^{d\times d}$, we have $[\mathcal{D}(\mathbf{A})]_{ij} := A_{ij}\mathbb{1}_{\{i=j\}}$ and $\mathcal{F}(\mathbf{A}) := \mathbf{A} - \mathcal{D}(\mathbf{A})$. In other words, $\mathcal{D}(\mathbf{A})$ and $\mathcal{F}(\mathbf{A})$ correspond to the diagonal and off-diagonal parts of $\mathbf{A}$ respectively. For $j \in [d]$, let $\mathbf{e}_j \in \mathbb{R}^d$ denote the standard basis vector along the jth coordinate axis and let $\mathbf{1}_d$ denote the all-one vector in $\mathbb{R}^d$. Moreover, for $a, b \geq 0$, we write $a \lesssim b$ if there exists a universal constant $C > 0$ such that $a \leq Cb$, and, where a and b may depend on an additional variable x, say, we write $a \lesssim_x b$ if there exists $C > 0$, depending only on x, such that $a \leq Cb$.

Proof (of Theorem 1). To simplify notation, we write $\widehat{\mathbf{V}}_K = \widehat{\mathbf{V}}_K^{\mathrm{OPW}}$ in this proof. Since $\mathbf{y}_i = \mathbf{V}_K\mathbf{u}_i + \mathbf{z}_i$, we have that

$$\|\mathbf{y}_i\|_{\psi_2} \leq \|\mathbf{V}_K\mathbf{u}_i\|_{\psi_2} + \|\mathbf{z}_i\|_{\psi_2} = \|\mathbf{u}_i\|_{\psi_2} + \|\mathbf{z}_i\|_{\psi_2} \leq (\lambda_1^{1/2} + 1)\tau. \tag{1}$$

Moreover, since $\max_{j\in[d]} \|y_{1j}\|_{\psi_2} \leq M^{1/2}$ by Lemma 1, it follows from van der Vaart and Wellner (1996, Lemma 2.2.2) that there exist a universal constant $C > 0$ such that†

$$\big\| \|\mathbf{y}_i\|_\infty \big\|_{\psi_2} \leq \{CM \log d\}^{1/2}. \tag{2}$$

Recall that $\widetilde{\mathbf{y}}_i^\top = (\widetilde{y}_{i1}, \ldots, \widetilde{y}_{id})$ denotes the ith row of $\mathbf{Y}_{\mathbf{\Omega}}$. Define $\mathbf{A}_i := \mathcal{F}(\widetilde{\mathbf{y}}_i\widetilde{\mathbf{y}}_i^\top)$ and $\mathbf{B}_i := \mathcal{D}(\widetilde{\mathbf{y}}_i\widetilde{\mathbf{y}}_i^\top)$. We have the following decomposition:

$$\begin{aligned}
\widehat{\mathbf{G}} &= \frac{1}{n}\sum_{i=1}^n \biggl(\frac{1}{\widehat{p}^2}\mathbf{A}_i - \frac{1}{p^2}\mathbb{E}\mathbf{A}_i\biggr) + \frac{1}{n}\sum_{i=1}^n \biggl(\frac{1}{\widehat{p}}\mathbf{B}_i - \frac{1}{p}\mathbb{E}\mathbf{B}_i\biggr) + \mathbf{\Sigma}_{\mathbf{y}} \\
&= \frac{1}{n\widehat{p}^2}\sum_{i=1}^n (\mathbf{A}_i - \mathbb{E}\mathbf{A}_i) + \frac{1}{n\widehat{p}}\sum_{i=1}^n (\mathbf{B}_i - \mathbb{E}\mathbf{B}_i) + \biggl(\frac{1}{\widehat{p}^2} - \frac{1}{p^2}\biggr)\mathbb{E}\mathbf{A}_1 + \biggl(\frac{1}{\widehat{p}} - \frac{1}{p}\biggr)\mathbb{E}\mathbf{B}_1 + \mathbf{\Sigma}_{\mathbf{y}} \\
&= \frac{1}{n\widehat{p}^2}\sum_{i=1}^n (\mathbf{A}_i - \mathbb{E}\mathbf{A}_i) + \frac{1}{n\widehat{p}}\sum_{i=1}^n (\mathbf{B}_i - \mathbb{E}\mathbf{B}_i) + \biggl(\frac{p^2}{\widehat{p}^2} - 1\biggr)\mathcal{F}(\mathbf{\Sigma}_{\mathbf{y}}) + \biggl(\frac{p}{\widehat{p}} - 1\biggr)\mathcal{D}(\mathbf{\Sigma}_{\mathbf{y}}) + \mathbf{\Sigma}_{\mathbf{y}} \\
&= \frac{1}{n\widehat{p}^2}\sum_{i=1}^n (\mathbf{A}_i - \mathbb{E}\mathbf{A}_i) + \frac{1}{n\widehat{p}}\sum_{i=1}^n (\mathbf{B}_i - \mathbb{E}\mathbf{B}_i) + \biggl(\frac{p}{\widehat{p}} - \frac{p^2}{\widehat{p}^2}\biggr)\mathcal{D}(\mathbf{\Sigma}_{\mathbf{y}}) + \frac{p^2}{\widehat{p}^2}\mathbf{\Sigma}_{\mathbf{y}}.
\end{aligned}$$

†In van der Vaart and Wellner (1996), the ψ_2-norm of a random variable is defined slightly differently as $\|X\|_{\psi_2} := \inf\{a : \mathbb{E}e^{(X/a)^2} \leq 2\}$. It can be shown (Vershynin, 2012, Lemma 5.5) that these two norms are equivalent.

We regard $\widehat{\mathbf{G}}$ as a perturbed version of $(p^2/\widehat{p}^2)\mathbf{\Sigma}_{\mathbf{y}}$. Applying Yu, Wang and Samworth (2015, Theorem 2), we have

$$\begin{aligned}
&L(\widehat{\mathbf{V}}_K, \mathbf{V}_K) \\
&\leq \frac{2K^{1/2}\widehat{p}^2}{p^2\lambda_K}\bigg\|\frac{1}{n\widehat{p}^2}\sum_{i=1}^n(\mathbf{A}_i - \mathbb{E}\mathbf{A}_i) + \frac{1}{n\widehat{p}}\sum_{i=1}^n(\mathbf{B}_i - \mathbb{E}\mathbf{B}_i) + \Big(\frac{p}{\widehat{p}} - \frac{p^2}{\widehat{p}^2}\Big)\mathcal{D}(\mathbf{\Sigma}_{\mathbf{y}})\bigg\|_{\mathrm{op}} \\
&\leq \frac{2K^{1/2}}{\lambda_K}\bigg(\bigg\|\frac{1}{np^2}\sum_{i=1}^n(\mathbf{A}_i - \mathbb{E}\mathbf{A}_i)\bigg\|_{\mathrm{op}} + \bigg\|\frac{\widehat{p}}{np^2}\sum_{i=1}^n(\mathbf{B}_i - \mathbb{E}\mathbf{B}_i)\bigg\|_{\mathrm{op}} + \bigg\|\Big(\frac{\widehat{p}}{p} - 1\Big)\mathcal{D}(\mathbf{\Sigma}_{\mathbf{y}})\bigg\|_{\mathrm{op}}\bigg). \qquad (3)
\end{aligned}$$

We will control the expectation of the three terms on the right-hand side of (3) separately. Define $\widehat{p}_i := d^{-1}\sum_{j=1}^d \omega_{ij}$. For notational simplicity, we write $\mathbb{P}'$ and $\mathbb{E}'$ respectively for the probability and expectation conditional on $(\widehat{p}_1, \ldots, \widehat{p}_n)$. Also, let $\widehat{p}_i^{(2)} := \mathbb{E}'(\omega_{i1}\omega_{i2})$ and $\widehat{p}_i^{(3)} := \mathbb{E}'(\omega_{i1}\omega_{i2}\omega_{i3})$ (if $d = 2$, then $\widehat{p}_i^{(3)} := 0$). For the first term, we apply a symmetrisation argument. Let $\{\mathbf{A}_i^*\}_{i=1}^n$ denote copies of $\{\mathbf{A}_i\}_{i=1}^n$ that are independent of $\{\mathbf{u}_i, \mathbf{z}_i, \boldsymbol{\omega}_i\}_{i=1}^n$, let $\{\epsilon_i\}_{i=1}^n$ be independent Rademacher random variables that are independent of $\{\mathbf{u}_i, \mathbf{z}_i, \boldsymbol{\omega}_i, \mathbf{A}_i^*\}_{i=1}^n$ and write $\mathbb{E}^*$ for expectation conditional on $\{\mathbf{u}_i, \mathbf{z}_i, \boldsymbol{\omega}_i\}_{i=1}^n$. Then by Jensen's inequality,

$$\begin{aligned}
\mathbb{E}\bigg\|\frac{1}{np^2}\sum_{i=1}^n(\mathbf{A}_i - \mathbb{E}\mathbf{A}_i)\bigg\|_{\mathrm{op}} &= \mathbb{E}\bigg\|\frac{1}{np^2}\sum_{i=1}^n(\mathbf{A}_i - \mathbb{E}^*\mathbf{A}_i^*)\bigg\|_{\mathrm{op}} \leq \mathbb{E}\bigg\|\frac{1}{np^2}\sum_{i=1}^n(\mathbf{A}_i - \mathbf{A}_i^*)\bigg\|_{\mathrm{op}} \\
&= \mathbb{E}\bigg\|\frac{1}{np^2}\sum_{i=1}^n\epsilon_i(\mathbf{A}_i - \mathbf{A}_i^*)\bigg\|_{\mathrm{op}} \leq 2\mathbb{E}\bigg\|\frac{1}{np^2}\sum_{i=1}^n\epsilon_i\mathbf{A}_i\bigg\|_{\mathrm{op}}. \qquad (4)
\end{aligned}$$

Since $\mathbf{A}_i = \widetilde{\mathbf{y}}_i\widetilde{\mathbf{y}}_i^\top - \mathcal{D}(\widetilde{\mathbf{y}}_i\widetilde{\mathbf{y}}_i^\top)$, we have that

$$\mathbb{E}'\{(\mathbf{A}_i^2)_{jk} \mid \mathbf{y}_i\} = \begin{cases} \mathbb{E}'\{\widetilde{y}_{ij}^2\|\widetilde{\mathbf{y}}_i\|_2^2 - \widetilde{y}_{ij}^4 \mid \mathbf{y}_i\} = \widehat{p}_i^{(2)}y_{ij}^2\sum_{t\neq j}y_{it}^2, & \text{if } j = k, \\ \sum_{t\notin\{j,k\}}\mathbb{E}'\{\widetilde{y}_{ij}\widetilde{y}_{ik}\widetilde{y}_{it}^2 \mid \mathbf{y}_i\} = \widehat{p}_i^{(3)}y_{ij}y_{ik}\sum_{t\notin\{j,k\}}y_{it}^2, & \text{if } j \neq k. \end{cases}$$

For two symmetric matrices $\mathbf{A}, \mathbf{B} \in \mathbb{R}^{d\times d}$, we write $\mathbf{A} \preceq \mathbf{B}$ if $\mathbf{B} - \mathbf{A}$ is positive semidefinite. Writing $\mathbf{y}_{i,-t} := \mathbf{y}_i - y_{it}\mathbf{e}_t$, we then have

$$\begin{aligned}
\mathbb{E}'(\mathbf{A}_i^2 \mid \mathbf{y}_i) &= \widehat{p}_i^{(3)}\sum_{t=1}^d y_{it}^2\mathbf{y}_{i,-t}\mathbf{y}_{i,-t}^\top + (\widehat{p}_i^{(2)} - \widehat{p}_i^{(3)})\mathcal{D}\Big(\sum_{t=1}^d y_{it}^2\mathbf{y}_{i,-t}\mathbf{y}_{i,-t}^\top\Big) \\
&\preceq \widehat{p}_i^{(3)}\|\mathbf{y}_i\|_\infty^2\sum_{t=1}^d\mathbf{y}_{i,-t}\mathbf{y}_{i,-t}^\top + (\widehat{p}_i^{(2)} - \widehat{p}_i^{(3)})\|\mathbf{y}_i\|_\infty^2\mathcal{D}\Big(\sum_{t=1}^d\mathbf{y}_{i,-t}\mathbf{y}_{i,-t}^\top\Big).
\end{aligned}$$

Notice that

$$\sum_{t=1}^d\mathbf{y}_{i,-t}\mathbf{y}_{i,-t}^\top = \sum_{t=1}^d\big(\mathbf{y}_i\mathbf{y}_i^\top - y_{it}\mathbf{e}_t\mathbf{y}_i^\top - y_{it}\mathbf{y}_i\mathbf{e}_t^\top + y_{it}^2\mathbf{e}_t\mathbf{e}_t^\top\big) = (d-2)\mathbf{y}_i\mathbf{y}_i^\top + \mathcal{D}(\mathbf{y}_i\mathbf{y}_i^\top).$$

Therefore,

$$\begin{aligned}
\mathbb{E}'(\mathbf{A}_i^2 \mid \mathbf{y}_i) &\preceq \|\mathbf{y}_i\|_\infty^2\big\{\widehat{p}_i^{(3)}(d-2)\mathbf{y}_i\mathbf{y}_i^\top + \big((d-1)\widehat{p}_i^{(2)} - (d-2)\widehat{p}_i^{(3)}\big)\mathcal{D}(\mathbf{y}_i\mathbf{y}_i^\top)\big\} \\
&\preceq d\|\mathbf{y}_i\|_\infty^2\big\{\widehat{p}_i^{(3)}\mathbf{y}_i\mathbf{y}_i^\top + \widehat{p}_i^{(2)}\mathcal{D}(\mathbf{y}_i\mathbf{y}_i^\top)\big\}.
\end{aligned}$$

Now, observe that $\|\mathbf{A}_i\|_{\mathrm{op}} \leq d\widehat{p}_i\|\mathbf{y}_i\|_\infty^2$, so for $q \geq 2$,

$$\mathbb{E}'\big(\mathbf{A}_i^q\big) \preceq \mathbb{E}'\big\{(d\widehat{p}_i\|\mathbf{y}_i\|_\infty^2)^{q-2}\mathbb{E}'(\mathbf{A}_i^2 \mid \mathbf{y}_i)\big\} \preceq d^{q-1}\widehat{p}_i^{q-2}\mathbb{E}'\big[\|\mathbf{y}_i\|_\infty^{2q-2}\big\{\widehat{p}_i^{(3)}\mathbf{y}_i\mathbf{y}_i^\top + \widehat{p}_i^{(2)}\mathcal{D}(\mathbf{y}_i\mathbf{y}_i^\top)\big\}\big].$$

By the Cauchy–Schwarz inequality, we therefore have that

$$\begin{aligned}\|\mathbb{E}'(\epsilon_i^q \mathbf{A}_i^q)\|_{\mathrm{op}} &\leq d^{q-1}\widehat{p}_i^{q-2}\widehat{p}_i^{(3)}\Bigl[\mathbb{E}\bigl(\|\mathbf{y}_i\|_\infty^{4q-4}\bigr)\sup_{\mathbf{v}\in\mathcal{S}^{d-1}}\mathbb{E}\bigl\{(\mathbf{v}^\top\mathbf{y}_i)^4\bigr\}\Bigr]^{1/2} + d^{q-1}\widehat{p}_i^{q-2}\widehat{p}_i^{(2)}\mathbb{E}\|\mathbf{y}_i\|_\infty^{2q} \\ &\leq d^{q-1}\widehat{p}_i^{q-2}\Bigl\{\widehat{p}_i^{(3)}(4q-4)^{q-1}(CM\log d)^{q-1}8R\tau^2 + \widehat{p}_i^{(2)}(2q)^q(CM\log d)^q\Bigr\} \\ &\leq \frac{q!}{2}\bigl\{32eCMR\tau^2\widehat{p}_i^{(3)}d\log d + e^2\widehat{p}_i^{(2)}d(CM\log d)^2\bigr\}\bigl(4eCM\widehat{p}_i d\log d\bigr)^{q-2} \\ &\leq \frac{q!}{2}C'Md\log d\bigl\{R\tau^2\widehat{p}_i^{(3)} + \widehat{p}_i^{(2)}M\log d\bigr\}\bigl(4eCM\widehat{p}_i d\log d\bigr)^{q-2},\end{aligned}$$

where $C' > 0$ is a universal constant, the second inequality uses (1) and (2) and the penultimate bound uses Stirling's inequality.

Let $\rho := 4eCMd(\max_i \widehat{p}_i)\log d$ and $\sigma^2 := C'Mn^{-1}d\log d\sum_{i=1}^n\bigl\{R\tau^2\widehat{p}_i^{(3)} + \widehat{p}_i^{(2)}M\log d\bigr\}$. Then by Tropp (2012, Theorem 6.2), we obtain that

$$\mathbb{P}'\biggl(\biggl\|\frac{1}{n}\sum_{i=1}^n\epsilon_i\mathbf{A}_i\biggr\|_{\mathrm{op}} \geq t\biggr) \leq 2d\exp\biggl(\frac{-nt^2/2}{\sigma^2+\rho t}\biggr).$$

Consequently, for $t_0 := 2\sigma n^{-1/2}\log^{1/2} d + 4\rho n^{-1}\log d$, we have

$$\mathbb{E}'\biggl\|\frac{1}{n}\sum_{i=1}^n\epsilon_i\mathbf{A}_i\biggr\|_{\mathrm{op}} \leq t_0 + \int_{t_0}^\infty 2d\bigl\{e^{-nt^2/(4\sigma^2)} + e^{-nt/(4\rho)}\bigr\}\,dt \leq 4t_0.$$

Given (4), integrating the left-hand side of the above inequality over $(\widehat{p}_i)_{i=1}^n$ yields

$$\begin{aligned}\mathbb{E}\biggl\|\frac{1}{np^2}\sum_{i=1}^n(\mathbf{A}_i - \mathbb{E}\mathbf{A}_i)\biggr\|_{\mathrm{op}} &\lesssim \frac{(\mathbb{E}\sigma^2)^{1/2}\log^{1/2} d}{n^{1/2}p^2} + \frac{\mathbb{E}\rho\log d}{np^2} \\ &\lesssim \sqrt{\frac{Md\{R\tau^2 p + M\log d\}\log^2 d}{np^2}} + \frac{Md\log^2 d\log n}{np},\end{aligned} \tag{5}$$

where the first inequality uses Jensen's inequality and the second inequality uses Lemma 4.

For the second sum on the right-hand side of (3), we have by van der Vaart and Wellner (1996, Lemma 2.2.2) again that

$$\begin{aligned}\biggl\|\biggl\|\frac{1}{n}\sum_{i=1}^n(\mathbf{B}_i - \mathbb{E}\mathbf{B}_i)\biggr\|_{\mathrm{op}}\biggr\|_{\psi_1} &= \biggl\|\max_{j\in[d]}\biggl|\frac{1}{n}\sum_{i=1}^n(\widetilde{y}_{ij}^2 - \mathbb{E}\widetilde{y}_{ij}^2)\biggr|\biggr\|_{\psi_1} \\ &\lesssim \frac{\log d}{n}\biggl\|\sum_{i=1}^n(\widetilde{y}_{i1}^2 - \mathbb{E}\widetilde{y}_{i1}^2)\biggr\|_{\psi_1} \lesssim \frac{M\log d}{\sqrt{n}},\end{aligned}$$

where the final inequality uses Lemma 2 and the fact that $\|\widetilde{y}_{i1}^2 - \mathbb{E}\widetilde{y}_{i1}^2\|_{\psi_1} \leq \|\widetilde{y}_{i1}^2\|_{\psi_1} + \mathbb{E}\widetilde{y}_{i1}^2 \leq 2M$.

Now by the Cauchy–Schwarz inequality,

$$\begin{aligned}\mathbb{E}\biggl\|\frac{\widehat{p}}{np^2}\sum_{i=1}^n(\mathbf{B}_i - \mathbb{E}\mathbf{B}_i)\biggr\|_{\mathrm{op}} &\leq \biggl\{\mathbb{E}\biggl(\frac{\widehat{p}^2}{p^4}\biggr)\mathbb{E}\biggl(\biggl\|\frac{1}{n}\sum_{i=1}^n(\mathbf{B}_i - \mathbb{E}\mathbf{B}_i)\biggr\|_{\mathrm{op}}^2\biggr)\biggr\}^{1/2} \\ &\lesssim \biggl\{\biggl(\frac{1}{p^2} + \frac{1}{ndp^3}\biggr)\frac{M^2\log^2 d}{n}\biggr\}^{1/2} \lesssim \frac{M\log d}{p\sqrt{n}},\end{aligned} \tag{6}$$

which is dominated by the bound in (5).

Finally, for the third term on the right-hand side of (3), we have by the Cauchy–Schwarz inequality again that

$$\mathbb{E}\biggl\|\biggl(\frac{\widehat{p}}{p} - 1\biggr)\mathcal{D}(\mathbf{\Sigma}_{\mathbf{y}})\biggr\|_{\mathrm{op}} \lesssim \frac{M}{\sqrt{ndp}}, \tag{7}$$

which is also dominated by the bound in (5). Substituting (5), (6) and (7) into (3) establishes (5) in the main text.

If we regard M and τ as constants and if $n \geq d \log^2 d \log^2 n/(\lambda_1 p + \log d)$, then the second term in the curly bracket of the right-hand side of (5) in the main text is dominated up to a constant by the first term, and claim (6) in the main text follows immediately.

Proof (of Theorem 2). Without loss of generality, we may assume that $d \geq 50$ and that d is even, and write $d = 2h$ for some $h \in \mathbb{N}$. By the Gilbert–Varshamov lemma (see, e.g. Massart, 2007, Lemma 4.7), there exist $W \subseteq \{0,1\}^h$ such that $\log|W| \geq h/16$ and for any distinct pair of vectors $\mathbf{w}, \mathbf{w}' \in W$, their Hamming distance, denoted by $d_{\mathrm{H}}(\mathbf{w},\mathbf{w}')$, is at least $h/4$. Let $\gamma \in [0, \pi/2]$ be a real number to be specified later. Recall also that the Kronecker product of two matrices $\mathbf{A} = (A_{ij}) \in \mathbb{R}^{d_1 \times d_2}$ and $\mathbf{B} = (B_{ij}) \in \mathbb{R}^{d_1' \times d_2'}$ is defined as the block matrix

$$\mathbf{A} \otimes \mathbf{B} := \begin{pmatrix} A_{11}\mathbf{B} & \cdots & A_{1d_2}\mathbf{B} \\ \vdots & \ddots & \vdots \\ A_{d_1 1}\mathbf{B} & \cdots & A_{d_1 d_2}\mathbf{B} \end{pmatrix} \in \mathbb{R}^{d_1 d_1' \times d_2 d_2'}.$$

To each $\mathbf{w} \in W$, we can associate a distribution $P_{\mathbf{w}} \in \mathcal{P}_{n,d}(\lambda_1, p)$ such that $\mathbf{U}$ is a random vector ($n \times 1$ random matrix) with independent $N(0,\lambda_1)$ entries, $\mathbf{Z}$ is an $n \times d$ random matrix with independent $N(0,1)$ entries, and

$$\mathbf{V}_1 = \mathbf{V}_{1,\mathbf{w}} := \frac{1}{\sqrt{h}}\biggl\{\mathbf{w} \otimes \begin{pmatrix} \cos\gamma \\ \sin\gamma \end{pmatrix} + (\mathbf{1}_h - \mathbf{w}) \otimes \begin{pmatrix} \cos\gamma \\ -\sin\gamma \end{pmatrix}\biggr\} \in \mathcal{S}^{d-1}.$$

Fixing distinct $\mathbf{w}, \mathbf{w}' \in W$, we write $\mathbf{v} = (v_j)_{j\in[d]} := \mathbf{V}_{1,\mathbf{w}}$ and $\mathbf{v}' = (v_j')_{j\in[d]} := \mathbf{V}_{1,\mathbf{w}'}$ and let $Q_{\mathbf{w}}$ and $Q_{\mathbf{w}'}$ denote respectively the marginal distribution of $(\tilde{\mathbf{y}}_1, \boldsymbol{\omega}_1)$ under $P_{\mathbf{w}}$ and $P_{\mathbf{w}'}$. Define $S := \{j \in [d] : \omega_{1j} = 1\}$ and also set $\bar{\mathbf{v}}_S := (v_j \mathbb{1}_{\{j\in S\}})_{j\in[d]} \in \mathbb{R}^d$ and $\bar{\mathbf{v}}_S' := (v_j' \mathbb{1}_{\{j\in S\}})_{j\in[d]} \in \mathbb{R}^d$. Then the Kullback–Leibler divergence‡ from $P_{\mathbf{w}'}$ to $P_{\mathbf{w}}$ is given by

$$\begin{aligned}\mathrm{KL}(P_{\mathbf{w}}, P_{\mathbf{w}'}) &= \mathrm{KL}(Q_{\mathbf{w}}^{\otimes n}, Q_{\mathbf{w}'}^{\otimes n}) = n\mathrm{KL}(Q_{\mathbf{w}}, Q_{\mathbf{w}'}) = n\mathbb{E}_{Q_{\mathbf{w}}}\biggl\{\mathbb{E}_{Q_{\mathbf{w}}}\biggl(\log\frac{dQ_{\mathbf{w}}}{dQ_{\mathbf{w}'}}\,\bigg|\,\boldsymbol{\omega}_1\biggr)\biggr\} \\ &= n\mathbb{E}\,\mathrm{KL}\bigl(N_d(\mathbf{0}, \mathbf{I}_d + \lambda_1\bar{\mathbf{v}}_S\bar{\mathbf{v}}_S^\top), N_d(\mathbf{0}, \mathbf{I}_d + \lambda_1\bar{\mathbf{v}}_S'\bar{\mathbf{v}}_S'^\top)\bigr), \qquad (8)\end{aligned}$$

where the final expectation is over the marginal distribution of S under $P_{\mathbf{w}}$. We partition $S = S_0 \sqcup S_{1+} \sqcup S_{1-}$, where $S_0 := \{j \in S : j \text{ is odd}\}$, $S_{1+} := \{j \in S : j \text{ is even and } v_j = \bar{v}_j'\}$ and $S_{1-} := \{j \in S : j \text{ is even and } v_j \neq v_j'\}$. Since by construction we always have $\|\bar{\mathbf{v}}_S\|_2 = \|\bar{\mathbf{v}}_S'\|_2$, we can apply Lemma 5 to obtain

$$\begin{aligned}\mathrm{KL}\bigl(N(\mathbf{0}, \mathbf{I}_d + \lambda_1\bar{\mathbf{v}}_S\bar{\mathbf{v}}_S^\top), N(\mathbf{0}, \mathbf{I}_d + \lambda_1\bar{\mathbf{v}}_S'(\bar{\mathbf{v}}_S')^\top)\bigr) &= \frac{\lambda_1^2(\|\bar{\mathbf{v}}_S\|_2^4 - \langle\bar{\mathbf{v}}_S, \bar{\mathbf{v}}_S'\rangle^2)}{2(1+\lambda_1\|\bar{\mathbf{v}}_S\|_2^2)} \\ \leq \frac{\lambda_1^2\langle\bar{\mathbf{v}}_S, \bar{\mathbf{v}}_S + \bar{\mathbf{v}}_S'\rangle\langle\bar{\mathbf{v}}_S, \bar{\mathbf{v}}_S - \bar{\mathbf{v}}_S'\rangle}{2\max\{1, \lambda_1\|\bar{\mathbf{v}}_S\|_2^2\}} &= \frac{\lambda_1^2\bigl(\sum_{j\in S_0\cup S_{1+}} 2v_j^2\bigr)\bigl(\sum_{j\in S_{1-}} 2v_j^2\bigr)}{2\max\{1, \lambda_1\sum_{j\in S} v_j^2\}} \\ \leq \min\biggl\{\frac{2\lambda_1^2}{h^2}\bigl(|S_0 \times S_{1-}|\sin^2\gamma\cos^2\gamma + |S_{1+}\times S_{1-}|\sin^4\gamma\bigr), \frac{2\lambda_1|S_{1-}|\sin^2\gamma}{h}\biggr\}.\end{aligned}$$

Substituting the above bound into (8), we have

$$\mathrm{KL}(P_{\mathbf{w}}, P_{\mathbf{w}'}) \leq 2n\lambda_1 p\min\{1, \lambda_1 p\}\sin^2\gamma. \qquad (9)$$

On the other hand, since $d_{\mathrm{H}}(\mathbf{w},\mathbf{w}') \geq h/4$, we also have

$$\sin^2\Theta(\mathbf{v}, \mathbf{v}') = 1 - (\mathbf{v}^\top\mathbf{v}')^2 = 1 - \biggl(1 - \frac{2d_{\mathrm{H}}(\mathbf{w},\mathbf{w}')\sin^2\gamma}{h}\biggr)^2 \geq \frac{1}{2}\sin^2\gamma. \qquad (10)$$

‡Recall that for two distributions P_1 and P_2 defined on the same measurable space $(\mathcal{X}, \mathcal{A})$ and such that P_1 is absolutely continuous with respect to P_2, the Kullback–Leibler divergence from P_2 to P_1 is given by $\mathrm{KL}(P_1, P_2) := \int_{\mathcal{X}} \log\frac{dP_1}{dP_2}\,dP_1$.

By (9), (10) and Fano's inequality (Yu, 1997, Lemma 3),

$$\inf_{\widehat{\mathbf{v}}} \sup_{P\in\mathcal{P}_{n,d,1}(\lambda_1,p)} \mathbb{E}_P L(\widehat{\mathbf{v}},\mathbf{v}) \geq \inf_{\widehat{\mathbf{v}}} \max_{\mathbf{w}\in W} \mathbb{E}_{P_{\mathbf{w}}} L(\widehat{\mathbf{v}},\mathbf{v})$$
$$\geq \frac{1}{2\sqrt{2}}\sin\gamma\biggl(1-\frac{\log 2+2n\lambda_1 p\min\{1,\lambda_1 p\}\sin^2\gamma}{\log|W|}\biggr).$$

We now choose $\gamma\in[0,\pi/2]$ such that $\sin^2\gamma=\min\bigl\{\frac{\log|W|}{8n\lambda_1 p\min\{1,\lambda_1 p\}},1\bigr\}$. Since $d\geq 50$, we obtain $\log|W|\geq d/32\geq 2\log 2$. Therefore,

$$\inf_{\widehat{\mathbf{v}}} \sup_{P\in\mathcal{P}_{n,d,1}(\lambda_1,p)} \mathbb{E}_P L(\widehat{\mathbf{v}},\mathbf{v}) \geq \frac{1}{8\sqrt{2}}\sin\gamma \geq \min\biggl\{\frac{1}{200\lambda_1}\sqrt{\frac{d\max(1,\lambda_1 p)}{np^2}},\frac{1}{8\sqrt{2}}\biggr\},$$

as desired.

Proof (of Proposition 1). For notational simplicity, we write $\widehat{\mathbf{V}}_K:=\widehat{\mathbf{V}}_K^{(\mathrm{in})}$ and $\widehat{\mathbf{V}}_{S,K}:=(\widehat{\mathbf{V}}_K)_S$ for any $S\subseteq[d]$, and let $\mathbf{W}\in\mathbb{O}^{K\times K}$ be the solution to the Procrustes problem for $\mathbf{V}_K$ and $\mathbf{R}$, so that $\mathbf{W}=\mathrm{argmin}_{\mathbf{O}\in\mathbb{O}^{K\times K}}\|\widehat{\mathbf{V}}_K-\mathbf{R}\mathbf{O}\|_{\mathrm{F}}$ and $\|\widehat{\mathbf{V}}_K-\mathbf{R}\mathbf{W}\|_{2\to\infty}=\mathcal{T}(\widehat{\mathbf{V}}_K,\mathbf{R})$ (see the discussion around (7) in the main text). For $i\in\mathcal{I}$, let $\boldsymbol{\ell}_i^\top\in\mathbb{R}^K$ denote the ith row of $\mathbf{L}$. For any $i\in\mathcal{I}$, we have $\widehat{\mathbf{y}}_{i,\mathcal{J}_i}=\mathbf{y}_{i,\mathcal{J}_i}$ and

$$\begin{aligned}\widehat{\mathbf{y}}_{i,\mathcal{J}_i^c}-\mathbf{y}_{i,\mathcal{J}_i^c}&=\widehat{\mathbf{V}}_{\mathcal{J}_i^c,K}(\widehat{\mathbf{V}}_{\mathcal{J}_i,K}^\top\widehat{\mathbf{V}}_{\mathcal{J}_i,K})^{-1}\widehat{\mathbf{V}}_{\mathcal{J}_i,K}^\top\mathbf{y}_{i,\mathcal{J}_i}-\mathbf{y}_{i,\mathcal{J}_i^c}\\&=\widehat{\mathbf{V}}_{\mathcal{J}_i^c,K}(\widehat{\mathbf{V}}_{\mathcal{J}_i,K}^\top\widehat{\mathbf{V}}_{\mathcal{J}_i,K})^{-1}\widehat{\mathbf{V}}_{\mathcal{J}_i,K}^\top\mathbf{R}_{\mathcal{J}_i}\mathbf{W}\mathbf{W}^{-1}\boldsymbol{\Gamma}\boldsymbol{\ell}_i-\mathbf{R}_{\mathcal{J}_i^c}\boldsymbol{\Gamma}\boldsymbol{\ell}_i\\&=\widehat{\mathbf{V}}_{\mathcal{J}_i^c,K}(\widehat{\mathbf{V}}_{\mathcal{J}_i,K}^\top\widehat{\mathbf{V}}_{\mathcal{J}_i,K})^{-1}\widehat{\mathbf{V}}_{\mathcal{J}_i,K}^\top(\mathbf{R}_{\mathcal{J}_i}\mathbf{W}-\widehat{\mathbf{V}}_{\mathcal{J}_i,K})\mathbf{W}^{-1}\boldsymbol{\Gamma}\boldsymbol{\ell}_i+(\widehat{\mathbf{V}}_{\mathcal{J}_i^c,K}-\mathbf{R}_{\mathcal{J}_i^c}\mathbf{W})\mathbf{W}^{-1}\boldsymbol{\Gamma}\boldsymbol{\ell}_i.\end{aligned}$$

Thus

$$\begin{aligned}\|\widehat{\mathbf{y}}_{i,\mathcal{J}_i^c}-\mathbf{y}_{i,\mathcal{J}_i^c}\|_\infty&\leq\sigma_*\sqrt{d}\|\widehat{\mathbf{V}}_{\mathcal{J}_i^c,K}\|_{2\to\infty}\|\mathbf{R}_{\mathcal{J}_i}\mathbf{W}-\widehat{\mathbf{V}}_{\mathcal{J}_i,K}\|_{2\to\infty}\|\boldsymbol{\Gamma}\boldsymbol{\ell}_i\|_2+\|\widehat{\mathbf{V}}_{\mathcal{J}_i^c,K}-\mathbf{R}_{\mathcal{J}_i^c}\mathbf{W}\|_{2\to\infty}\|\boldsymbol{\Gamma}\boldsymbol{\ell}_i\|_2\\&\leq\Delta\|\boldsymbol{\Gamma}\boldsymbol{\ell}_i\|_2\bigl(1+\sigma_*\sqrt{d}\|\widehat{\mathbf{V}}_K\|_{2\to\infty}\bigr)\\&\leq\Delta\sigma_1(\boldsymbol{\Gamma})\mu\Bigl(\frac{K}{n}\Bigr)^{1/2}\bigl\{1+\sigma_*\bigl(\mu\sqrt{K}+\Delta\sqrt{d}\bigr)\bigr\}\\&\leq\frac{C'}{n^{1/2}}\Delta\sigma_1(\boldsymbol{\Gamma})\mu^2K=:m,\end{aligned}$$

say, where $C'>0$ depends only on σ_* and c_1. Note that the inequality above holds for all $i\in\mathcal{I}$. Writing $\mathbf{E}:=\widehat{\mathbf{Y}}-\mathbf{Y}$ for convenience, we have found that $\|\mathbf{E}\|_\infty\leq m$. Let $\mathbf{L}_\perp\in\mathbb{O}^{n\times(n-K)},\mathbf{R}_\perp\in\mathbb{O}^{d\times(d-K)}$ be the orthogonal complements of $\mathbf{L}\in\mathbb{O}^{n\times K}$ and $\mathbf{R}\in\mathbb{O}^{d\times K}$ respectively, so that $(\mathbf{L},\mathbf{L}_\perp)\in\mathbb{O}^{n\times n}$ and $(\mathbf{R},\mathbf{R}_\perp)\in\mathbb{O}^{d\times d}$. We wish to apply Cai and Zhang (2018a, Theorem 1). To this end, note that

$$\|\mathbf{L}^\top\mathbf{E}\mathbf{R}\|_{\mathrm{op}}=\sup_{\mathbf{s},\mathbf{t}\in\mathcal{S}^{K-1}}(\mathbf{L}\mathbf{s})^\top\mathbf{E}(\mathbf{R}\mathbf{t})\leq\|\mathbf{L}\|_{2\to\infty}\|\mathbf{R}\|_{2\to\infty}\|\mathbf{E}\|_1\leq\frac{K\mu^2m\|\boldsymbol{\Omega}^{\mathrm{c}}\|_1}{\sqrt{nd}}.$$

Hence, writing $\alpha:=\sigma_K(\boldsymbol{\Gamma}+\mathbf{L}^\top\mathbf{E}\mathbf{R})$, we have by Weyl's inequality that

$$\sigma_K(\boldsymbol{\Gamma})-\frac{K\mu^2m\|\boldsymbol{\Omega}^{\mathrm{c}}\|_1}{\sqrt{nd}}\leq\alpha\leq\sigma_K(\boldsymbol{\Gamma})+\frac{K\mu^2m\|\boldsymbol{\Omega}^{\mathrm{c}}\|_1}{\sqrt{nd}}.$$

Now, writing $\beta:=\|\mathbf{L}_\perp^\top\widehat{\mathbf{Y}}\mathbf{R}_\perp\|_{\mathrm{op}}=\|\mathbf{L}_\perp^\top\mathbf{E}\mathbf{R}_\perp\|_{\mathrm{op}}$, we have

$$\beta\leq\|\mathbf{E}\|_{\mathrm{op}}\leq\|\mathbf{E}\|_{\mathrm{F}}\leq m\sqrt{\|\boldsymbol{\Omega}^{\mathrm{c}}\|_1}.$$

In addition, by Cauchy–Schwarz and Jensen's inequality,

$$\|\mathbf{L}^\top\mathbf{E}\|_{\mathrm{op}}=\sup_{\substack{\mathbf{s}\in\mathcal{S}^{K-1}\\\mathbf{t}\in\mathcal{S}^{d-1}}}(\mathbf{L}\mathbf{s})^\top\mathbf{E}\mathbf{t}\leq\|\mathbf{L}\|_{2\to\infty}\sup_{\mathbf{t}\in\mathcal{S}^{K-1}}\|\mathbf{E}\mathbf{t}\|_1$$
$$\leq\mu(Kn)^{1/2}\frac{1}{n}\sum_{i=1}^n m\sqrt{\|\boldsymbol{\omega}_i^{\mathrm{c}}\|_1}\leq\mu m(K\|\boldsymbol{\Omega}^{\mathrm{c}}\|_1)^{1/2}.$$

Similarly,

$$\|\mathbf{E}\mathbf{R}\|_{\mathrm{op}} \leq \mu m(K\|\boldsymbol{\Omega}^{\mathrm{c}}\|_1)^{1/2}.$$

Hence there exists $c_1 > 0$, depending only on σ_*, such that whenever $\Delta \leq \frac{c_1\sigma_K(\boldsymbol{\Gamma})}{K^2\mu^4\sigma_1(\boldsymbol{\Gamma})\sqrt{d}}$, we have

$$\alpha^2 - \beta^2 - \min(\|\mathbf{L}^\top\mathbf{E}\|_{\mathrm{op}}^2, \|\mathbf{E}\mathbf{R}\|_{\mathrm{op}}^2) \geq \frac{\sigma_K^2(\boldsymbol{\Gamma})}{2} \quad \text{and} \quad \alpha, \beta \leq 2\sigma_K(\boldsymbol{\Gamma}).$$

Now let $\widehat{\mathbf{Y}} = \widehat{\mathbf{L}}\widehat{\boldsymbol{\Gamma}}\widehat{\mathbf{R}}^\top$ be an SVD of $\widehat{\mathbf{Y}}$. We can now apply Cai and Zhang (2018a, Theorem 1) to deduce that for such c_1,

$$\begin{aligned}\|\sin\Theta(\widehat{\mathbf{R}}, \mathbf{R})\|_{\mathrm{op}} &\leq \frac{\alpha\|\mathbf{L}^\top\mathbf{E}\|_{\mathrm{op}} + \beta\|\mathbf{E}\mathbf{R}\|_{\mathrm{op}}}{\alpha^2 - \beta^2 - \min(\|\mathbf{L}^\top\mathbf{E}\|_{\mathrm{op}}^2, \|\mathbf{E}\mathbf{R}\|_{\mathrm{op}}^2)} \leq \frac{8m\mu(K\|\boldsymbol{\Omega}^{\mathrm{c}}\|_1)^{1/2}}{\sigma_K(\boldsymbol{\Gamma})} \\ &\leq \frac{8C'K^{3/2}\sigma_1(\boldsymbol{\Gamma})\mu^3}{\sigma_K(\boldsymbol{\Gamma})}\left(\frac{\|\boldsymbol{\Omega}^{\mathrm{c}}\|_1}{n}\right)^{1/2}\Delta =: \kappa\Delta,\end{aligned}$$

say. Similarly,

$$\|\sin\Theta(\widehat{\mathbf{L}}, \mathbf{L})\|_{\mathrm{op}} \leq \frac{\alpha\|\mathbf{E}\mathbf{R}\|_{\mathrm{op}} + \beta\|\mathbf{L}^\top\mathbf{E}\|_{\mathrm{op}}}{\alpha^2 - \beta^2 - \min(\|\mathbf{L}^\top\mathbf{E}\|_{\mathrm{op}}^2, \|\mathbf{E}\mathbf{R}\|_{\mathrm{op}}^2)} \leq \kappa\Delta.$$

We are now in a position to show contraction in terms of two-to-infinity norm. By Cape, Tang and Priebe (2018, Theorem 3.7),

$$\begin{aligned}\mathcal{T}(\widehat{\mathbf{R}}, \mathbf{R}) \leq \frac{2\|\mathbf{R}_\perp\mathbf{R}_\perp^\top\mathbf{E}^\top\mathbf{L}\mathbf{L}^\top\|_{2\to\infty}}{\sigma_K(\boldsymbol{\Gamma})} &+ \frac{2\|\mathbf{R}_\perp\mathbf{R}_\perp^\top\mathbf{E}^\top\mathbf{L}_\perp\mathbf{L}_\perp^\top\|_{2\to\infty}}{\sigma_K(\boldsymbol{\Gamma})}\|\sin\Theta(\widehat{\mathbf{L}}, \mathbf{L})\|_{\mathrm{op}} \\ &+ \|\sin\Theta(\widehat{\mathbf{R}}, \mathbf{R})\|_{\mathrm{op}}^2\|\mathbf{R}\|_{2\to\infty} =: T_1 + T_2 + T_3, \qquad (11)\end{aligned}$$

say. Note that

$$\begin{aligned}\|\mathbf{R}_\perp\mathbf{R}_\perp^\top\|_{\infty\to\infty} &\leq \|\mathbf{I}_d\|_{\infty\to\infty} + \|\mathbf{R}\mathbf{R}^\top\|_{\infty\to\infty} = 1 + \sup_{\|\mathbf{v}\|_\infty \leq 1}\|\mathbf{R}\mathbf{R}^\top\mathbf{v}\|_\infty \\ &\leq 1 + \sup_{\|\mathbf{v}\|_2 \leq \sqrt{d}}\|\mathbf{R}\|_{2\to\infty}\|\mathbf{R}^\top\mathbf{v}\|_2 \leq 1 + \sqrt{K}\mu.\end{aligned}$$

Hence,

$$\begin{aligned}T_1 &\leq \frac{2(1+\sqrt{K}\mu)\|\mathbf{E}^\top\mathbf{L}\mathbf{L}^\top\|_{2\to\infty}}{\sigma_K(\boldsymbol{\Gamma})} \leq \frac{2(1+\sqrt{K}\mu)\|\mathbf{E}^\top\mathbf{L}\|_{2\to\infty}}{\sigma_K(\boldsymbol{\Gamma})} \\ &\leq \frac{2(1+\sqrt{K}\mu)\mu\sqrt{K}m\|\boldsymbol{\Omega}^{\mathrm{c}}\|_{1\to 1}}{\sqrt{n}\sigma_K(\boldsymbol{\Gamma})} \lesssim \frac{K^2\mu^4\sigma_1(\boldsymbol{\Gamma})\|\boldsymbol{\Omega}^{\mathrm{c}}\|_{1\to 1}\Delta}{n\sigma_K(\boldsymbol{\Gamma})}.\end{aligned}$$

Moreover,

$$\begin{aligned}T_2 &\leq \frac{2(1+\sqrt{K}\mu)\|\mathbf{E}^\top\|_{2\to\infty}\kappa\Delta}{\sigma_K(\boldsymbol{\Gamma})} \leq \frac{2(1+\sqrt{K}\mu)m\|\boldsymbol{\Omega}^{\mathrm{c}}\|_{1\to 1}^{1/2}\kappa\Delta}{\sigma_K(\boldsymbol{\Gamma})} \\ &\lesssim \frac{K^{3/2}\mu^3\sigma_1(\boldsymbol{\Gamma})\|\boldsymbol{\Omega}^{\mathrm{c}}\|_{1\to 1}^{1/2}\kappa\Delta^2}{\sqrt{n}\sigma_K(\boldsymbol{\Gamma})}.\end{aligned}$$

Finally,

$$T_3 \leq \mu\kappa^2\Delta^2\left(\frac{K}{d}\right)^{1/2}.$$

Write

$$\eta := \frac{K^2\sigma_1(\boldsymbol{\Gamma})\|\boldsymbol{\Omega}^{\mathrm{c}}\|_{1\to 1}^{1/2}}{\sqrt{n}\sigma_K(\boldsymbol{\Gamma})}$$

for simplicity, so that $\kappa \lesssim_{\sigma_*} \mu^3(d/K)^{1/2}\eta$. Given that $\mathcal{T}(\widehat{\mathbf{V}}_K^{(\mathrm{out})}, \mathbf{V}_K) = \mathcal{T}(\widehat{\mathbf{R}}, \mathbf{R})$, substituting the bounds for T_1, T_2, T_3 into (11) yields that

$$\begin{aligned}\mathcal{T}(\widehat{\mathbf{V}}_K^{(\mathrm{out})}, \mathbf{V}_K) &\lesssim_{\sigma_*} \left\{\mu^4\eta\left(\frac{\|\mathbf{\Omega}^{\mathrm{c}}\|_{1\to1}}{n}\right)^{1/2} + \mu^6\frac{d^{1/2}}{K}\eta^2\Delta + \mu^7\left(\frac{d}{K}\right)^{1/2}\eta^2\Delta\right\}\Delta \\ &\le \eta^2\left\{\frac{\sigma_K(\mathbf{\Gamma})\mu^4}{K^2\sigma_1(\mathbf{\Gamma})} + 2\mu^7\left(\frac{d}{K}\right)^{1/2}\Delta\right\}\Delta \lesssim \frac{\mu^4\eta^2\sigma_K(\mathbf{\Gamma})}{K^2\sigma_1(\mathbf{\Gamma})}\Delta \\ &= \frac{\mu^4K^2\sigma_1(\mathbf{\Gamma})\|\mathbf{\Omega}^{\mathrm{c}}\|_{1\to1}}{\sigma_K(\mathbf{\Gamma})n}\Delta,\end{aligned}$$

as desired.

Proof (of Theorem 3). We prove this result by induction on t. The case $t = 0$ is true by definition of Δ, so suppose that the conclusion holds for some $t \in \{0\} \cup [n_{\mathrm{iter}} - 1]$. We make the following two claims:

(a) $\mathcal{I}^{(t)} = \mathcal{I}$;

(b) The error is further contracted by refinement, i.e., $\mathcal{T}(\widehat{\mathbf{V}}_K^{(t+1)}, \mathbf{V}_K) \le \rho\mathcal{T}(\widehat{\mathbf{V}}_K^{(t)}, \mathbf{V}_K)$.

To prove claim (a), similarly to the proof of Proposition 1, let $\mathbf{W} \in \mathbb{O}^{K\times K}$ be the solution to the Procrustes problem for $\widehat{\mathbf{V}}_K^{(t)}$ and $\mathbf{V}_K$. Notice that for each $i \in [n]$, by Weyl's inequality and the inductive hypothesis,

$$\begin{aligned}\big|\sigma_K\big((\widehat{\mathbf{V}}_K^{(t)})_{\mathcal{J}_i}\big) - \sigma_K((\mathbf{V}_K)_{\mathcal{J}_i})\big| &= \big|\sigma_K\big((\widehat{\mathbf{V}}_K^{(t)})_{\mathcal{J}_i}\big) - \sigma_K\big((\mathbf{V}_K)_{\mathcal{J}_i}\mathbf{W}\big)\big| \\ &\le \big\|(\widehat{\mathbf{V}}_K^{(t)})_{\mathcal{J}_i} - (\mathbf{V}_K)_{\mathcal{J}_i}\mathbf{W}\big\|_{\mathrm{op}} \\ &\le |\mathcal{J}_i|^{1/2}\mathcal{T}\big(\widehat{\mathbf{V}}_K^{(t)}, \mathbf{V}_K\big) \le |\mathcal{J}_i|^{1/2}\rho^t\Delta.\end{aligned}$$

Now, for $i \in \mathcal{I}$,

$$\begin{aligned}\sigma_K\big((\widehat{\mathbf{V}}_K^{(t)})_{\mathcal{J}_i}\big) &\ge \sigma_K\big((\mathbf{V}_K)_{\mathcal{J}_i}\big) - \big|\sigma_K\big((\widehat{\mathbf{V}}_K^{(t)})_{\mathcal{J}_i}\big) - \sigma_K\big((\mathbf{V}_K)_{\mathcal{J}_i}\big)\big| \\ &\ge \big(\sigma_*^{-1} + \epsilon - \sqrt{d}\Delta\big)(|\mathcal{J}_i|/d)^{1/2}.\end{aligned}$$

On the other hand, if $i \in \mathcal{I}^{\mathrm{c}}$ and $\|\boldsymbol{\omega}_i\|_1 > K$, then

$$\begin{aligned}\sigma_K\big((\widehat{\mathbf{V}}_K^{(t)})_{\mathcal{J}_i}\big) &\le \sigma_K\big((\mathbf{V}_K)_{\mathcal{J}_i}\big) + |\sigma_K\big((\widehat{\mathbf{V}}_K^{(t)})_{\mathcal{J}_i}\big) - \sigma_K\big((\mathbf{V}_K)_{\mathcal{J}_i}\big)| \\ &\le \big(\sigma_*^{-1} - \epsilon + \sqrt{d}\Delta\big)(|\mathcal{J}_i|/d)^{1/2}.\end{aligned}$$

Hence, if we choose $c_1 \le \epsilon$, then $\sqrt{d}\Delta < \epsilon$, so for $i \in \mathcal{I}$,

$$\sigma_K\big((\widehat{\mathbf{V}}_K^{(t)})_{\mathcal{J}_i}\big) > \left(\frac{|\mathcal{J}_i|}{d\sigma_*}\right)^{1/2};$$

moreover, for $i \in \mathcal{I}^{\mathrm{c}}$,

$$\sigma_K\big((\widehat{\mathbf{V}}_K^{(t)})_{\mathcal{J}_i}\big) < \left(\frac{|\mathcal{J}_i|}{d\sigma_*}\right)^{1/2}.$$

Claim (a) follows. As for claim (b), note that $\widehat{\mathbf{V}}_K^{(t+1)} = \texttt{refine}(K, \widehat{\mathbf{V}}_K^{(t)}, \mathbf{\Omega}_{\mathcal{I}^{(t)}}, (\mathbf{Y}_{\mathbf{\Omega}})_{\mathcal{I}^{(t)}})$. Taking $c_1, C > 0$ from Proposition 1, and reducing c_1 if necessary so that $c_1 \le \epsilon$, we may apply this proposition to deduce that whenever

(i) $\mathcal{T}(\widehat{\mathbf{V}}_K^{(t)}, \mathbf{V}_K) \le \frac{c_1\sigma_K(\mathbf{\Gamma})}{K^2\mu^4\sigma_1(\mathbf{\Gamma})\sqrt{d}}$;

(ii) $\rho := \frac{CK^2\mu^4\sigma_1(\mathbf{\Gamma})\|\mathbf{\Omega}_{\mathcal{I}}^{\mathrm{c}}\|_{1\to1}}{\sigma_K(\mathbf{\Gamma})|\mathcal{I}|} < 1$,

we have $\mathcal{T}(\widehat{\mathbf{V}}_K^{(t+1)}, \mathbf{V}_K) \leq \rho\mathcal{T}(\widehat{\mathbf{V}}_K^{(t)}, \mathbf{V}_K)$. But the conditions (i) and (ii) are ensured by the inductive hypothesis and our assumptions, so the conclusion follows.

It is convenient to prove Proposition 2 before Theorem 4.

Proof (of Proposition 2). In this proof, we use the shorthand $\mathbf{D}_\mathbf{u} := \mathrm{diag}(\mathbf{u})$ for $u \in \mathbb{R}^d$. We represent $\widetilde{\mathbf{G}}$ under the orthonormal basis $(\mathbf{V}_K, \mathbf{V}_{-K})$ as follows:

$$\widetilde{\mathbf{G}} = (\mathbf{V}_K, \mathbf{V}_{-K})\begin{pmatrix} \mathbf{V}_K^\top\widetilde{\mathbf{G}}\mathbf{V}_K & \mathbf{V}_K^\top\widetilde{\mathbf{G}}\mathbf{V}_{-K} \\ \mathbf{V}_{-K}^\top\widetilde{\mathbf{G}}\mathbf{V}_K & \mathbf{V}_{-K}^\top\widetilde{\mathbf{G}}\mathbf{V}_{-K} \end{pmatrix}\begin{pmatrix} \mathbf{V}_K^\top \\ \mathbf{V}_{-K}^\top \end{pmatrix}.$$

Define

$$\mathbf{G}^* := (\mathbf{V}_K, \mathbf{V}_{-K})\begin{pmatrix} \mathbf{V}_K^\top\widetilde{\mathbf{G}}\mathbf{V}_K & \mathbf{0} \\ \mathbf{0} & \mathbf{V}_{-K}^\top\widetilde{\mathbf{G}}\mathbf{V}_{-K} \end{pmatrix}\begin{pmatrix} \mathbf{V}_K^\top \\ \mathbf{V}_{-K}^\top \end{pmatrix}.$$

In the sequel, we regard $\widetilde{\mathbf{G}}$ as a corrupted version of $\mathbf{G}^*$ with the off-diagonal blocks $\mathbf{V}_K^\top\widetilde{\mathbf{G}}\mathbf{V}_{-K}$ and $\mathbf{V}_{-K}^\top\widetilde{\mathbf{G}}\mathbf{V}_K$ as perturbations. We have

$$\|\mathbf{V}_K^\top\widetilde{\mathbf{G}}\mathbf{V}_{-K}\|_\mathrm{F} = \|\mathbf{V}_K^\top(\widetilde{\mathbf{G}} - \mathbf{\Sigma}_\mathbf{y})\mathbf{V}_{-K}\|_\mathrm{F} \leq \|\mathbf{V}_K^\top(\widetilde{\mathbf{G}} - \mathbb{E}^{\mathbf{\Omega}}\widetilde{\mathbf{G}})\|_\mathrm{F}$$

We control the right-hand side through a concentration inequality, and for $k \in [K]$ let $\mathbf{v}_k$ denote the kth column of $\mathbf{V}_K$. For any $j \in [d]$ and $k \in [K]$,

$$\begin{aligned}\mathbf{v}_k^\top(\widetilde{\mathbf{G}} - \mathbb{E}^{\mathbf{\Omega}}\widetilde{\mathbf{G}})\mathbf{e}_j &= \frac{1}{n}\sum_{i=1}^n \mathbf{v}_k^\top\bigl\{\widetilde{\mathbf{y}}_i\widetilde{\mathbf{y}}_i^\top \circ \widetilde{\mathbf{W}} - \mathbb{E}^{\mathbf{\Omega}}\bigl(\widetilde{\mathbf{y}}_i\widetilde{\mathbf{y}}_i^\top \circ \widetilde{\mathbf{W}}\bigr)\bigr\}\mathbf{e}_j \\ &= \frac{1}{n}\sum_{i=1}^n \bigl\{\widetilde{\mathbf{y}}_i^\top\mathbf{D}_{\mathbf{v}_k}\widetilde{\mathbf{W}}\mathbf{D}_{\mathbf{e}_j}\widetilde{\mathbf{y}}_i - \mathbb{E}^{\mathbf{\Omega}}\bigl(\widetilde{\mathbf{y}}_i^\top\mathbf{D}_{\mathbf{v}_k}\widetilde{\mathbf{W}}\mathbf{D}_{\mathbf{e}_j}\widetilde{\mathbf{y}}_i\bigr)\bigr\} \\ &= \frac{1}{n}\sum_{i=1}^n \bigl\{\widetilde{y}_{ij}\widetilde{\mathbf{y}}_i^\top\mathbf{D}_{\mathbf{v}_k}\widetilde{\mathbf{W}}_j - \mathbb{E}^{\mathbf{\Omega}}\bigl(\widetilde{y}_{ij}\widetilde{\mathbf{y}}_i^\top\mathbf{D}_{\mathbf{v}_k}\widetilde{\mathbf{W}}_j\bigr)\bigr\},\end{aligned} \tag{12}$$

where $\widetilde{\mathbf{W}}_j$ denotes the jth column of $\widetilde{\mathbf{W}}$.

Note that

$$\|\mathbf{y}_i\|_{\psi_2^*} \leq \sup_{\mathbf{v}\in\mathcal{S}^{d-1}} \frac{\|\mathbf{v}^\top\mathbf{V}_K\mathbf{u}_i\|_{\psi_2} + \|\mathbf{v}^\top\mathbf{z}_i\|_{\psi_2}}{\sqrt{\mathbf{v}^\top\mathbf{V}_K\mathbf{\Sigma}_\mathbf{u}\mathbf{V}_K^\top\mathbf{v} + 1}} \leq 2\tau.$$

Thus for any vector $\mathbf{a} \in \mathbb{R}^d$, we have by Lemma 1 that

$$\|y_{ij}(\mathbf{a}^\top\mathbf{y}_i)\|_{\psi_1} \leq 2\|y_{ij}\|_{\psi_2}\|\mathbf{a}^\top\mathbf{y}_i\|_{\psi_2} \leq 4\tau(M\mathbf{a}^\top\mathbf{\Sigma}_\mathbf{y}\mathbf{a})^{1/2}.$$

For $i \in [n]$, let $\mathbf{a}_i := \omega_{ij}\widetilde{\mathbf{W}}_j \circ \mathbf{v}_k \circ \boldsymbol{\omega}_i$. Now for any $q \geq 2$,

$$\begin{aligned}\mathbb{E}^{\mathbf{\Omega}}\bigl|\widetilde{y}_{ij}(\widetilde{\mathbf{W}}_j^\top\mathbf{D}_{\mathbf{v}_k}\widetilde{\mathbf{y}}_i)\bigr|^q = \mathbb{E}^{\mathbf{\Omega}}|y_{ij}\mathbf{a}_i^\top\mathbf{y}_i|^q &\leq \Bigl(4q\tau\sqrt{M\mathbf{a}_i^\top\mathbf{\Sigma}_\mathbf{y}\mathbf{a}_i}\Bigr)^q \\ &\leq \frac{16q^q\tau^2\mu^2KMR}{d}\Bigl(4\tau\mu\sqrt{KMR\|\widetilde{\mathbf{W}}_j\|_2^2/d}\Bigr)^{q-2}\sum_{t=1}^d \widetilde{W}_{tj}^2\omega_{it}\omega_{ij} \\ &\leq \frac{8e^2q!\tau^2\mu^2KMR}{d}\Bigl(4e\tau\mu\sqrt{KMR\|\widetilde{\mathbf{W}}_j\|_2^2/d}\Bigr)^{q-2}\sum_{t=1}^d \widetilde{W}_{tj}^2\omega_{it}\omega_{ij},\end{aligned}$$

where the penultimate inequality uses the fact that $\|\mathbf{a}_i\|_2^2 \leq K\mu^2\|\widetilde{\mathbf{W}}_j\|_2^2/d$, and the last inequality is due to Stirling's approximation.

Hence,

$$\begin{aligned}\frac{1}{n}\sum_{i=1}^{n}\mathbb{E}^{\mathbf{\Omega}}|\widetilde{y}_{ij}\widetilde{\mathbf{W}}_j^\top\mathbf{D}_{\mathbf{v}_k}\widetilde{\mathbf{y}}_i|^q &\leq \frac{8e^2q!\tau^2\mu^2KMR}{d}\Bigl(4e\tau\mu\sqrt{KMR\|\widetilde{\mathbf{W}}_j\|_2^2/d}\Bigr)^{q-2}\sum_{t=1}^{d}\sum_{i=1}^{n}\frac{\widetilde{W}_{jt}^2\omega_{it}\omega_{ij}}{n}\\ &= \frac{8e^2q!\tau^2\mu^2KMR}{d}\Bigl(4e\tau\mu\sqrt{KMR\|\widetilde{\mathbf{W}}_j\|_2^2/d}\Bigr)^{q-2}\|\widetilde{\mathbf{W}}_j\|_1.\end{aligned}$$

Thus by (12) and Bernstein's inequality (Boucheron, Lugosi and Massart, 2013, Theorem 2.10), we have that for any $\xi > 0$,

$$\mathbb{P}^{\mathbf{\Omega}}\biggl\{\bigl|\mathbf{v}_k^\top(\widetilde{\mathbf{G}} - \mathbb{E}^{\mathbf{\Omega}}\widetilde{\mathbf{G}})\mathbf{e}_j\bigr| \geq 2^{5/2}e\tau\mu\Bigl(\frac{KMR}{d}\Bigr)^{1/2}\biggl(\Bigl(\frac{\xi\|\widetilde{\mathbf{W}}_j\|_1}{n}\Bigr)^{1/2} + \frac{\xi\|\widetilde{\mathbf{W}}_j\|_2}{n}\biggr)\biggr\} \leq 2e^{-\xi}. \tag{13}$$

By a union bound over $(j,k) \in [d]\times[K]$, for any $\xi > 1$,

$$\begin{aligned}\mathbb{P}^{\mathbf{\Omega}}\biggl\{\|\mathbf{V}_K^\top\widetilde{\mathbf{G}}\mathbf{V}_{-K}\|_{\mathrm{F}} \geq 8eK\tau\mu\Bigl(\frac{MR}{d}\Bigr)^{1/2}\Bigl(\frac{\xi^{1/2}\|\widetilde{\mathbf{W}}\|_1^{1/2}\log^{1/2}d}{n^{1/2}} + \frac{\xi\|\widetilde{\mathbf{W}}\|_{\mathrm{F}}\log d}{n}\Bigr)\biggr\}&\\ \leq 2Kd^{-(\xi-1)}.&\end{aligned} \tag{14}$$

Now we provide a condition under which $\lambda_{\min}(\mathbf{V}_K^\top\widetilde{\mathbf{G}}\mathbf{V}_K) > \|\mathbf{V}_{-K}^\top\widetilde{\mathbf{G}}\mathbf{V}_{-K}\|_{\mathrm{op}}$, which ensures that $\mathbf{V}_K$ is the top K eigenspace of $\mathbf{G}^*$. Note that

$$\lambda_{\min}(\mathbf{V}_K^\top\widetilde{\mathbf{G}}\mathbf{V}_K) \geq \lambda_K + 1 - \|\mathbf{V}_K^\top(\widetilde{\mathbf{G}} - \mathbf{\Sigma}_{\mathbf{y}})\mathbf{V}_K\|_{\mathrm{op}} \geq \lambda_K + 1 - \|\widetilde{\mathbf{G}} - \mathbf{\Sigma}_{\mathbf{y}}\|_{\mathrm{op}}$$

and

$$\|\mathbf{V}_{-K}^\top\widetilde{\mathbf{G}}\mathbf{V}_{-K}\|_{\mathrm{op}} \leq 1 + \|\widetilde{\mathbf{G}} - \mathbf{\Sigma}_{\mathbf{y}}\|_{\mathrm{op}}.$$

This implies that if $\lambda_K > 4\|\widetilde{\mathbf{G}} - \mathbf{\Sigma}_{\mathbf{y}}\|_{\mathrm{op}}$, then

$$\lambda_{\min}(\mathbf{V}_K^\top\widetilde{\mathbf{G}}\mathbf{V}_K) - \|\mathbf{V}_{-K}^\top\widetilde{\mathbf{G}}\mathbf{V}_{-K}\|_{\mathrm{op}} > \lambda_K/2. \tag{15}$$

In the following, we derive an exponential tail bound for $\|\widetilde{\mathbf{G}} - \mathbf{\Sigma}_{\mathbf{y}}\|_{\mathrm{op}} = \|\widetilde{\mathbf{G}} - \mathbb{E}^{\mathbf{\Omega}}\widetilde{\mathbf{G}}\|_{\mathrm{op}}$. Let $\mathbf{A}_i := \widetilde{\mathbf{y}}_i\widetilde{\mathbf{y}}_i^\top \circ \widetilde{\mathbf{W}}$ and note that $\|\mathbf{A}_i\|_{\mathrm{op}} \leq \|\mathbf{y}_i\|_\infty^2\|\widetilde{\mathbf{W}}\|_{\mathrm{op}}$. Recalling the definition of the matrix absolute value§, for any $\mathbf{v} = (v_1,\ldots,v_d)^\top \in \mathcal{S}^{d-1}$ and any integer $q \geq 2$, we have

$$\begin{aligned}\mathbb{E}^{\mathbf{\Omega}}\bigl(\mathbf{v}^\top|\mathbf{A}_i|^q\mathbf{v}\bigr) &\leq \mathbb{E}^{\mathbf{\Omega}}\bigl(\bigl\|\mathbf{A}_i\bigr\|_{\mathrm{op}}^{q-2}\mathbf{v}^\top\mathbf{A}_i^2\mathbf{v}\bigr) \leq \mathbb{E}^{\mathbf{\Omega}}\bigl\{\bigl(\|\widetilde{\mathbf{W}}\|_{\mathrm{op}}\|\mathbf{y}_i\|_\infty^2\bigr)^{q-2}\mathbf{v}^\top\bigl(\widetilde{\mathbf{y}}_i\widetilde{\mathbf{y}}_i^\top \circ \widetilde{\mathbf{W}}\bigr)^2\mathbf{v}\bigr\}\\ &= \|\widetilde{\mathbf{W}}\|_{\mathrm{op}}^{q-2}\mathbb{E}^{\mathbf{\Omega}}\bigl\{\|\mathbf{y}_i\|_\infty^{2(q-2)}\mathbf{v}^\top\mathbf{D}_{\widetilde{\mathbf{y}}_i}\widetilde{\mathbf{W}}\mathbf{D}_{\widetilde{\mathbf{y}}_i}\mathbf{D}_{\widetilde{\mathbf{y}}_i}\widetilde{\mathbf{W}}\mathbf{D}_{\widetilde{\mathbf{y}}_i}\mathbf{v}\bigr\}\\ &= \|\widetilde{\mathbf{W}}\|_{\mathrm{op}}^{q-2}\mathbb{E}^{\mathbf{\Omega}}\bigl\{\|\mathbf{y}_i\|_\infty^{2(q-2)}\,\mathrm{tr}(\mathbf{D}_{\widetilde{\mathbf{y}}_i}^2\widetilde{\mathbf{W}}\mathbf{D}_{\widetilde{\mathbf{y}}_i}\mathbf{v}\mathbf{v}^\top\mathbf{D}_{\widetilde{\mathbf{y}}_i}\widetilde{\mathbf{W}})\bigr\}\\ &= \|\widetilde{\mathbf{W}}\|_{\mathrm{op}}^{q-2}\sum_{j=1}^{d}\omega_{ij}\mathbb{E}^{\mathbf{\Omega}}\bigl\{y_{ij}^2\|\mathbf{y}_i\|_\infty^{2(q-2)}\bigl(\widetilde{\mathbf{W}}_j^\top\mathbf{D}_{\mathbf{v}}\widetilde{\mathbf{y}}_i\bigr)^2\bigr\}.\end{aligned}$$

Now, for each $j \in [d]$, and $q \geq 2$,

$$\begin{aligned}\mathbb{E}^{\mathbf{\Omega}}\bigl\{y_{ij}^2\|\mathbf{y}_i\|_\infty^{2(q-2)}(\widetilde{\mathbf{W}}_j^\top\mathbf{D}_{\mathbf{v}}\widetilde{\mathbf{y}}_i)^2\bigr\} &= \mathbb{E}^{\mathbf{\Omega}}\bigl[y_{ij}^2\|\mathbf{y}_i\|_\infty^{2(q-2)}\{(\widetilde{\mathbf{W}}_j \circ \mathbf{v} \circ \boldsymbol{\omega}_i)^\top\mathbf{y}_i\}^2\bigr]\\ &\leq \bigl(\mathbb{E}y_{ij}^8\bigr)^{1/4}\bigl\{\mathbb{E}\bigl(\|\mathbf{y}_i\|_\infty^{8(q-2)}\bigr)\bigr\}^{1/4}8R\tau^2\|\widetilde{\mathbf{W}}_j \circ \mathbf{v} \circ \boldsymbol{\omega}_i\|_2^2\\ &\lesssim MR\tau^2\{8(q-2)CM\log d\}^{q-2}\sum_{t=1}^{d}(v_t\widetilde{W}_{tj}\omega_{it})^2,\end{aligned}$$

§This is defined formally just before Lemma 3.

where the last inequality is due to the fact that $\big\|\|\mathbf{y}_i\|_\infty\big\|_{\psi_2} \leq (CM\log d)^{1/2}$ by (2). Therefore,

$$\begin{aligned}\sum_{i=1}^n \mathbb{E}^{\mathbf{\Omega}}\big(\mathbf{v}^\top|\mathbf{A}_i|^q\mathbf{v}\big) &\lesssim MR\tau^2\big\{8(q-2)CM\|\widetilde{\mathbf{W}}\|_{\mathrm{op}}\log d\big\}^{q-2}\sum_{j,t=1}^d\sum_{i=1}^n \omega_{ij}\omega_{it}v_t^2\widetilde{W}_{tj}^2 \\ &= nMR\tau^2\big\{8(q-2)CM\|\widetilde{\mathbf{W}}\|_{\mathrm{op}}\log d\big\}^{q-2}\sum_{j,t=1}^d v_t^2\widetilde{W}_{tj} \\ &\lesssim q!nMR\tau^2\|\widetilde{\mathbf{W}}^\top\|_{1\to1}\big(8eCM\|\widetilde{\mathbf{W}}\|_{\mathrm{op}}\log d\big)^{q-2},\end{aligned}$$

where $\|\widetilde{\mathbf{W}}^\top\|_{1\to1} = \sup_{\|\mathbf{u}\|_1=1}\|\widetilde{\mathbf{W}}^\top\mathbf{u}\|_1 = \|\widetilde{\mathbf{W}}\|_{1\to1}$. Since the above inequality holds for all $\mathbf{v}\in\mathcal{S}^{d-1}$, we have

$$\Big\|\sum_{i=1}^n \mathbb{E}^{\mathbf{\Omega}}\big(|\mathbf{A}_i|^q\big)\Big\|_{\mathrm{op}} \lesssim q!nMR\tau^2\|\widetilde{\mathbf{W}}\|_{1\to1}\big(8eCM\|\widetilde{\mathbf{W}}\|_{\mathrm{op}}\log d\big)^{q-2}.$$

By a version of the Matrix Bernstein inequality for non-central absolute moments, which we give as Lemma 3, there exists a universal constant $C_1>0$ such that for any $\xi>1$,

$$\mathbb{P}^{\mathbf{\Omega}}\bigg\{\big\|\widetilde{\mathbf{G}} - \mathbb{E}^{\mathbf{\Omega}}\widetilde{\mathbf{G}}\big\|_{\mathrm{op}} \geq C_1\bigg(\Big(\frac{MR\tau^2\|\widetilde{\mathbf{W}}\|_{1\to1}\xi\log d}{n}\Big)^{1/2} + \frac{M\|\widetilde{\mathbf{W}}\|_{\mathrm{op}}\xi\log^2 d}{n}\bigg)\bigg\} \leq 4d^{-(\xi-1)}. \tag{16}$$

Now let

$$\mathcal{A} := \Big\{\lambda_{\min}(\mathbf{V}_K^\top\widetilde{\mathbf{G}}\mathbf{V}_K) - \|\mathbf{V}_{-K}^\top\widetilde{\mathbf{G}}\mathbf{V}_{-K}\|_{\mathrm{op}} > \frac{\lambda_K}{2}\Big\}.$$

From (15) and (16), we deduce that for any $\xi>1$, if

$$\lambda_K \geq 4C_1\bigg\{\Big(\frac{MR\tau^2\|\widetilde{\mathbf{W}}\|_{1\to1}\xi\log d}{n}\Big)^{1/2} + \frac{M\|\widetilde{\mathbf{W}}\|_{\mathrm{op}}\xi\log^2 d}{n}\bigg\}, \tag{17}$$

then $\mathbb{P}^{\mathbf{\Omega}}(\mathcal{A}^{\mathrm{c}}) \leq \mathbb{P}^{\mathbf{\Omega}}\{\|\widetilde{\mathbf{G}} - \mathbf{\Sigma}_{\mathbf{y}}\|_{\mathrm{op}} \geq \lambda_K/4\} \leq 4d^{-(\xi-1)}$. The desired result follows immediately by combining this with (14) and applying Yu, Wang and Samworth (2015, Theorem 2).

Proof (of Theorem 4). Let $\mathbf{E} := \widetilde{\mathbf{G}} - \mathbb{E}^{\mathbf{\Omega}}\widetilde{\mathbf{G}} = \widetilde{\mathbf{G}} - \mathbf{\Sigma}_{\mathbf{y}}$. By Cape, Tang and Priebe (2018, Theorem 3.7), when $\lambda_K \geq 2\|\mathbf{E}\|_{\mathrm{op}}$, we have that

$$\begin{aligned}\mathcal{T}(\widetilde{\mathbf{V}}_K,\mathbf{V}_K) \leq\;& 2\lambda_K^{-1}\|\mathbf{V}_{-K}\mathbf{V}_{-K}^\top\mathbf{E}\mathbf{V}_K\mathbf{V}_K^\top\|_{2\to\infty} \\ &+ 2\lambda_K^{-1}\|\mathbf{V}_{-K}\mathbf{V}_{-K}^\top\mathbf{E}\mathbf{V}_{-K}\mathbf{V}_{-K}^\top\|_{2\to\infty}\|\sin\Theta(\widetilde{\mathbf{V}}_K,\mathbf{V}_K)\|_{\mathrm{op}} \\ &+ 2\lambda_K^{-1}\|\mathbf{V}_{-K}\mathbf{V}_{-K}^\top\mathbf{\Sigma}_{\mathbf{y}}\mathbf{V}_{-K}\mathbf{V}_{-K}^\top\|_{2\to\infty}\|\sin\Theta(\widetilde{\mathbf{V}}_K,\mathbf{V}_K)\|_{\mathrm{op}} \\ &+ \|\sin\Theta(\widetilde{\mathbf{V}}_K,\mathbf{V}_K)\|_{\mathrm{op}}^2\|\mathbf{V}_K\|_{2\to\infty} \\ =:\;& T_1+T_2+T_3+T_4.\end{aligned}$$

Note that if λ_K satisfies (17) for some $\xi>1$, then $\mathbb{P}^{\mathbf{\Omega}}(\|\mathbf{E}\|_{\mathrm{op}} \geq \lambda_K/4) \leq 4d^{-(\xi-1)}$. In fact, since $\|\widetilde{\mathbf{W}}\|_{\mathrm{op}} \leq \|\widetilde{\mathbf{W}}\|_{\mathrm{F}}$, there exists $c_{M,\tau}>0$ such that (10) in the main text implies (17), which, together with (16) ensures that $\mathbb{P}^{\mathbf{\Omega}}(\|\mathbf{E}\|_{\mathrm{op}} \geq \lambda_K/2) \leq 4d^{-(\xi-1)}$.

To bound T_1, we have

$$\begin{aligned}\|\mathbf{V}_{-K}\mathbf{V}_{-K}^\top\mathbf{E}\mathbf{V}_K\mathbf{V}_K^\top\|_{2\to\infty} &\leq \|\mathbf{V}_{-K}\mathbf{V}_{-K}^\top\|_{\infty\to\infty}\|\mathbf{E}\mathbf{V}_K\mathbf{V}_K^\top\|_{2\to\infty} \\ &\leq (1+K\mu)\max_{j\in[d]}\sup_{\mathbf{u}\in\mathcal{S}^{K-1}}\mathbf{e}_j^\top\mathbf{E}\mathbf{V}_K\mathbf{u},\end{aligned} \tag{18}$$

where the second inequality is due to the fact that

$$\begin{aligned}\|\mathbf{V}_{-K}\mathbf{V}_{-K}^\top\|_{\infty\to\infty} &\leq \|\mathbf{I}_d\|_{\infty\to\infty} + \|\mathbf{V}_K\mathbf{V}_K^\top\|_{\infty\to\infty} \leq 1 + \|\mathbf{V}_K\|_{\infty\to\infty}\|\mathbf{V}_K^\top\|_{\infty\to\infty} \\ &\leq 1 + K^{1/2}\|\mathbf{V}_K\|_{2\to\infty}\cdot d^{1/2}\|\mathbf{V}_K^\top\|_{2\to\infty} \leq 1+K\mu.\end{aligned}$$

We use a covering argument to bound the supremum term. Let $\mathcal{N}_K(1/2)$ be a 1/2-net of the Euclidean sphere $\mathcal{S}^{K-1}$, i.e., for any $\mathbf{u} \in \mathcal{S}^{K-1}$, there exists a point $\pi(\mathbf{u}) \in \mathcal{N}_K(1/2)$ such that $\|\mathbf{u}-\pi(\mathbf{u})\|_2 \leq 1/2$. Note that for any $\mathbf{u} \in \mathcal{S}^{K-1}$,

$$\mathbf{e}_j^\top \mathbf{E}\mathbf{V}_K\mathbf{u} = \mathbf{e}_j^\top \mathbf{E}\mathbf{V}_K\pi(\mathbf{u}) + \mathbf{e}_j^\top \mathbf{E}\mathbf{V}_K(\mathbf{u}-\pi(\mathbf{u})) \leq \max_{\mathbf{v}\in\mathcal{N}_K(1/2)} \mathbf{e}_j^\top \mathbf{E}\mathbf{V}_K\mathbf{v} + \frac{1}{2}\sup_{\mathbf{v}\in\mathcal{S}^{K-1}} \mathbf{e}_j^\top \mathbf{E}\mathbf{V}_K\mathbf{v},$$

which further implies that

$$\sup_{\mathbf{u}\in\mathcal{S}^{K-1}} \mathbf{e}_j^\top \mathbf{E}\mathbf{V}_K\mathbf{u} \leq 2 \max_{\mathbf{u}\in\mathcal{N}_K(1/2)} \mathbf{e}_j^\top \mathbf{E}\mathbf{V}_K\mathbf{u}. \tag{19}$$

We then argue similarly as in (13), with $\mathbf{V}_K\mathbf{u}$ taking the role of $\mathbf{v}_k$ there (since $\|\mathbf{V}_K\mathbf{u}\|_\infty \leq \mu(K/d)^{1/2}$) to obtain that for any $\xi > 0$ and $\mathbf{u} \in \mathcal{N}_K(1/2)$,

$$\mathbb{P}^{\mathbf{\Omega}}\biggl\{|\mathbf{e}_j^\top \mathbf{E}\mathbf{V}_K\mathbf{u}| \geq 2^{5/2}e\tau\mu\Bigl(\frac{KMR}{d}\Bigr)^{1/2}\Bigl(\frac{\xi^{1/2}\|\widetilde{\mathbf{W}}_j\|_1^{1/2}}{n^{1/2}} + \frac{\xi\|\widetilde{\mathbf{W}}_j\|_2}{n}\Bigr)\biggr\} \leq e^{-\xi}.$$

By Vershynin (2012, Lemma 5.2), $|\mathcal{N}_K(1/2)| \leq 5^K$. Hence, by (18), (19) and a union bound, we have for any $\xi > \log 5$ that

$$\mathbb{P}^{\mathbf{\Omega}}\biggl\{T_1 \geq \frac{2^{9/2}e\tau\mu(1+K\mu)}{\lambda_K}\Bigl(\frac{KMR}{d}\Bigr)^{1/2}\Bigl(\frac{\xi^{1/2}\|\widetilde{\mathbf{W}}\|_{\infty\to\infty}^{1/2}}{n^{1/2}} + \frac{\xi\|\widetilde{\mathbf{W}}\|_{2\to\infty}}{n}\Bigr)\biggr\} \leq de^{K\log 5-\xi}.$$

Next we bound T_2. Note that

$$\|\mathbf{V}_{-K}\mathbf{V}_{-K}^\top\mathbf{E}\mathbf{V}_{-K}\mathbf{V}_{-K}^\top\|_{2\to\infty} \leq \|\mathbf{V}_{-K}\mathbf{V}_{-K}^\top\|_{\infty\to\infty}\|\mathbf{E}\|_{2\to\infty} \leq (1+K\mu)\|\mathbf{E}\|_{2\to\infty}.$$

For $j,k \in [d]$, let $\mathcal{I}_{jk} := \{i : \omega_{ij}\omega_{ik} = 1\}$ and $n_{jk} := |\mathcal{I}_{jk}| = n/\widetilde{W}_{jk}$. Then

$$E_{jk} = \frac{1}{n}\sum_{i=1}^n \widetilde{y}_{ij}\widetilde{y}_{ik}\widetilde{W}_{jk} - [\mathbb{E}^{\mathbf{\Omega}}\widetilde{\mathbf{G}}]_{jk} = \frac{1}{n_{jk}}\sum_{i\in\mathcal{I}_{jk}} y_{ij}y_{ik} - [\mathbb{E}^{\mathbf{\Omega}}\widetilde{\mathbf{G}}]_{jk}.$$

By applying both parts of Lemma 1, for any $i \in [n]$ and $j,k \in [d]$, we have that $\|y_{ij}y_{ik}\|_{\psi_1} \leq 2\|y_{ij}\|_{\psi_2}\|y_{ik}\|_{\psi_2} \leq 2M$. Applying Bernstein's inequality (Boucheron, Lugosi and Massart, 2013, Theorem 2.10) yields that for any $\xi > 0$,

$$\mathbb{P}^{\mathbf{\Omega}}\biggl\{|E_{jk}| \geq 2eM\biggl(\Bigl(\frac{2\xi\widetilde{W}_{jk}}{n}\Bigr)^{1/2} + \frac{\xi\widetilde{W}_{jk}}{n}\biggr)\biggr\} \leq 2e^{-\xi}.$$

Therefore, a union bound with $(j,k) \in [d]\times[d]$ yields that

$$\mathbb{P}^{\mathbf{\Omega}}\biggl\{T_2 \geq \frac{4\sqrt{2}eM(1+K\mu)}{\lambda_K}\biggl(\Bigl(\frac{2\xi\|\widetilde{\mathbf{W}}\|_{\infty\to\infty}}{n}\Bigr)^{1/2} + \frac{\xi\|\widetilde{\mathbf{W}}\|_{2\to\infty}}{n}\biggr)\|\sin\Theta(\widetilde{\mathbf{V}}_K,\mathbf{V}_K)\|_{\mathrm{op}}\biggr\} \leq 2d^2e^{-\xi}.$$

Now we bound T_3. We have that

$$T_3 = \frac{2\|\mathbf{V}_{-K}\mathbf{V}_{-K}^\top\|_{2\to\infty}}{\lambda_K}\|\sin\Theta(\widetilde{\mathbf{V}}_K,\mathbf{V}_K)\|_{\mathrm{op}} \leq \frac{2\{1+\mu(K/d)^{1/2}\}}{\lambda_K}\|\sin\Theta(\widetilde{\mathbf{V}}_K,\mathbf{V}_K)\|_{\mathrm{op}}.$$

Finally, T_4 satisfies

$$T_4 \leq \frac{\mu K^{1/2}}{d^{1/2}}\|\sin\Theta(\widetilde{\mathbf{V}}_K,\mathbf{V}_K)\|_{\mathrm{op}}^2.$$

Since $\|\sin\Theta(\widetilde{\mathbf{V}}_K,\mathbf{V}_K)\|_{\mathrm{op}} \leq \min\bigl\{L(\widetilde{\mathbf{V}}_K,\mathbf{V}_K),1\bigr\}$, combining our bounds for $\{T_j\}_{j=1}^4$ yields that there exists $C_{M,\tau} > 0$ such that for any $\xi > 2$,

$$\begin{aligned}\mathbb{P}^{\mathbf{\Omega}}\biggl\{\mathcal{T}(\widetilde{\mathbf{V}}_K,\mathbf{V}_K) \geq \frac{K\mu C_{M,\tau}}{\lambda_K}\biggl\{L(\widetilde{\mathbf{V}}_K,\mathbf{V}_K) + \mu\Bigl(\frac{KR}{d}\Bigr)^{1/2}\biggr\}\Bigl(\frac{\xi^{1/2}\|\widetilde{\mathbf{W}}\|_{\infty\to\infty}^{1/2}}{n^{1/2}} + \frac{\xi\|\widetilde{\mathbf{W}}\|_{2\to\infty}}{n}\Bigr)\\ + \mu\Bigl(\frac{K^{1/2}}{d^{1/2}} + \frac{4}{\lambda_K}\Bigr)L(\widetilde{\mathbf{V}}_K,\mathbf{V}_K)\biggr\} \leq de^{K\log 5-\xi} + 2d^2e^{-\xi} + 4d^{-(\xi-1)}.\end{aligned}$$

It therefore follows from Proposition 2 in the main text, which applies because condition (10) in the main text for a suitable $c_{M,\tau}$ implies (11) in the main text, together with the facts that $\|\widetilde{\mathbf{W}}\|_1 \leq d\|\widetilde{\mathbf{W}}\|_{\infty\to\infty}$ and $\|\widetilde{\mathbf{W}}\|_{\mathrm{F}} \leq d^{1/2}\|\widetilde{\mathbf{W}}\|_{2\to\infty}$, that the first conclusion of the theorem holds. The second conclusion then follows immediately.

Proof (of Proposition 3). As an abbreviation, we write $\widehat{\mathbf{V}}_K := \widehat{\mathbf{V}}_K^{(\mathrm{in})}$. For any $i \in [n]$, define $\widehat{\mathbf{B}}^{(i)} := (\widehat{\mathbf{V}}_{\mathcal{J}_i,K}^\top \widehat{\mathbf{V}}_{\mathcal{J}_i,K})^{-1}\widehat{\mathbf{V}}_{\mathcal{J}_i,K}^\top \mathbf{\Xi}_{\mathcal{J}_i} \in \mathbb{R}^{K\times K}$. Note that

$$\begin{aligned}\|\widehat{\mathbf{B}}^{(i)}\|_{\mathrm{op}} &\leq \frac{\sigma_*^2 d\|\widehat{\mathbf{V}}_{\mathcal{J}_i,K}^\top \mathbf{\Xi}_{\mathcal{J}_i}\|_{\mathrm{op}}}{|\mathcal{J}_i|} \leq \frac{\sigma_*^2 d\big(\|\mathbf{V}_{\mathcal{J}_i,K}^\top \mathbf{\Xi}_{\mathcal{J}_i}\|_{\mathrm{op}} + \|\mathbf{\Xi}_{\mathcal{J}_i}\|_{\mathrm{op}}^2\big)}{|\mathcal{J}_i|} \\ &\leq \sigma_*^2(\kappa_1+\kappa_2)\|\mathbf{\Xi}\|_{\mathrm{op}}^2 + \sigma_*^2\kappa_1\frac{\mu K(\log K)^{1/2}\|\mathbf{\Xi}\|_{\mathrm{op}}}{d^{1/2}} =: M.\end{aligned}$$

Now define $\widetilde{\mathbf{Y}} := \mathbf{Y} - (\widehat{\mathbf{B}}^{(1)}\mathbf{O}^\top\mathbf{u}_1,\ldots,\widehat{\mathbf{B}}^{(n)}\mathbf{O}^\top\mathbf{u}_n)^\top\mathbf{O}^\top\mathbf{V}_K^\top$, and let $\mathbf{E} := \widehat{\mathbf{Y}} - \widetilde{\mathbf{Y}}$. For any $i \in [n]$, we have

$$\mathbf{e}_{i,\mathcal{J}_i} = (\widehat{\mathbf{y}}_i - \widetilde{\mathbf{y}}_i)_{\mathcal{J}_i} = \mathbf{V}_{\mathcal{J}_i,K}\mathbf{O}\widehat{\mathbf{B}}^{(i)}\mathbf{O}^\top\mathbf{u}_i.$$

Now, for $i \in [n]$, define $\widetilde{\mathbf{u}}_i := (\mathbf{I}_K - \mathbf{O}\widehat{\mathbf{B}}^{(i)}\mathbf{O}^\top)\mathbf{u}_i$. Then

$$\begin{aligned}\mathbf{e}_{i,\mathcal{J}_i^c} = (\widehat{\mathbf{y}}_i - \widetilde{\mathbf{y}}_i)_{\mathcal{J}_i^c} &= \widehat{\mathbf{V}}_{\mathcal{J}_i^c,K}(\widehat{\mathbf{V}}_{\mathcal{J}_i,K}^\top\widehat{\mathbf{V}}_{\mathcal{J}_i,K})^{-1}\widehat{\mathbf{V}}_{\mathcal{J}_i,K}^\top\mathbf{y}_{i,\mathcal{J}_i} - \mathbf{V}_{\mathcal{J}_i^c,K}\widetilde{\mathbf{u}}_i \\ &= \widehat{\mathbf{V}}_{\mathcal{J}_i^c,K}(\widehat{\mathbf{V}}_{\mathcal{J}_i,K}^\top\widehat{\mathbf{V}}_{\mathcal{J}_i,K})^{-1}\widehat{\mathbf{V}}_{\mathcal{J}_i,K}^\top(\widehat{\mathbf{V}}_{\mathcal{J}_i,K} - \mathbf{\Xi}_{\mathcal{J}_i})\mathbf{O}^\top\mathbf{u}_i - \mathbf{V}_{\mathcal{J}_i^c,K}\widetilde{\mathbf{u}}_i \\ &= \big(\widehat{\mathbf{V}}_{\mathcal{J}_i^c,K} - \widehat{\mathbf{V}}_{\mathcal{J}_i^c,K}\widehat{\mathbf{B}}^{(i)} - \mathbf{V}_{\mathcal{J}_i^c,K}\mathbf{O} + \mathbf{V}_{\mathcal{J}_i^c,K}\mathbf{O}\widehat{\mathbf{B}}^{(i)}\big)\mathbf{O}^\top\mathbf{u}_i \\ &= \mathbf{\Xi}_{\mathcal{J}_i^c}(\mathbf{I}_K - \widehat{\mathbf{B}}^{(i)})\mathbf{O}^\top\mathbf{u}_i = \mathbf{\Xi}_{\mathcal{J}_i^c}\mathbf{O}^\top\widetilde{\mathbf{u}}_i.\end{aligned}$$

Now, by Weyl's inequality, we have that

$$\sigma_K(\widetilde{\mathbf{Y}}) \geq \sigma_K(\mathbf{Y}) - \big\|(\widehat{\mathbf{B}}^{(1)}\mathbf{O}^\top\mathbf{u}_1,\ldots,\widehat{\mathbf{B}}^{(n)}\mathbf{O}^\top\mathbf{u}_n)\big\|_{\mathrm{F}} \geq \sigma_K(\mathbf{Y}) - M\|\mathbf{Y}\|_{\mathrm{F}}.$$

By Theorem 1.4 of Wang (2016), there exists $\widehat{\mathbf{O}} \in \mathbb{O}^{K\times K}$ such that

$$\begin{aligned}\|\widehat{\mathbf{V}}_K^{\mathrm{out}} - \mathbf{V}_K\widehat{\mathbf{O}}\|_{\mathrm{F}} &\leq \frac{8}{\sigma_K(\mathbf{Y}) - M\|\mathbf{Y}\|_{\mathrm{F}}}\bigg\{\sum_{i\in[n]}\big(\|\mathbf{u}_i\|_2^2\|\widehat{\mathbf{B}}^{(i)}\|_{\mathrm{op}}^2 + \|\widetilde{\mathbf{u}}_i\|_2^2\|\mathbf{\Xi}_{\mathcal{J}_i^c}\|_{\mathrm{op}}^2\big)\bigg\}^{1/2} \\ &\leq \frac{8}{(c-M)\|\mathbf{Y}\|_{\mathrm{F}}}\bigg\{\bigg(\sum_{i\in[n]}\|\mathbf{u}_i\|_2^2\|\widehat{\mathbf{B}}^{(i)}\|_{\mathrm{op}}^2\bigg)^{1/2} + \bigg(\sum_{i\in[n]}\|\widetilde{\mathbf{u}}_i\|_2^2\|\mathbf{\Xi}_{\mathcal{J}_i^c}\|_{\mathrm{op}}^2\bigg)^{1/2}\bigg\} \\ &\leq \frac{8\big\{M + \kappa_3^{1/2}(1+M)\|\mathbf{\Xi}\|_{\mathrm{op}}\big\}}{c-M}.\end{aligned}$$

When $\|\mathbf{\Xi}\|_{\mathrm{op}} \leq \min\big\{\big(\frac{c}{4\sigma_*^2(\kappa_1+\kappa_2)}\big)^{1/2}, \frac{c}{4\mu\kappa_1\sigma_*^2}\big(\frac{d}{K\log K}\big)^{1/2}\big\}$, we have $M \leq c/2$. Thus

$$\begin{aligned}\|\widehat{\mathbf{V}}_K^{\mathrm{out}} - \mathbf{V}_K\widehat{\mathbf{O}}\|_{\mathrm{op}} &\leq \|\widehat{\mathbf{V}}_K^{\mathrm{out}} - \mathbf{V}_K\widehat{\mathbf{O}}\|_{\mathrm{F}} \\ &\leq \frac{16\|\mathbf{\Xi}\|_{\mathrm{op}}}{c}\bigg\{\sigma_*^2(\kappa_1+\kappa_2)\|\mathbf{\Xi}\|_{\mathrm{op}} + \sigma_*^2\kappa_1\mu K\bigg(\frac{\log K}{d}\bigg)^{1/2} + \kappa_3^{1/2}\bigg(1+\frac{c}{2}\bigg)\bigg\},\end{aligned}$$

as required.

Proof (of Corollary 1). Under the p-homogeneous MCAR missingness mechanism, we have for any $i \in [n]$ that

$$\mathbb{E}(\mathbf{V}_{\mathcal{J}_i,K}^\top\mathbf{\Xi}_{\mathcal{J}_i}) = p\mathbf{V}_K^\top\mathbf{\Xi} = -(p/2)\mathbf{O}\mathbf{\Xi}^\top\mathbf{\Xi} \quad \text{and} \quad \mathbb{E}(\mathbf{\Xi}_{\mathcal{J}_i}^\top\mathbf{\Xi}_{\mathcal{J}_i}) = p\mathbf{\Xi}^\top\mathbf{\Xi}.$$

For $j \in [d]$, let $\mathbf{v}_j^\top \in \mathbb{R}^K$ and $\boldsymbol{\xi}_j^\top \in \mathbb{R}^K$ denote the jth rows of $\mathbf{V}_K$ and $\boldsymbol{\Xi}$ respectively. Then $\mathbf{V}_{\mathcal{J}_i,K}^\top \boldsymbol{\Xi}_{\mathcal{J}_i} = \sum_{j=1}^d \omega_{ij}\mathbf{v}_j\boldsymbol{\xi}_j^\top$, and for $q = 2, 3, \ldots$,

$$\mathbb{E}\big((\omega_{ij}\mathbf{v}_j\boldsymbol{\xi}_j^\top\boldsymbol{\xi}_j\mathbf{v}_j^\top)^{q/2}\big) \preceq p\|\mathbf{v}_j\|_2^q\|\boldsymbol{\xi}_j\|_2^q\mathbf{I}_K \preceq \frac{p\mu^2K\|\boldsymbol{\xi}_j\|_2^2}{d}\bigg\{\mu\bigg(\frac{K}{d}\bigg)^{1/2}\|\boldsymbol{\Xi}\|_{\mathrm{op}}\bigg\}^{q-2}\mathbf{I}_K.$$

Similarly,

$$\mathbb{E}\big((\omega_{ij}\boldsymbol{\xi}_j\mathbf{v}_j^\top\mathbf{v}_j\boldsymbol{\xi}_j^\top)^{q/2}\big) \preceq \frac{p\mu^2K\|\boldsymbol{\xi}_j\|_2^2}{d}\bigg\{\mu\bigg(\frac{K}{d}\bigg)^{1/2}\|\boldsymbol{\Xi}\|_{\mathrm{op}}\bigg\}^{q-2}\mathbf{I}_K.$$

Applying Corollary 3 therefore gives that for every $t > 0$ and $i \in [n]$,

$$\mathbb{P}\bigg(\bigg\|\mathbf{V}_{\mathcal{J}_i,K}^\top\boldsymbol{\Xi}_{\mathcal{J}_i} + \frac{p}{2}\mathbf{O}\boldsymbol{\Xi}^\top\boldsymbol{\Xi}\bigg\|_{\mathrm{op}} \geq t\bigg) \leq 8K\exp\bigg(\frac{-t^2/32}{p\mu^2K\|\boldsymbol{\Xi}\|_{\mathrm{F}}^2/d + \mu(K/d)^{1/2}\|\boldsymbol{\Xi}\|_{\mathrm{op}}t/3}\bigg).$$

Thus, for any $\delta \in (0,1]$, with probability at least $1 - \delta/3$, we have

$$\|\mathbf{V}_{\mathcal{J}_i,K}^\top\boldsymbol{\Xi}_{\mathcal{J}_i}\|_{\mathrm{op}} \leq \frac{p}{2}\|\boldsymbol{\Xi}\|_{\mathrm{op}}^2 + 22\|\boldsymbol{\Xi}\|_{\mathrm{op}}\frac{\mu K\log(24K/\delta)}{d^{1/2}}. \tag{20}$$

In addition, $\boldsymbol{\Xi}_{\mathcal{J}_i}^\top\boldsymbol{\Xi}_{\mathcal{J}_i} = \sum_{j=1}^d \omega_{ij}\boldsymbol{\xi}_j\boldsymbol{\xi}_j^\top$, and $\mathbb{E}\big((\omega_{ij}\boldsymbol{\xi}_j\boldsymbol{\xi}_j^\top)^q\big) \preceq p\|\boldsymbol{\xi}_j\|_2^{2q}\mathbf{I}_K$ for $q = 2, 3, \ldots$. Applying Lemma 3 yields that for all $t > 0$ and $i \in [n]$,

$$\begin{aligned}\mathbb{P}\big(\|\boldsymbol{\Xi}_{\mathcal{J}_i}^\top\boldsymbol{\Xi}_{\mathcal{J}_i} - p\boldsymbol{\Xi}^\top\boldsymbol{\Xi}\|_{\mathrm{op}} \geq t\big) &\leq 4K\exp\bigg(\frac{-t^2/32}{p\sum_{j=1}^d\|\boldsymbol{\xi}_j\|_2^4 + \|\boldsymbol{\Xi}\|_{\mathrm{op}}^2t/3}\bigg)\\ &\leq 4K\exp\bigg(\frac{-t^2/32}{p\|\boldsymbol{\Xi}\|_{\mathrm{F}}^2\|\boldsymbol{\Xi}\|_{\mathrm{op}}^2 + \|\boldsymbol{\Xi}\|_{\mathrm{op}}^2t/3}\bigg).\end{aligned}$$

Thus, for any $\delta \in (0,1]$, with probability at least $1 - \delta/3$, we have

$$\big\|\boldsymbol{\Xi}_{\mathcal{J}_i}\big\|_{\mathrm{op}}^2 \leq 22K^{1/2}\|\boldsymbol{\Xi}\|_{\mathrm{op}}^2\log(12K/\delta). \tag{21}$$

By the multiplicative Chernoff bound, when $dp \geq 8\log(3/\delta)$, we have

$$\mathbb{P}(|\mathcal{J}_i| < dp/2) \leq e^{-dp/8} \leq \delta/3. \tag{22}$$

On the other hand, we have by the usual Bernstein's inequality that

$$\begin{aligned}\mathbb{P}\big(\|\boldsymbol{\Xi}_{\mathcal{J}_i^c}\|_{\mathrm{F}}^2 > (1-p)\|\boldsymbol{\Xi}\|_{\mathrm{F}}^2 + t\big) &\leq \exp\bigg(\frac{-t^2/2}{p(1-p)\sum_{j=1}^d\|\boldsymbol{\xi}_j\|_2^4 + \|\boldsymbol{\Xi}\|_{2\to\infty}^2t/3}\bigg)\\ &\leq \exp\bigg(\frac{-t^2/2}{K(1-p)\|\boldsymbol{\Xi}\|_{\mathrm{op}}^4/C_*^2 + \|\boldsymbol{\Xi}\|_{\mathrm{op}}^2t/(3C_*^2)}\bigg),\end{aligned}$$

where the last step uses the fact that $\|\boldsymbol{\Xi}\|_{2\to\infty} \leq \|\boldsymbol{\Xi}\|_{\mathrm{op}}/C_*$. Thus, for any $\delta \in (0,1]$, with probability at least $1 - \delta/3$, we have that

$$\begin{aligned}\|\boldsymbol{\Xi}_{\mathcal{J}_i^c}\|_{\mathrm{op}}^2 &\leq K(1-p)\|\boldsymbol{\Xi}\|_{\mathrm{op}}^2 + \frac{2}{C_*}\{K(1-p)\log(3/\delta)\}^{1/2}\|\boldsymbol{\Xi}\|_{\mathrm{op}}^2 + \frac{4}{3C_*^2}\|\boldsymbol{\Xi}\|_{\mathrm{op}}^2\log(3/\delta)\\ &\leq \bigg\{2K(1-p) + \frac{7}{3C_*^2}\log(3/\delta)\bigg\}\|\boldsymbol{\Xi}\|_{\mathrm{op}}^2.\end{aligned}$$

Combining (20), (21) and (22) with a union bound, we see that in Proposition 3, if we take

$$\begin{aligned}\kappa_1 &= \frac{44}{p}K\|\boldsymbol{\Xi}\|_{\mathrm{op}}\log(24nK/\delta), \quad \kappa_2 = \frac{44}{p}K^{1/2}\|\boldsymbol{\Xi}\|_{\mathrm{op}}^2\log(12nK/\delta)\\ \kappa_3 &= 2K(1-p) + \frac{7\log(3n/\delta)}{3C_*^2},\end{aligned}$$

then the conditions (12) of that proposition hold simultaneously with probability at least $1-\delta$. Moreover, since $\|\boldsymbol{\Xi}\|_{\mathrm{op}} \le p/\bigl(44K\log(24nK/\delta)\bigr)$, we have $\kappa_2 \le \kappa_1 \le 1$, and hence condition (13) of Proposition 3 is also satisfied. Therefore, by Proposition 3, we have

$$\|\widehat{\mathbf{V}}_K^{(\mathrm{out})} - \mathbf{V}_K\widehat{\mathbf{O}}\|_{\mathrm{op}} \le \frac{16}{c}\bigl(3\kappa_1\mu K\sigma_*^2 + 2\kappa_3^{1/2}\bigr)\|\boldsymbol{\Xi}\|_{\mathrm{op}} \le \frac{80}{c}\kappa_3^{1/2}\|\widehat{\mathbf{V}}_K^{(\mathrm{in})} - \mathbf{V}_K\widehat{\mathbf{O}}\|_{\mathrm{op}},$$

where we used the fact that $\|\boldsymbol{\Xi}\|_{\mathrm{op}} \le \frac{p(1-p)^{1/2}}{22\sqrt{2}\mu K^{3/2}\sigma_*^2\log(24nK/\delta)}$ in the final bound.

B. Auxiliary lemmas used in Section A

LEMMA 1. *Let X and Y be two sub-Gaussian random variables. Then we have $\|X\|_{\psi_2}^2 \le \|X^2\|_{\psi_1}$ and $\|XY\|_{\psi_1} \le 2\|X\|_{\psi_2}\|Y\|_{\psi_2}$.*

PROOF. For any $x \ge 0$, let $\lceil x\rceil := \inf\{z \in \mathbb{N} : z \ge x\}$. According to the definitions of the ψ_1-norm and ψ_2-norm, we have that

$$\|X\|_{\psi_2}^2 = \sup_{p\in\mathbb{N}} \frac{\mathbb{E}(|X|^p)^{2/p}}{p} \le \sup_{p\in\mathbb{N}} \frac{\bigl\{\mathbb{E}\bigl(X^{2\lceil p/2\rceil}\bigr)\bigr\}^{\frac{1}{\lceil p/2\rceil}}}{p} \le \|X^2\|_{\psi_1},$$

where the penultimate inequality is due to Jensen's inequality and the last inequality is due to the fact that $p \ge \lceil p/2\rceil$. For the second inequality,

$$\begin{aligned}\|XY\|_{\psi_1} &= \sup_{p\in\mathbb{N}} \frac{(\mathbb{E}|XY|^p)^{1/p}}{p} \le 2\sup_{p\in\mathbb{N}} \frac{(\mathbb{E}|X|^{2p})^{1/(2p)}}{\sqrt{2p}}\frac{(\mathbb{E}|Y|^{2p})^{1/(2p)}}{\sqrt{2p}} \\ &\le 2\sup_{p\in\mathbb{N}} \frac{(\mathbb{E}|X|^{2p})^{1/(2p)}}{\sqrt{2p}} \sup_{q\in\mathbb{N}} \frac{(\mathbb{E}|Y|^{2q})^{1/(2q)}}{\sqrt{2q}} \le 2\|X\|_{\psi_2}\|Y\|_{\psi_2},\end{aligned}$$

as required.

LEMMA 2. *If $X_1,\ldots,X_n$ are independent centred random variables with $\max_{i\in[n]}\|X_i\|_{\psi_1} < \infty$, then there exists a universal constant $C > 0$ such that*

$$\biggl\|\sum_{i=1}^n X_i\biggr\|_{\psi_1} \le C\biggl(\sum_{i=1}^n \|X_i\|_{\psi_1}^2\biggr)^{1/2}.$$

PROOF. Write $K_i := \|X_i\|_{\psi_1}$ and $\mathbf{K} := (K_1,\ldots,K_n)^\top$. From Vershynin (2012, Lemma 5.15), there exist universal constants $c_1, C_1 > 0$ such that for $|t| \le c_1/\|\mathbf{K}\|_\infty$,

$$\mathbb{E}\exp\biggl\{t\sum_{i=1}^n X_i\biggr\} = \prod_{i=1}^n \mathbb{E}\exp\{tX_i\} \le \exp\bigl\{C_1t^2\|\mathbf{K}\|_2^2\bigr\}.$$

Setting $t = \min\{C_1^{-1/2}\|\mathbf{K}\|_2^{-1}, c_1\|\mathbf{K}\|_\infty^{-1}\}$ in the above expression, the right-hand side is bounded above by e. The desired result follows from the fact that (5.15) and (5.16) in Vershynin (2012) are two definitions that yield equivalent ψ_1-norms.

The following lemma provides a variant of the existing matrix Bernstein inequality (Tropp, 2012, Theorem 6.2). The primary difference is that we impose non-central absolute moment inequalities, as opposed to central moment inequalities. We believe that this inequality may be of independent interest, with applications beyond the scope of this paper. To state the result, for any symmetric matrix $\mathbf{A} \in \mathbb{R}^{d\times d}$ with eigendecomposition $\mathbf{Q}\,\mathrm{diag}(\mu_1,\ldots,\mu_d)\mathbf{Q}^\top$, where $\mathbf{Q} \in \mathbb{O}^{d\times d}$, we define its *matrix absolute value* as $|\mathbf{A}| := \mathbf{Q}\,\mathrm{diag}(|\mu_1|,\ldots,|\mu_d|)\mathbf{Q}^\top$.

LEMMA 3 (A MATRIX BERNSTEIN INEQUALITY WITH NON-CENTRAL MOMENT CONDITIONS). *Let* $\{\mathbf{X}_i\}_{i\in[n]}$ *be independent symmetric* $d \times d$ *random matrices. Assume that*

$$\mathbb{E}\big(|\mathbf{X}_i|^q\big) \preceq \frac{q!}{2} R^{q-2}\mathbf{A}_i^2 \quad \textit{for } q = 2, 3, 4, \ldots$$

for some $R > 0$ *and deterministic* d*-dimensional symmetric matrices* $\{\mathbf{A}_i\}_{i\in[n]}$*. Define the variance parameter*

$$\sigma^2 := \bigg\|\sum_{i=1}^n \mathbf{A}_i^2\bigg\|_{\mathrm{op}}.$$

Then for each $t > 0$*,*

$$\mathbb{P}\bigg[\lambda_{\max}\bigg\{\sum_{i=1}^n (\mathbf{X}_i - \mathbb{E}\mathbf{X}_i)\bigg\} \geq t\bigg] \leq 4d \exp\bigg(\frac{-t^2/32}{\sigma^2 + Rt}\bigg).$$

PROOF. Let $\widetilde{\mathbf{X}}_1, \ldots, \widetilde{\mathbf{X}}_n, \epsilon_1, \ldots, \epsilon_n$ be independent random matrices and variables, independent of $(\mathbf{X}_1, \ldots, \mathbf{X}_n)$, satisfying $\widetilde{\mathbf{X}}_i \overset{d}{=} \mathbf{X}_i$ and $\epsilon_i \sim U(\{-1, 1\})$ for $i \in [n]$. Write $\mathbf{S}_n := \sum_{i=1}^n (\mathbf{X}_i - \mathbb{E}\mathbf{X}_i)$ and $\widetilde{\mathbf{S}}_n := \sum_{i=1}^n (\widetilde{\mathbf{X}}_i - \mathbb{E}\mathbf{X}_i)$. Given $\mathbf{X}_1, \ldots, \mathbf{X}_n$, let $\mathbf{v}_* = \mathbf{v}_*(\mathbf{X}_1, \ldots, \mathbf{X}_n)$ be a leading unit-length eigenvector of $\mathbf{S}_n$. Let $\tilde{\mathbf{v}}_1, \ldots, \tilde{\mathbf{v}}_d$ denote orthonormal eigenvectors of $\widetilde{\mathbf{X}}_1$ with corresponding eigenvalues $\tilde{\mu}_1, \ldots, \tilde{\mu}_d$; fix $\mathbf{v} \in \mathcal{S}^{d-1}$, and let $w_j := (\tilde{\mathbf{v}}_j^\top \mathbf{v})^2$ for $j \in [d]$. Since $\sum_{j=1}^d w_j = 1$, we have by Jensen's inequality that for $q \in \{2, 3, \ldots\}$,

$$|\mathbf{v}^\top \widetilde{\mathbf{X}}_1 \mathbf{v}|^q = \bigg|\sum_{j=1}^d w_j \tilde{\mu}_j\bigg|^q \leq \sum_{j=1}^d w_j |\tilde{\mu}_j|^q = \mathbf{v}^\top |\widetilde{\mathbf{X}}_1|^q \mathbf{v}.$$

We deduce that $\mathbb{E}\big\{(\mathbf{v}^\top \widetilde{\mathbf{X}}_i \mathbf{v})_+^q\big\} \leq \mathbb{E}\big\{|\mathbf{v}^\top \widetilde{\mathbf{X}}_i \mathbf{v}|^q\big\} \leq \frac{q!}{2} R^{q-2} \mathbf{v}^\top \mathbf{A}_i^2 \mathbf{v}$ for $i \in [n]$, so by Bernstein's inequality (Boucheron, Lugosi and Massart, 2013, Corollary 2.11),

$$\mathbb{P}\big(\mathbf{v}_*^\top \widetilde{\mathbf{S}}_n \mathbf{v}_* > t/2 \bigm| \mathbf{X}_1, \ldots, \mathbf{X}_n\big) \leq \exp\bigg(\frac{-t^2/8}{\mathbf{v}_*^\top \sum_{i=1}^n \mathbf{A}_i^2 \mathbf{v}_* + Rt}\bigg) \leq \exp\bigg(\frac{-t^2/8}{\sigma^2 + Rt}\bigg).$$

We may assume that the right-hand side of the above inequality is at most $1/2$, since otherwise the lemma is trivially true. Therefore,

$$\begin{aligned}
\mathbb{P}\{\lambda_{\max}(\mathbf{S}_n) \geq t\} &= \mathbb{P}(\mathbf{v}_*^\top \mathbf{S}_n \mathbf{v}_* \geq t) \leq 2\mathbb{E}\big\{\mathbb{P}\big(\mathbf{v}_*^\top \widetilde{\mathbf{S}}_n \mathbf{v}_* \leq t/2 \bigm| \mathbf{X}_1, \ldots, \mathbf{X}_n\big)\mathbb{1}_{\{\mathbf{v}_*^\top \mathbf{S}_n \mathbf{v}_* \geq t\}}\big\} \\
&= 2\mathbb{P}\big(\mathbf{v}_*^\top \widetilde{\mathbf{S}}_n \mathbf{v}_* \leq t/2 \text{ and } \mathbf{v}_*^\top \mathbf{S}_n \mathbf{v}_* \geq t\big) \leq 2\mathbb{P}\big(\mathbf{v}_*^\top (\mathbf{S}_n - \widetilde{\mathbf{S}}_n) \mathbf{v}_* \geq t/2\big) \\
&\leq 2\mathbb{P}\bigg[\lambda_{\max}\bigg\{\sum_{i=1}^n \epsilon_i (\mathbf{X}_i - \widetilde{\mathbf{X}}_i)\bigg\} \geq t/2\bigg] \leq 4\mathbb{P}\bigg\{\lambda_{\max}\bigg(\sum_{i=1}^n \epsilon_i \mathbf{X}_i\bigg) \geq t/4\bigg\}, \qquad (23)
\end{aligned}$$

where we have used the fact that $\epsilon_i(\mathbf{X}_i - \widetilde{\mathbf{X}}_i) \overset{d}{=} \mathbf{X}_i - \widetilde{\mathbf{X}}_i$ for all i in the penultimate inequality.

Since $\mathbb{E}(\epsilon_i \mathbf{X}_i) = \mathbf{0}$ and $\mathbb{E}\big\{(\epsilon_i \mathbf{X}_i)^q\big\} \preceq \mathbb{E}(|\mathbf{X}_i|^q) \preceq \frac{q!}{2} R^{q-2} \mathbf{A}_i^2$ for $q \in \{2, 3, \ldots\}$, applying the matrix Bernstein inequality (Tropp, 2012, Theorem 6.2) to the sequence $\{\epsilon_i \mathbf{X}_i\}_{i\in[n]}$ yields

$$\mathbb{P}\bigg\{\lambda_{\max}\bigg(\sum_{i=1}^n \epsilon_i \mathbf{X}_i\bigg) \geq t/4\bigg\} \leq d \exp\bigg(\frac{-t^2/32}{\sigma^2 + Rt}\bigg).$$

We attain the conclusion by combining the above inequality with (23).

LEMMA 4. *Let* $X_1, \ldots, X_n$ *be independent* $\mathrm{Bin}(d, p)$ *random variables and let* $\widehat{p}_i := X_i/d$*. When* $dp \geq 1$ *and* $n \geq 2$*, we have*

$$\mathbb{E} \max_{i\in[n]} \widehat{p}_i \leq 10p \log n.$$

PROOF. By Bernstein's inequality (van der Vaart and Wellner, 1996, Lemma 2.2.9) and a union bound,

$$\mathbb{P}\Bigl(\max_{i\in[n]} \widehat{p}_i \geq p + t\Bigr) \leq n \exp\biggl(-\frac{dt^2}{2(p+t/3)}\biggr).$$

Setting $t_0 := 2\sqrt{pd^{-1}\log n} + \frac{4}{3d}\log n$, we have

$$\mathbb{E}\max_{i\in[n]} \widehat{p}_i = p + t_0 + \int_{t_0}^{\infty} n\{e^{-dt^2/(4p)} + e^{-3dt/4}\}\,dt \leq p + t_0 + \sqrt{\frac{\pi p}{d}} + \frac{4}{3d} \leq 10p\log n,$$

where we have used $\log n \geq \log 2$ and $1/d \leq p$ in the final inequality.

The following lemma controls the Kullback–Leibler divergence between two centred multivariate normal distributions.

LEMMA 5. *Suppose that $\boldsymbol{\beta}, \boldsymbol{\eta} \in \mathbb{R}^d$ and $\|\boldsymbol{\eta}\|_2 = \|\boldsymbol{\beta}\|_2$. Let $\boldsymbol{\Sigma}_1 := \mathbf{I}_d + \boldsymbol{\beta}\boldsymbol{\beta}^\top$ and $\boldsymbol{\Sigma}_2 := \mathbf{I}_d + \boldsymbol{\eta}\boldsymbol{\eta}^\top$. Then*

$$\mathrm{KL}\bigl(N_d(\mathbf{0}, \boldsymbol{\Sigma}_1), N_d(\mathbf{0}, \boldsymbol{\Sigma}_2)\bigr) = \frac{\|\boldsymbol{\eta}\|_2^4 - (\boldsymbol{\eta}^\top\boldsymbol{\beta})^2}{2(1+\|\boldsymbol{\eta}\|_2^2)}.$$

PROOF. Since $\|\boldsymbol{\eta}\|_2 = \|\boldsymbol{\beta}\|_2$, the matrices $\boldsymbol{\Sigma}_1$ and $\boldsymbol{\Sigma}_2$ share the same set of eigenvalues. Hence $\det \boldsymbol{\Sigma}_1 = \det \boldsymbol{\Sigma}_2$ and we have

$$\mathrm{KL}\bigl(N_d(\mathbf{0}, \boldsymbol{\Sigma}_1), N_d(\mathbf{0}, \boldsymbol{\Sigma}_2)\bigr) = \frac{1}{2}\bigl\{\mathrm{tr}\bigl(\boldsymbol{\Sigma}_2^{-1}\boldsymbol{\Sigma}_1\bigr) - d\bigr\} = \frac{1}{2}\bigl\{\mathrm{tr}\bigl((\mathbf{I}_d + \boldsymbol{\eta}\boldsymbol{\eta}^\top)^{-1}(\mathbf{I}_d + \boldsymbol{\beta}\boldsymbol{\beta}^\top)\bigr) - d\bigr\}.$$

Now, by the Sherman–Morrison formula,

$$(\mathbf{I}_d + \boldsymbol{\eta}\boldsymbol{\eta}^\top)^{-1} = \mathbf{I}_d - \frac{\boldsymbol{\eta}\boldsymbol{\eta}^\top}{1+\|\boldsymbol{\eta}\|_2^2}$$

and thus we have

$$\begin{aligned}\mathrm{KL}\bigl(N_d(\mathbf{0}, \boldsymbol{\Sigma}_1), N_d(\mathbf{0}, \boldsymbol{\Sigma}_2)\bigr) &= \frac{1}{2}\biggl[\mathrm{tr}\biggl(\biggl(\mathbf{I}_d - \frac{\boldsymbol{\eta}\boldsymbol{\eta}^\top}{1+\|\boldsymbol{\eta}\|_2^2}\biggr)(\mathbf{I}_d + \boldsymbol{\beta}\boldsymbol{\beta}^\top)\biggr) - d\biggr] \\ &= \frac{1}{2}\biggl(\|\boldsymbol{\beta}\|_2^2 - \frac{\|\boldsymbol{\eta}\|_2^2}{1+\|\boldsymbol{\eta}\|_2^2} - \frac{(\boldsymbol{\eta}^\top\boldsymbol{\beta})^2}{1+\|\boldsymbol{\eta}\|_2^2}\biggr) = \frac{\|\boldsymbol{\eta}\|_2^4 - (\boldsymbol{\eta}^\top\boldsymbol{\beta})^2}{2(1+\|\boldsymbol{\eta}\|_2^2)},\end{aligned}$$

as required.

Theorem 4 and Proposition 2 in the main text exhibit bounds on $\mathcal{T}(\widetilde{\mathbf{V}}_K, \mathbf{V}_K)$ and $L(\widetilde{\mathbf{V}}_K, \mathbf{V}_K)$ given a deterministic observation scheme. The following lemma derives probabilistic bounds for various norms of $\widetilde{\mathbf{W}}$.

LEMMA 6. *Let $\boldsymbol{\Omega} = (\omega_{ij}) \in \{0,1\}^{n\times d}$ have independent and identically distributed rows, and write $p_{jk} := \mathbb{E}(\omega_{1j}\omega_{1k})$ for $j,k \in [d]$. Define $\mathbf{W} = (W_{jk}) \in [0,\infty]^{d\times d}$ by $W_{jk} := 1/p_{jk}$, and let $\widetilde{\mathbf{W}} = (\widetilde{W}_{jk})_{j,k\in[d]}$ be defined as in (9). Then there exists an event of probability at least $1 - \sum_{j,k\in[d]} e^{-np_{jk}/8}$ on which each of the following inequalities holds:*

(i) $\|\widetilde{\mathbf{W}}\|_{\infty\to\infty} \leq 2\|\mathbf{W}\|_{\infty\to\infty} = 2\max_{j\in[d]}\sum_{k\in[d]} 1/p_{jk}$;
(ii) $\|\widetilde{\mathbf{W}}\|_1 \leq 2\|\mathbf{W}\|_1 = 2\sum_{j,k\in[d]} 1/p_{jk}$;
(iii) $\|\widetilde{\mathbf{W}}\|_{\mathrm{F}} \leq 2\|\mathbf{W}\|_{\mathrm{F}} = 2\bigl(\sum_{j,k\in[d]} 1/p_{jk}^2\bigr)^{1/2}$;
(iv) $\|\widetilde{\mathbf{W}}\|_{2\to\infty} \leq 2\|\mathbf{W}\|_{2\to\infty} = 2\max_{j\in[d]}\bigl(\sum_{k\in[d]} 1/p_{jk}^2\bigr)^{1/2}$.

PROOF. Define the event

$$\mathcal{A} := \Bigl\{\max_{j,k\in[d]} \widetilde{W}_{jk}/W_{jk} \leq 2\Bigr\}.$$

For $j, k \in [d]$, write $\widehat{p}_{jk} := n^{-1} \sum_{i=1}^{n} \omega_{ij}\omega_{ik}$. Then by a union bound and the multiplicative form of Bernstein's inequality (McDiarmid, 1998, Theorem 2.3(c)), we have

$$\mathbb{P}(\mathcal{A}^{\mathrm{c}}) \leq \sum_{j=1}^{d}\sum_{k=1}^{d} \mathbb{P}\bigl(\widehat{p}_{jk} < p_{jk}/2\bigr) \leq \sum_{j=1}^{d}\sum_{k=1}^{d} e^{-np_{jk}/8}.$$

The desired bounds on the event $\mathcal{A}$ then follow from the definitions of the norms.

References

Boucheron, S., Lugosi, G. and Massart, P. (2013) *Concentration Inequalities: A Nonasymptotic Theory of Independence.* Oxford University Press, Oxford.

Cai, T. T. and Zhang, A. (2018a) Rate-optimal perturbation bounds for singular subspaces with applications to high-dimensional statistics. *Ann. Statist.*, **46**, 60–89.

Cape, J., Tang, M. and Priebe, C. E. (2018) The two-to-infinity norm and singular subspace geometry with applications to high-dimensional statistics. *Ann. Statist.*, to appear.

Massart, P. (2007) *Concentration Inequalities and Model Selection*, Springer, Berlin.

McDiarmid, C. (1998) Concentration. In *Probabilistic Methods for Algorithmic Discrete Mathematics*, pp. 195–248, Springer.

Tropp, J. A. (2012) User-friendly tail bounds for sums of random matrices. *Found. Comput. Math.*, **12**, 389–434.

van der Vaart, A. W. and Wellner, J. A. (1996) *Weak Convergence and Empirical Processes.* Springer, New York.

Vershynin, R. (2012) Introduction to the non-asymptotic analysis of random matrices. In Y. Eldar and G. Kutyniok (Eds.) *Compressed Sensing, Theory and Applications.* Cambridge University Press, Cambridge. 210–26.

Yu, B. (1997) Assouad, Fano and Le Cam. In *Festschrift for Lucien Le Cam: Research Papers in Probability and Statistics*, Pollard, D., Torgersen, E. and Yang G. L. (Eds.), 423–435. Springer, New York.

Yu, Y., Wang, T. and Samworth, R. J. (2015) A useful variant of the Davis–Kahan theorem for statisticians. *Biometrika*, **102**, 315–323.

Zhu, Z., Wang, T. and Samworth, R. J. (2021) High-dimensional principal component analysis with heterogeneous missingness. *Submitted.*

Wang, T. (2016) Spectral methods and computational trade-offs in high-dimensional statistical inference. Ph.D. thesis, University of Cambridge.
